# New insights from broadband simulations into small overmoded smooth and corrugated terahertz waveguides and transitions for NMR-DNP

**DOI:** 10.1016/j.jmro.2020.100009

**Published:** 2021-01-16

**Authors:** F David Doty, Glenn N. Doty, John P. Staab, Yuriy Sizyuk, Paul D. Ellis

**Affiliations:** Doty Scientific, Columbia SC, United States

**Keywords:** Overmoded Waveguides (OMWGs), Downtapers, NMR-DNP, Corrugated wave guides

## Abstract

The primary impetus for the work reported in this paper is to develop efficient overmoded waveguides (OMWGs) that employ broadband downtaper transitions that would be compatible with the severe space constraints in high-field NB magnets. Further, it is essential these would be readily manufacturable, as high precision corrugated metallic downtapers for the sub-mmw regime are very difficult to produce. We have simulated numerous alternatives to corrugated circular OMWGs, including most of the previously reported alternatives (except for many of the low-power fiberoptics options) and several novel designs. We conclude that corrugated circular metallic OMWGs are the best of the reported options to date (except from a cost perspective) for diameters down to ~1.5*λ*, but the corrugation parameters for small OMWGs need to be significantly different from the previously published guidelines that have worked well for large OMWGs. With numerically optimized small OMWGs, easily manufacturable smooth downtapers appear to work as well as corrugated downtapers in many cases relevant to MAS-DNP probes. Our example simulations will be for the 170–230 GHz range, but the lessons and results should be readily applicable to other ranges by simple scaling.

## 1. Introduction

Resonance frequencies for ^1^H’s in nuclear magnetic resonance (NMR) spectroscopy have historically been in the range of 100 MHz up to perhaps as high as 1.5 GHz. More typically the frequencies are in the range of 500 to 900 MHz. As an NMR probe designer, one is always focused on the optimized means of coupling the rf energy to the sample and then obtaining the highest performance of the detected signal. Dynamic Nuclear Polarization (DNP) experiments have changed this paradigm dramatically. This experiment includes resonant electron irradiation at the same high magnetic field used for the NMR experiment. Given the size of the magnetic moment of the electron, the electron resonance frequency then becomes hundreds of GHz. From the perspective of a manufacturer of NMR probes capable of DNP experiments, the challenges increase many-fold, and one of the most significant is the efficient coupling of the resonant electron irradiation to the NMR sample. From a background in rf-electronics, behavior of microwaves can be counterintuitive in many respects. Likewise, the terminology associated with millimeter waves (mmw) can be unfamiliar. As the frequency of interest increases from several hundred MHz to many tens of GHz, the normal co-axial cable becomes an inefficient means to transmit electromagnetic energy between two points. The higher frequencies demand a different means of transmission and these are denoted as waveguides, which have conventionally been simple hollow rectangular or circular tubes of high conductivity metals. The theory for energy transfer in waveguides near the lower ends of their usable range (in fundamental *mode)* was fully developed before the end of WWII, spearheaded by Herculean efforts at the MIT Radiation Laboratory [1–3]; but as we shall see, this too becomes severely inadequate in the millimeter-wave (mmw) or “Terahertz” regime (generally meaning 0.2–3 THz, or wave lengths in the range 1.5–0.1 mm).

The size of fundamental*-mode* waveguides decreases linearly with the wavelength, so this method of energy transfer also becomes too lossy for many purposes above ~100 GHz. The solution is to *not* reduce the waveguide size as the frequency increases and instead figure out how best to deal with the implications – that the waveguide may then behave as a resonator with tens of thousands of *modes,* leading to very high attenuation at many frequencies of interest and possibly with no region of flat transmission response.

The italics on “*modes*” in the above are to draw attention to the confusing usage of this term for at least three distinctly different meanings in this context: (1) transverse field profile of a propagating wave, (2) frequency of a resonance, or (3) field distribution of a resonance. For the first portion of this paper, we will be using the first definition exclusively, and will signal when we shift to one of the other definitions. We will forgo more precise mathematical descriptions and try to clarify with reference to some more familiar transmission cases.

For a given hollow waveguide size, there is a minimum (cut-off) frequency at which any mode (field profile) can propagate, but there is not an upper limit. That may seem counterintuitive, but the field contours (as seen in the next section for circular waveguides) show the shape of the electromagnetic (EM) field as that mode travels down the waveguide. It is only the wavelength in the propagation direction that is inverse with frequency and limited by relativity – Einstein didn’t affect field extents transverse to propagation, as energy and information travel only in the propagation direction, the z axis by convention. Maxwell’s equations, with the mathematics of Bernoulli and Bessel, determine which propagating field profiles are allowed in the transverse directions in waveguides.

While wave propagation even far above cut-off was also covered by the classical treatments for perfect waveguides of simple geometry (circular or rectangular) [3,4], the real world is not perfect; and then it gets much more complicated. The understanding of energy transfer in this “*overmoded”* regime is far from complete, both from theoretical and experimental perspectives. The purpose of this paper is to add some insights to this rapidly developing area in a way that can be of practical value, particularly to newcomers.

Transmission of mmw in a waveguide occurs via TE (Transverse Electric field waves), TM (Transverse Magnetic field waves), and possibly TEM modes (Electric and Magnetic fields are perpendicular to the direction of propagation). In the TE case there is no electric field parallel to the direction of the traveling wave (but there is magnetic field in that direction). In the case of TM there is no magnetic field parallel to the direction of travel (but there is electric).

Efficient transmission of electromagnetic power below 3 GHz is quite straightforward using coaxial cables. Small coaxial cables can be used even beyond 100 GHz, but with very high losses. They normally operate in the TEM mode, where the electric and magnetic fields are everywhere transverse to the propagation direction, surface current directions are everywhere collinear with the propagation direction, and there are negligible E and M field components (M usually quantified in terms of the H field rather than the B field) in the propagation direction. The use of subscripts in the TE and/or TM notation can be confusing. Without going through a rigorous discussion, the first subscript denotes the number of full wave patterns around the circumference. Whereas, the second subscript indicates the number half wave patterns across the diameter.

At the microwave frequencies traditionally seen for low-field Electron Paramagnetic Resonance (EPR) [4], rectangular brass waveguides have normally been used, as their losses are much lower than coax in the 2–70 GHz range, though their relative advantage decreases with increasing frequency. For example, the attenuation in rectangular waveguide WR-15 (3.8 mm x 1.9 mm) at 65 GHz is ~2.9 dB/m [5], compared to ~6.2 dB/m in semi-rigid coax type RG-405 (which has 1.68-mm PTFE dielectric outside diameter, OD). The attenuation in coax cables is simply proportional to the square root of frequency *f* from a few MHz to their upper limit, which is roughly when their circumference is comparable to the wavelength (and other modes begin to propagate) [4]. The attenuation in fundamental-mode waveguides, on the other hand, at their normal frequency of use is roughly proportional to *f*^3/2^ – about 1.2 dB/m in WR-28 at 30 GHz, compared to about 26 dB/m in WR-3 at 250 GHz [5].

**Fig. 1.**
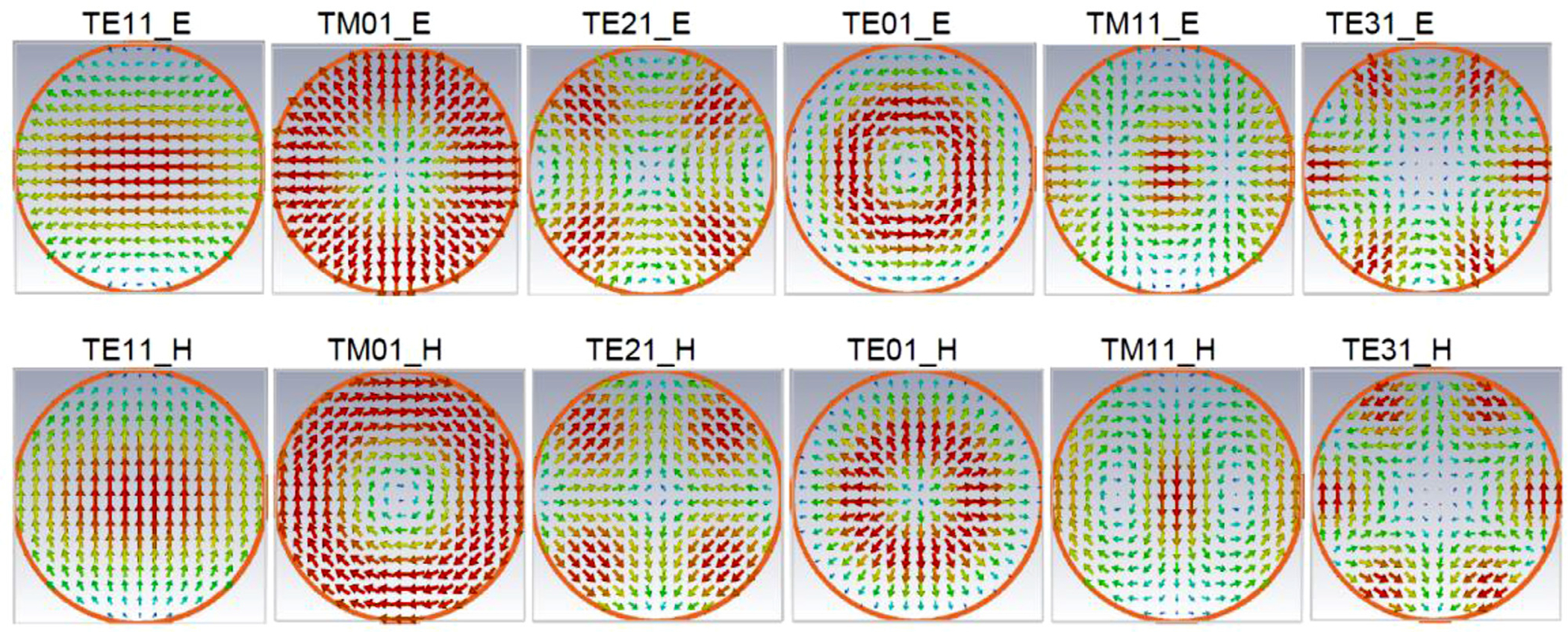
The E and H fields of the first six modes of the circular waveguide are illustrated here, with field intensity shown in a color scale, where red is highest.

There is a wide range over which a rectangular waveguide (WG) can be used with little effect from severe bends (in either direction) on attenuation or reflection, or concern about mode conversion, because the cut-off frequency of its second mode (TE_20_) is about twice the frequency of the (fundamental) TE_10_ mode [4, 6, 7]. Above ~70 GHz, round waveguides are more often used to simplify manufacturing, even though the propagating modes are more closely spaced. As with rectangular waveguides, they have usually been used in their lowest mode, which for round waveguide is the TE_11_ mode – one azimuthal phase cycle and one radial phase cycle of the E field, with the E field everywhere transverse, as illustrated in Fig. 1.

Round waveguides too can often be bent as needed with little effect on attenuation or reflection if operated below the cutoff of their second mode, the TM_01_ (which has no azimuthal phase change and one radial phase cycle of the M field, with M everywhere transverse), which is ~30% above cut-off of the TE_11_.

There is, however, an additional issue with circular waveguides that often gets overlooked. There is not a single TE_11_ mode. Rather, there are two orthogonal (degenerate) TE_11_ modes, and the same is true of many of the higher modes. It is very easy for minor imperfections and small deviations from perfect circularity to cause substantial coupling to the orthogonal (cross-polar) TE_11_ mode, which might be mostly reflected at the load end of the waveguide, depending on the nature of the load. Regularly spaced cross-pins in the waveguide can eliminate this problem [8], and we will present some novel ways to minimize coupling to the cross-polar (CP) modes in overmoded circular waveguides.

When near cut-off of the second mode in circular waveguides in the THz regime, attenuation of fundamental mode is usually deemed unacceptable, so somewhat larger sizes are normally used. For example, calculated TE_11_ attenuation (assuming a high-quality gold surface) for an inside diameter (ID) of 1.5 mm (a commonly used size for 140–210 GHz) is ~14 dB/m at 150 GHz, ~11 dB/m at 180 GHz, and ~10 dB/m at 210 GHz. For this size waveguide, cut-off for the second mode is 150 GHz, so in addition to coupling to the cross-polar mode (if not suppressed), larger imperfections could lead to some coupling to the TM_01_ mode.

The best options for transmission of microwave power in the range of interest to high-field DNP (200–600 GHz) without excessive losses over distances of more than ~5 cm are usually either using open beams or overmoded waveguides – waveguides operating far above the cut-off frequency of the lowest-loss mode and thus capable of supporting many modes. If open beams are used – as commonly seen in laser beams, from IR to UV – the divergence is limited by the size and precision of the launching optics. With perfect optics, the diffraction-limited beam divergence (full) angle *θ* is

(1) $$\theta =\lambda /\pi w_{0}$$

where *λ* is the wavelength and *w*_*0*_ is the minimum “beam waist” radius in the source [9]. For example, *θ*=95 mrad (5.44°) for an optically perfect 200 GHz (*λ*=1.5 mm) Gaussian beam from a source beam diameter of 10 mm. Hence, this beam diameter would increase to ~200 mm (at 1/e^2^, or 0.135 intensity) over a distance of 2 m. The best available horns with lens can have 3 dB beam full width divergence as small as 0.5°, but this requires a horn diameter greater than 10 cm (and probably a length greater than 40 cm) at 200 GHz. In addition, there are mechanical alignment, stability, and vibration issues that can become daunting [10]. Pairs of ellipsoidal refocusing mirrors in combination with flat mirrors, all oriented at 45° with respect to the beam, can recover most of the above divergence. This permits quasi-optical tables to be used very effectively for control of beam polarization, modulation, and splitting with practical mirror and table dimensions for typical gyrotron beam waists (~15*λ*) [11–13]. While open beams have been used successfully within some DNP probes [11], they do not appear to be the best option in most cases.

The benefit of overmoded waveguides (OMWGs) is that they can transmit certain lower modes with very low loss. Most (though certainly not all) treatments of OMWGs in the terahertz regime have focused on essentially fixed frequencies rather than broadband behavior (bandwidth 20–50% of center frequency) in relatively large corrugated waveguides [14–17]. Corrugated waveguides have been shown to offer substantial benefit in loss reduction, flatness of response, and tolerance of imperfections at diameters greater than ~6*λ*; but there are many places (particularly in complex MAS-DNP-NMR probes for use in narrow-bore (NB) magnets) where space constraints require the use of much smaller waveguides. Moreover, it is often desirable to taper the beam down to a much smaller diameter to increase its intensity in small samples. For example, one case reported using a corrugated downtaper for a 1-kW 94-GHz beam from a 50-mm OMWG to 3-mm ID to obtain H field (A/m) intensity in a glycerol/D_2_ O/H_2_ O/trityl sample at 80 K in a 3-mm ENDOR-type cavity [18] greater than 2 kA/m [19], which is greater than that in a uniform 50-mm beam in free space by a factor > 40 (and recall that power intensity is quadratic in E and H). Others have used tapered Teflon rods – in some cases ending as a convex lens – to concentrate the beam emanating from a corrugated OMWG into small samples [20, 21]. Bruker recently substantially improved microwave utilization efficiency in their 1.3-mm MAS-DNP probe by using a teflon lens to reduce the beam emanating from their 7.6-mm corrugated OMMG to under 2 mm before feeding it into a smooth OMWG of 2-mm diameter and ~10 mm length just prior to reaching the rotor [22], However, in complex multi-channel MAS-DNP probes, it can be beneficial to reduce the waveguide down to something close to fundamental-mode circular waveguide well before the sample region to permit fitting it around other essential rf and rotor-handling hardware.

The primary impetus for the work reported in this paper is to develop efficient overmoded broadband downtaper transitions that would be compatible with the severe space constraints in high-field NB magnets and would be readily manufacturable, as high-precision corrugated metallic downtapers for the sub-mmw regime are very difficult to produce by traditional machining methods. They are usually made by high-precision electroforming or electrical discharge machining (EDM) methods that are rather costly [23]. Additive manufacturing (3D printing) methods have progressed dramatically in recent years, and several groups have successfully made various microwave components by metalizing 3D-printed plastic parts [23–26]. This works well for parts where a few errors of 0.2% plus 25 *μ*m have little effect, and where the thermal contraction of plastic upon cooling to cryogenic temperatures is also acceptable. Our evaluations of several more demanding 200-GHz components say they still fall short of the precision needed for many corrugated THz components. Straight THz corrugated OMWGs can be made in very short sections by conventional methods from readily machinable Cu-Zn-Ni-Pb alloys, and they can be made in somewhat longer sections by EDM methods. These sections can then be joined by gluing or soldering, but with some unavoidable errors at each junction. Much longer sections can be made by the stacked-ring method, in which thin precision metal rings of alternating ID’s are stacked inside a pipe of precision ID [27–29]. All of these methods become quite costly for OMWGs above 250 GHz, especially with non-constant ID.

We have simulated numerous alternatives to corrugated circular OMWGs, including most of the previously reported alternatives (except for many of the low-power fiber-optics options, which we will later briefly mention) and several novel designs. We conclude that corrugated circular metallic OMWGs are the best of the reported options to date (except from a cost perspective) for diameters down to ~1.5*λ*, but the corrugation parameters for small OMWGs need to be significantly different from the previously published guidelines that have worked well for large OMWGs. With numerically optimized small OMWGs, easily manufacturable smooth downtapers appear to work as well as corrugated downtapers in many cases relevant to MAS-DNP probes. Our example simulations will be mostly for the 170–230 GHz range, but the lessons and results should be readily applicable to other ranges by simple scaling.

## 2. The basics of circular waveguides

The transverse profiles (and relative magnitudes, by color) of the first six distinct modes at the crests of the waves in a circular waveguide were seen in Fig. 1. The propagation of the TM_mn_ mode is characterized by cut-off constant *χ*_mn_ that is the nth zero of the Bessel function of the first kind of order m. For the TE_mn_ modes, the appropriate *χ* comes from the derivative of the respective Bessel function [1–4, 6–7,30,31]. The cutoff frequency *f*_c_ in either case is given simply by

(2) $$f_{c}=c\chi /2\pi a,$$

where *c* = (*με*)^−0.5^ for permittivity *ε* and permeability *μ*, *a* is the tube radius, and the appropriate *χ* is used.

The guide phase velocity *v*_p_ (which is always greater than *c*) for a propagating mode is given by

(3) $$v_{p}=c/{\left[1-{\left(f_{c}/f_{0}\right)}^{2}\right]}^{0.5},$$

where *f*_0_ is the excitation frequency. Note that the phase velocity goes to infinity at the cut-off frequency and becomes imaginary at lower frequencies, meaning the mode doesn’t propagate. A wave fed into a waveguide below the lowest *f*_c_ of the waveguide is reflected, with some attenuation and phase shift.

While the phase velocity is conveniently defined by Eq. (3), it can be more intuitively understood from the “guide” (actual) wavelength *λ*_g_ within the waveguide, which is related to *v*_p_ by the standard relationship,

(4) $$\lambda_{g}=v_{p}/f_{0}$$

The phase propagation constant *β* is defined as 2*π* / *λ*_g_ [rad/m]. The product of the group velocity (velocity at which power or information can be transmitted) and phase velocity is always *c*^*2*^. Hence, the group velocity *v*_g_ is always less than *c*, consistent with relativity.

(5) $$v_{g}=c{\left[1-{\left(f_{c}/f_{0}\right)}^{2}\right]}^{0.5},$$

Note that waveguides are dispersive – the velocities are functions of frequency. That is different from standard TEM propagation in coax lines, where *f*_c_ is zero and *v*_g_ = *v*_p_, both of which scale simply with *ε*^−0.5^.

In the views shown in Fig. 1, both E and M appear to be transverse to the propagation direction. The distinction between TE and TM is that in the TE modes the E field remains transverse (with the profile shown) as the wave propagates, while the M field changes between transverse and axial (and its magnitude profile changes) every *λ*_g_/4. For the TM modes, the M field remains transverse as the wave propagates while the E field changes between transverse and axial every *λ*_g_/4.

The classical expression for rf surface resistance *R*_*s*_ [Ω] of a conductor is

(6) $$R_{s}=1/\sigma \delta$$

where *σ* is the DC conductivity [S/m] and *δ* is the classical skin depth [m].

(7) $$\delta ={\left[2/(\omega \mu \sigma )\right]}^{0.5}$$

However, from the combination of surface roughness, surface impurities, plating problems, and anomalous skin effects, Eq. (6) is usually a significant underestimate of *R*_*s*_ at frequencies above ~200 MHz (where *δ* ~5 *μ*m in pure copper). Surface roughness in drawn copper tubing is likely to be ~0.3 *μ*m rms, which is comparable to the classical *δ* in pure Cu at 50 GHz. From our experience, we believe correction factors for *R*_S_ of “high quality” room temperature (RT) copper surfaces should be above that of most reported experiments, which have been ~1.1 at 14 GHz and ~2 at 670 GHz [5, 32, 33]. We measured attenuation of the TE_11_ mode in commercial copper tubing (alloy C122, 99.9% Cu, 0.06% P, 85% DC conductivity of pure copper) of 1.6-mm ID (lengths in the range of 16–205 *λ*_g_) to be 7.8 dB/m at 200 GHz, which implies a surface resistance correction factor of ~2.3 assuming there was negligible conversion to the TM_01_ mode. (The transmitted power was measured at the angle giving maximum output to largely include effects of any conversions to the cross-polar TE_11_ mode.)

The characteristic impedance *Z* of a waveguide for a TE mode is

(8) $$Z=\eta /{\left[1-{\left(f_{c}/f_{0}\right)}^{2}\right]}^{0.5},$$

where *η* = (*μ*/*ε*)^0.5^ (376.7 Ω for vacuum).

Expressions for attenuation *α* of the classical modes are also well known, but they are a bit more complicated and need not be repeated here. The interested reader may refer to the references [1–4,6,7]. The classical equations are valid from just above cutoff to very highly overmoded. Several excellent text books and courses are freely available online [6, 7].

**Fig. 2.**
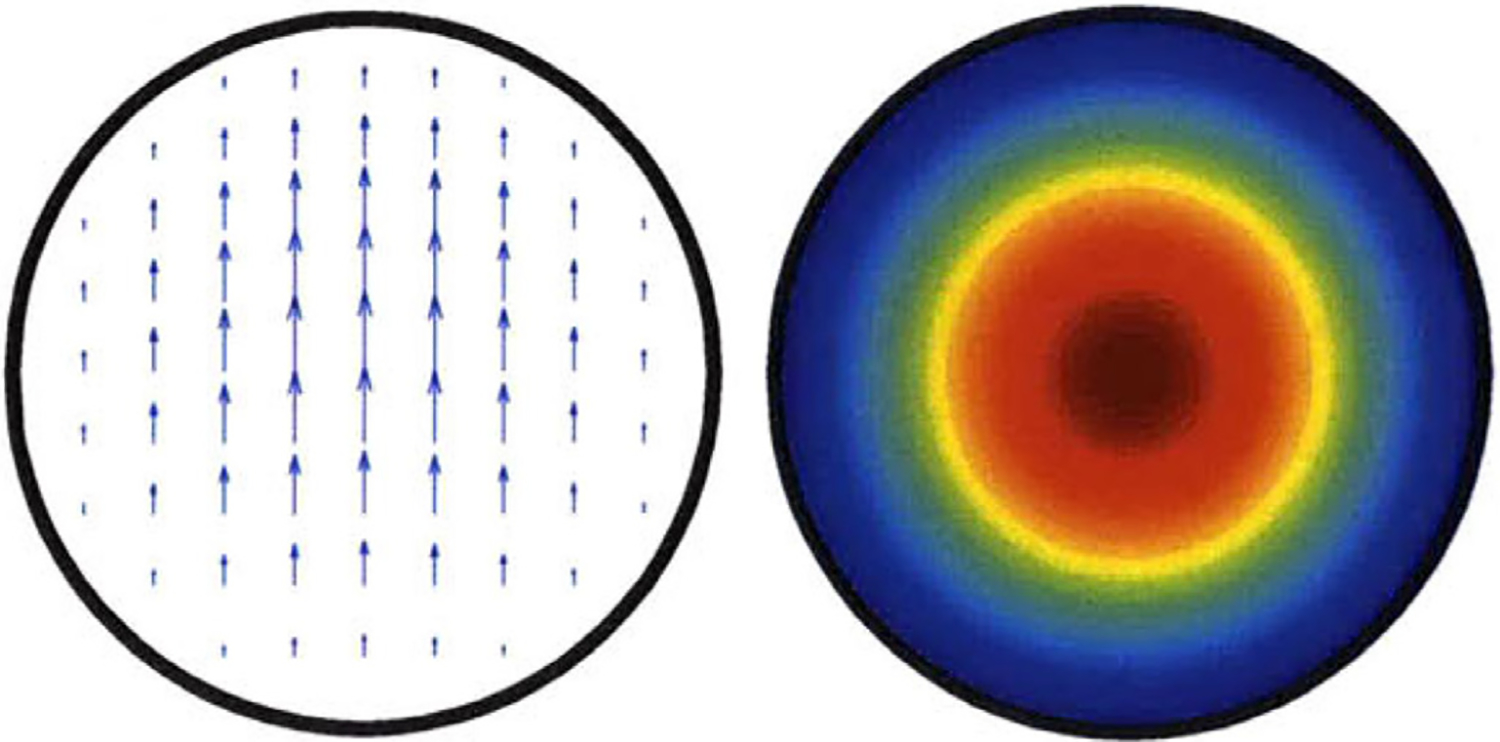
Field vectors (left) for the HE_11_ E field in a highly overmoded circular waveguide, and power density (right), indicating very low power density at the edges. With an optimum profile, the fields are 1/e at 0.32*a*.

Hybrid modes (with profiles similar to the sum of a TE and TM mode) propagate in corrugated and in highly overmoded smooth waveguides. In the fundamental HE_11_ (sometimes called linear LP_01_) mode the field essentially vanishes at the boundary, and the E field is linear [14–17], as illustrated in Fig. 2.

The HE_11_ mode is a unique quasi-Gaussian mode in corrugated waveguides with a propagation constant equal to that of the TM_01_ mode in a smooth waveguide of the same ID. However, it can be well approximated by a weighted sum of the TE_11_ and TM_11_ modes. In our simulations the input port is excited with 0.95*TE_11_ + 0.31*TM_11_ amplitudes (or 90% TE_11_ and 10% TM_11_ powers), each at phase 0°, which we found to be a good approximation of an HE_11_ beam (in radial profiles of E and power flow) using just two modes. (Others have reported amplitude factors of 0.92 and 0.39 at different phases [30]. We did see a slight improvement in the HE_11_ approximation by subtracting some TM_41_, but we determined it was not significant and did not include that in the simulations reported here.) In a highly overmoded waveguide the HE_11_ beam is similar to a typical TEM_00_ laser beam, which heuristically explains why loss is surprisingly small from relatively long gaps [15,17]. More precisely, the HE_11_ mode couples very well (98% transmission) to the free-space Gaussian TEM_00_ mode. When fed into smooth circular waveguide, it converts mostly to TE_11_ and TM_11_.

Table 1 lists some characteristics at 200 GHz for the first 12 distinct (non-degenerate) modes in smooth circular waveguides, and the first three HE modes in corrugated waveguide [14]. The values here are for *a* = 3 mm, and (except for the HE_11_ mode) a surface resistance *R*_S_ of 0.3 Ω, appropriate for a typical gold-plated surface with plating thickness more than 4*δ* at 200 GHz. (Commercial components always have nickel (which has high *μ*) under the gold, and the gold thickness is often too thin, which probably explains much of their excessive loss relative to theoretical expectations.) Note that lowest attenuation here is seen for TE_01_. While this mode has been used effectively in very high-power applications (waveguide diameters greater than 27 mm) up to 70 GHz [30, 34–36], efficient coupling to and from this mode would be very difficult to implement in small waveguides in the THz regime.

The attenuation shown in Table 1 for the HE_11_ mode is from the expressions in [16] for trapezoidal corrugations (depth ~*λ*_0_/3, and pitch of *λ*_0_/3 in the propagation direction, where *λ*_0_ is the free-space wavelength) and with a typical brass surface (*R*_S_ of 0.45 Ω at 200 GHz). The listed value is for a 6-mm ID, as for the other modes. When there is a hard OD constraint, the comparison is very different. The smooth 6-mm waveguides could easily have an OD of 6.6 mm, but it would be difficult to manufacture the corrugated waveguide of similar OD with ID greater than ~4.5 mm. The referenced expression for attenuation of HE_11_ at 200 GHz in a corrugated OMWG of 4.5 mm ID gives 2.5 dB/m or ~2.3 times that for TE_11_ in a smooth OMWG of the same OD (though as we report later, our simulations indicate the referenced expression overestimates loss in small corrugated OMWGs and underestimates loss in large corrugated OMWGs). The benefit of the HE_11_ mode increases rapidly with waveguide size and frequency, as its attenuation decreases as the cube of the diameter in corrugated waveguides while that of the TE_11_ mode in smooth waveguides decreases only linearly with increasing diameter.

There are a number of loss sources such as miter bends, polarizers, waveguide flange gaps, misalignments, waveguide sagging, and corrugation inaccuracy in overmoded corrugated waveguides. However, a number of excellent works showed that these losses (including mode conversions) should in principle be limited to no more than a few percent from each loss-source for HE_11_ [14–17,37]. One of the more significant contributors to mode conversions seen in one study was input-beam tilt [15], where an error under 0.2° seemed needed to keep mode conversion below 1% when feeding a Gaussian beam into a corrugated waveguide. Another study calculated loss in large miter bends (63.5 mm) to be 0.025 dB (0.6%) for *a*/*λ*=10 and 0.12 dB (3%) for *a*/*λ*=4 [17]. However, even a dozen sources of 1–3% loss each (and mode conversion is not necessarily a loss) doesn’t add up to the 50–70% total loss often anecdotally reported by DNP researchers. Apparently, the loss in small THz miter bends is more likely to be in the 4–8% range because of greater relative manufacturing errors. These referenced loss studies did not investigate tapered transitions, perhaps because such were not needed in their primary application – plasma heating of the ITER reactor, where a large corrugated waveguide of constant size (60–90 mm ID) could be used from the beginning to the end of the waveguide run. There is indeed considerable literature on tapered overmoded transitions [30,34], including a nice recent addition [38]; but the prior literature on such has generally been focused on cases where the number of propagating modes may be perhaps a dozen, not hundreds, and the frequencies are much lower.

For those readers unfamiliar with S-parameters it is worth while to briefly mention them. The scattering matrix (S-matrix) is a mathematical construct that quantifies how rf power propagates through a multi-port network. S-parameters are usually displayed in a matrix format, with the number of rows and columns equal to the number of ports. For the S-parameter S_ij_ the j subscript stands for the port that is excited (the input port), and the “i” subscript is for the output port. Thus, S_11_ refers to the ratio of the amplitude of the signal that reflects from port one to the amplitude of the signal incident on port one. Parameters along the diagonal of the S-matrix are referred to as reflection coefficients because they only refer to what happens at a single port, while off-diagonal S-parameters are referred to as transmission coefficients, because they refer to what happens at one port when it is excited by a signal incident at another port.

In highly overmoded microwave systems, the whole is not the sum of the parts from a conventional circuits perspective. When only a single mode can propagate in ports, the system can be broken up into parts, each with single-valued S-parameters for its input and output ports, and conventional rules apply – make S11 and S22 small for each separate component (with matched port impedances), and the components can be connected together with a predictable total transfer function. That approach is not adequate for overmoded components because each component has dozens (maybe hundreds) of S11’s, S22’s, S12’s, and S21’s – one for each combination of significant input and output modes. Moreover, the details of the imperfections in the connections between the components – even at the level of a few microns for the THz regime – can have a significant effect on results. The primary driver in our OMWG research has been to obtain a better understanding of what is likely to work best in a world of imperfect components, severe external space constraints, and manufacturing cost constraints.

We have simulated numerous widely differing types of RF problems, from simple to extremely complex, in frequency-domain (FD) using COMSOL-RF [39] over the past eight years, from which we have gained many insights. Moreover, we always found those simulation results to agree very closely with experimental results – until we began working with models that included highly overmoded ports. We eventually concluded that COMSOL’s method of dealing with overmoded ports may be the reason we were not able to get any of our attempts at COMSOL simulations of corrugated OMWGs to come even close to well established experimental results. We should hasten to say our (admittedly limited) experience with CST’s FD solver was even more disappointing in all respects (robustness, speed, mesh controls, and accuracy) but we are very “bullish” on CST’s time-domain (TD) solver [40]. A drawback of FD is that one needs to know in advance where all the modes will appear (and there may be scores of unpredictable modes, as will be seen) and instruct the solver to perform calculations at 8 or more specific frequencies very near each mode – an arduous task. In contrast, a single TD run calculates response over as broad a range as desired, typically with spectral resolution of 0.1%, or 0.02% with a little more patience. As a result, all of the simulation results presented here are the result from CST-TD calculations. We, like hundreds of other users, have experimentally validated the CST TD solver time and time again. Prior to looking at very highly OMWGs we had not seen any results from it – with sufficiently fine meshes – that were clearly at odds with experimental results. These validations, for example, included confirming that CST gives attenuation results for various modes in smooth waveguides of various sizes that agree with the classical equations. We report here some experimental results that agree with the CST TD simulations for high-mode problems of moderate size (~20 M mesh cells). We also report simulation results for some high-mode problems (up to ~320 M mesh cells) that are at odds with expectations from some published materials on OMWGs.

We would note that validity of the results presented in the following was checked by confirming that they did not change significantly as the mesh (which was generally finer than default) was made yet finer (more mesh cells), time step made smaller than default (typically ~0.1 ps), or energy decay taken further. All of the results presented went to −30 dB energy decay (except if noted otherwise), though often there was little difference – other than a smoother response – when going only to −18 dB decay.

## 3. Smooth THz waveguides with uptapers and downtapers

A solid-state source will often begin from a microstrip circuit, then usually go into fundamental-mode rectangular waveguide (possibly via a short intermediate coax section), and then to fundamental-mode (TE_11_) circular. The benefit of then expanding (in what is generally called an uptaper) to OMWG for distance transmission (even if fundamental-mode is needed at the end) has long been appreciated [30,33,36]. Unfortunately, trapped modes (essentially cylindrical cavity resonances) between the uptapers at the beginning and the downtapers at the end are then likely to cause problems. The severity of these resonances increases with the radial extent of the final downtaper and with surface conductivity, and it decreases (within limits) with taper angle [33].

**Fig. 3.**
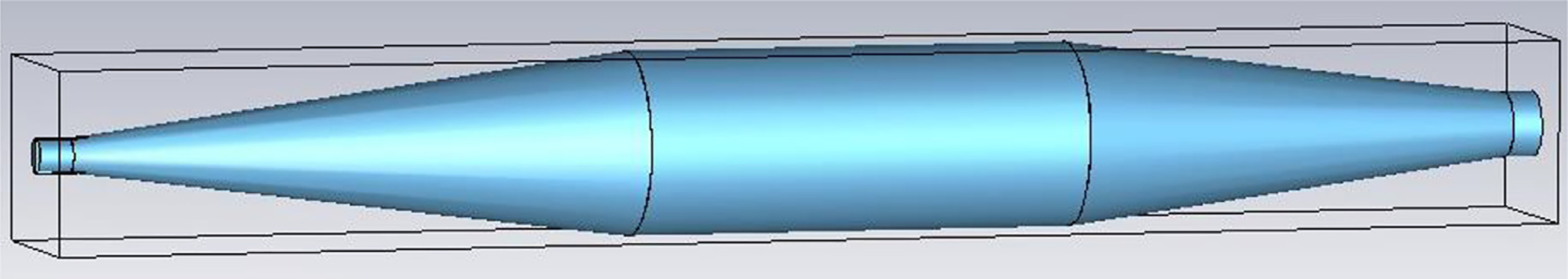
Example-1 geometry: an uptapers from fundamental-mode (on the left above), followed by a short smooth OMWG (8 mm diameter, 20 mm long), and then a downtaper, as described in the text, with no imperfections. The input is on the left, and output is on the right.

**Fig. 4.**
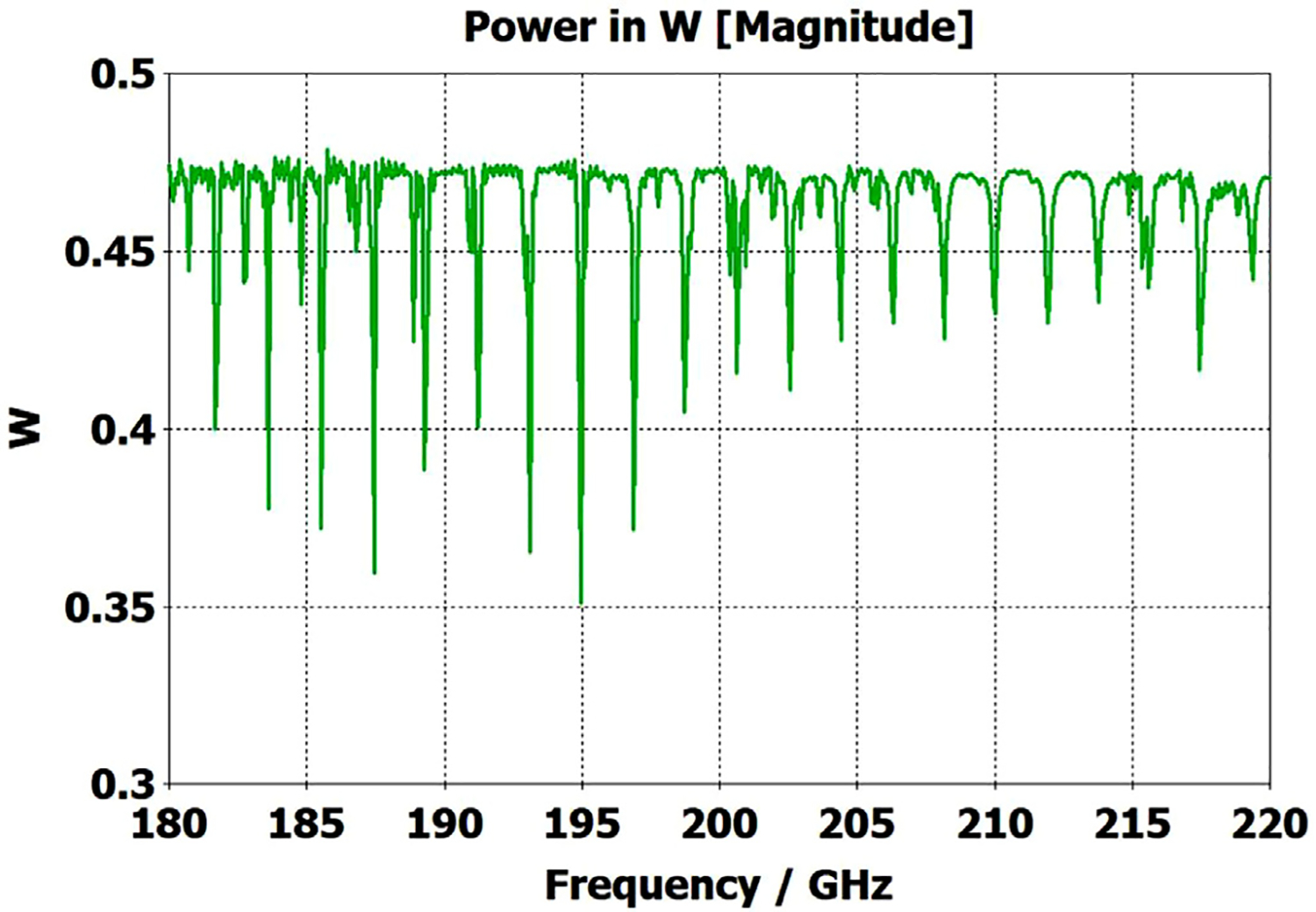
The P_A_ (power accepted) plot shows total power (all modes) accepted at the output port for 0.5-W TE_11_ in-port excitation of the geometry shown in Fig. 3. The output *power* was over 92% TE_11_ except at the absorptive spikes, where it was up to ~25% TM_11_.

A short version of this case is depicted as example-1 in Fig. 3. It begins with in-port radius r_in = 0.75 mm, followed by an uptaper of 7.5° semi-angle to a 20-mm-long smooth OMWG of radius r_omwg = 4.0 mm, following by a downtaper of 7.5° semi-angle to out-port (Port 2) of radius r_out = 1.42 mm, which is large enough for all modes up through TE_31_ to propagate at frequencies above 180 GHz. The central portion of the CST transmission spectrum results for this example, for 0.5 W TE_11_ 160–240 GHz excitation at the input port (port_1), are shown in the P_A_ plot (power accepted) at the output port in Fig. 4. The linear P_A_ plot is generally more useful than S-parameter plots in OMWGs if one is interested in the sum of all modes. At the resonances indicated by the (negative) spikes, the reflected power from port_1 (S11, not shown) also spiked. However, it was still not bad, as only two of the spikes had S11 greater than −16 dB. The P_A_ negative spikes primarily reflect increased dissipation in the walls by these trapped modes, which also convert some of the TE_11_ to TM_11_, which propagates to the out-port. A perfect electric conductor (PEC) on the YZ plane and a perfect magnetic conductor (PMC) on the XZ plane (appropriate for TE_11_, HE_11_, TM_11_, and TE_31_ for input phase of 0°) were used to speed up calculation, which then took only ~7.5 min for this model with 2.2 M mesh cells with a GV100 GPU (7.4 TFlops double precision) and dual Xeon 6148 s.

Manually calculated surface losses (which were mostly in the short 1.5-mm waveguide following the in-port and in the initial portion of the uptaper) using classical expressions for TE_11_ mode were in good agreement with those seen from the simulations (~2.5%) at low-loss frequencies for surface *σ*=1.5E-7 S/m (unpolished gold surfaces, with correction factor for typical roughness). With a slightly larger r_in, similar performance could be achieved with TM_11_ or HE_11_ excitation.

**Fig. 5.**
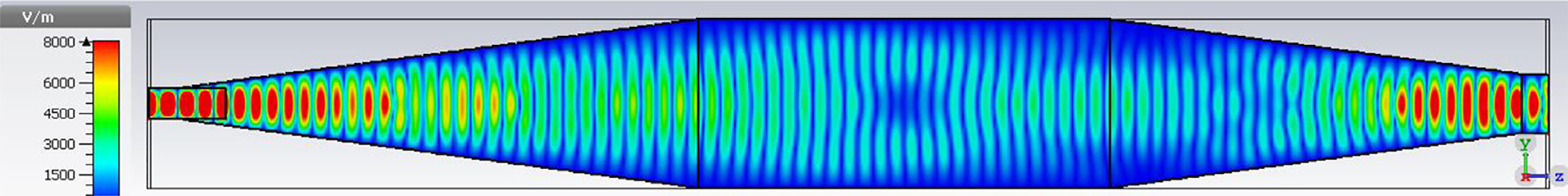
E field magnitude at a “good” frequency”, 200 GHz, from the CST TD simulation of the geometry in Fig. 3. Red is 8000 V/m.

**Fig. 6.**
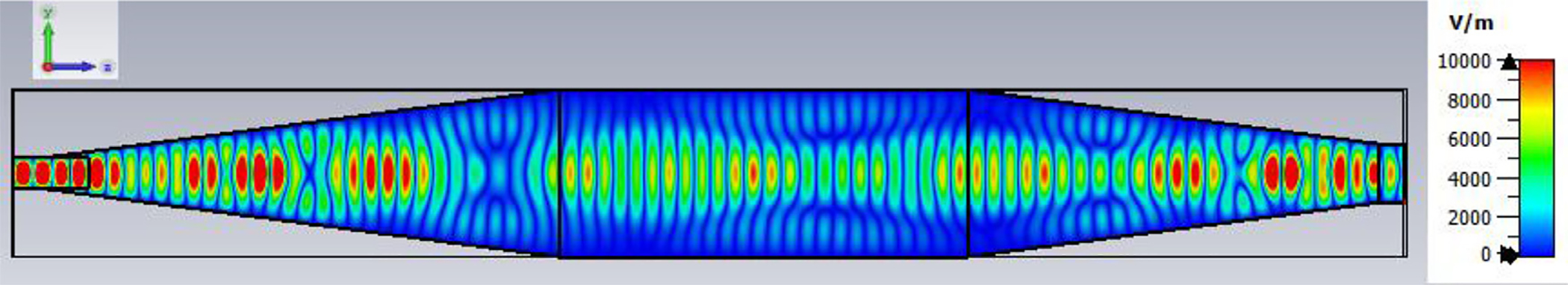
E field magnitude at a spike frequency, 196.8 GHz, for the case in Fig. 3.

A snapshot (at a selected instant, or phase) of the E field magnitude, E_abs_, on the YZ plane from the CST TD results at 200 GHz (which according to Fig. 4 is a “good” frequency, or “good” waveguide length), is seen in Fig. 5. It shows a rather smoothly expanding and converging plane wave with little effect from reflections or trapped modes. More pronounced effects of trapped modes and mode conversions are evident in the plot in Fig. 6 at what Fig. 4 shows to be a spike (“bad”) frequency, but our experience says little can be learned or concluded from such plots, particularly because their appearance changes dramatically with the chosen phase.

**Fig. 7.**
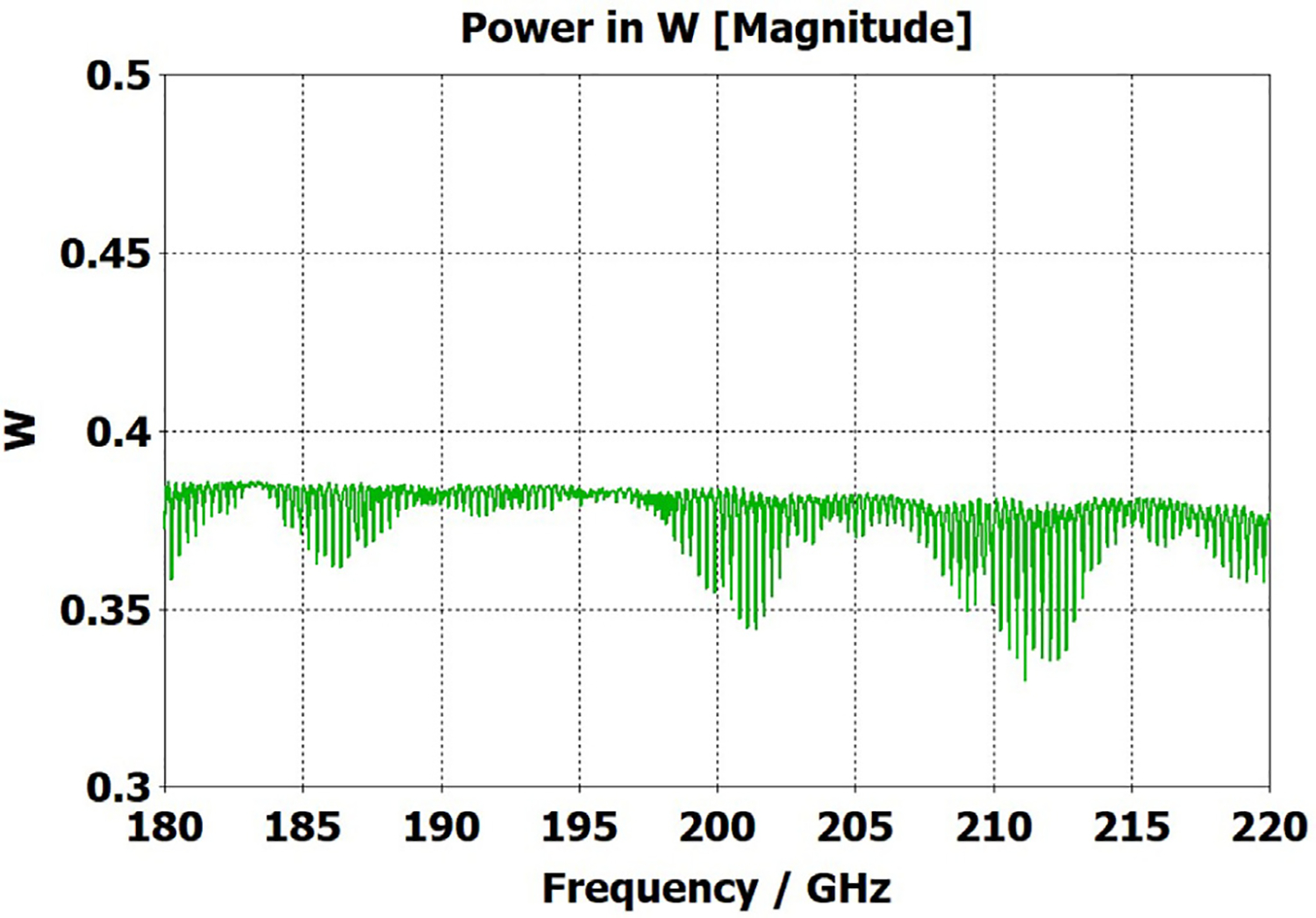
P_A_ for the output port for a geometry as in Fig. 3 except the length of the central 8-mm OMWG is increased by a factor of 20, to 400 mm.

**Fig. 8.**
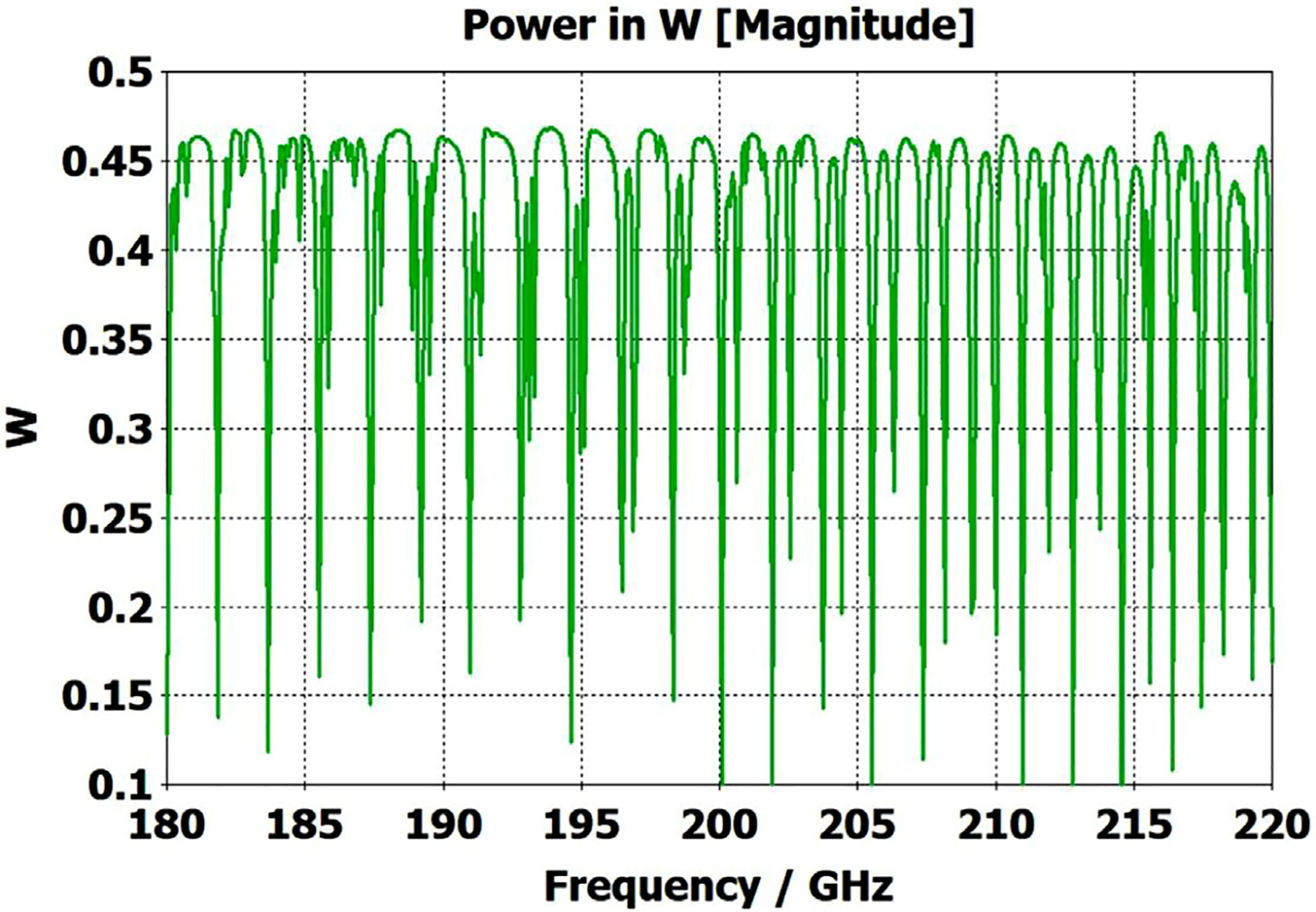
P_A_ for the output port with the short OMWG as in Fig. 3 except the length of the downtaper is increased to bring the final diameter down to 1.5 mm, where only TE11 propagates with the assumed four-fold symmetry.

Quite surprisingly, the transmission spectrum does not become much worse – but actually becomes flatter in places – when the length of the 8-mm OMWG (i.e., the central section with 8-mm ID) is increased by a factor of 20, as seen in Fig. 7. However, when the objective is to taper back all the way down to “fundamental mode” the picture is much worse, as seen for a short case in Fig. 8. Here, the geometry is the same as in Fig. 3 except the downtaper length is increased to bring the final diameter down to 1.5-mm (in which only TE_11_ and TM_01_ propagate below 220 GHz) so that bench experiments might be carried out with standard fundamental mode instruments – to possibly validate the simulations. Here, the output from the simulation is totally TE_11_, as that is the only mode that will propagate below 220 GHz in the output smooth 1.5-mm waveguide with the two symmetry planes imposed (YZ=PEC, XZ=PMC), though when those symmetry constraints are lifted (as in the real world) one sees some TM_01_ and TE_21_ in the output, particularly at the frequencies of the spikes.

Some pertinent questions to try to address now are:

- What can be expected when the geometries deviate from the perfectly smooth and cylindrical surface assumptions of example-1 and include a few small grooves or ridges, as might be expected in practical joints between sections?
- What can be done to minimize the effects of imperfections in joints, cylindricity, and other errors?
- What is the best one can expect to achieve with a long OMWG and likely imperfections if the output must go totally to TE_11_?
- How much will it help if the output can be large enough for modes up to TM_11_ to propagate in the range of interest?
- Does it help or hurt if the downtapering is done in several stages?

What eventually became clear from our experiments and numerical investigations of OMWGs was that even minute surface imperfections at any location where surface currents are high can dramatically worsen the broadband performance. Moreover, the optimization focus must be on broadband flatness with low loss, as it is impractical from a manufacturing perspective to plan to operate between strong absorptive spikes since their locations will be moving substantially with temperature in practical applications, particularly if the waveguide is made of metalized plastic. Less than a 20-K change in temperature of a plastic section 400 mm long changes its length by *λ*/4. That change in length for the case shown in Fig. 7 shifts the mode locations by half their separation, potentially moving the operating point from a peak to a trough.

We should point out here that TD simulations begin with a short broadband (usually Gaussian) excitation pulse (in our cases, about 40 ps, to excite up to 240 GHz) at the in-port, and they follow the progression of the wave emanating from it, in steps of ~0.1 ps in our typical cases. So, if we are simulating a waveguide that is 560 mm long (as in several of the long models presented later), the wave front from the excitation reaches the out-port in ~2 ns, and its reflection gets back to the in-port ~2 ns later. If there is a horn there, as will be the case in many of the simulations presented later, much (maybe most) of the energy in the wave coming back to the horn will be reflected rather than make it to the in-port, where it would be absorbed. That reflected wave then gets back to the out-port ~2 ns later, and so on, as the energy steadily decays. If one terminates the simulation before this primary reflection gets to the out-port, which might correspond to an energy decay of only ~8 dB, the response will appear much smoother, as the resonances have not had time to build up. The mean of that port_2 P_A_ curve will probably be like what comes from a more complete run, but the transmission at any particular frequency can be wildly different. That is not such an issue in the simulations presented in the next section, as they begin from a large port, which doesn’t reflect much of the wave coming back at it. Thus, there are fewer trapped modes, and they are much weaker than in simulations beginning and ending with fundamental-mode waveguides. They are also close to what one would see for the case of excitation by a beam from a quasi-optics bench.

## 4. HE_11_-beam-fed small OMWGs with multiple smooth downtapers and imperfections

The obvious place to begin is with corrugations designed according to previously published guidelines, in which the groove depth *d* is *λ*/4 and the periodicity *p* is *λ*/3 [14–17,41–44], with various groove widths *w*, as the prior recommendations are not clear on optimum relative groove width, *w/p*. The results from numerous simulations of various HE_11_-beam-fed OMWGs, with different extents of downtapers, of various lengths, to various sized OMWGs, led us to the general conclusion that excellent results (low loss, acceptably flat response) could be obtained without corrugating the downtapers if the OMWG size reduction in each downtaper was limited to ~40% and the corrugations in the larger straight sections were numerically optimized. Our simulations of many cases with greater reductions per section, with both smooth and corrugated downtapers, gave more ragged output P_A_ curves, though we note others have reported excellent results from a single large corrugated downtaper [13,19,30], and we did not look at defect-free cases with semi-angles below 3° after seeing little difference over the 3°−7° range.

**Fig. 9.**
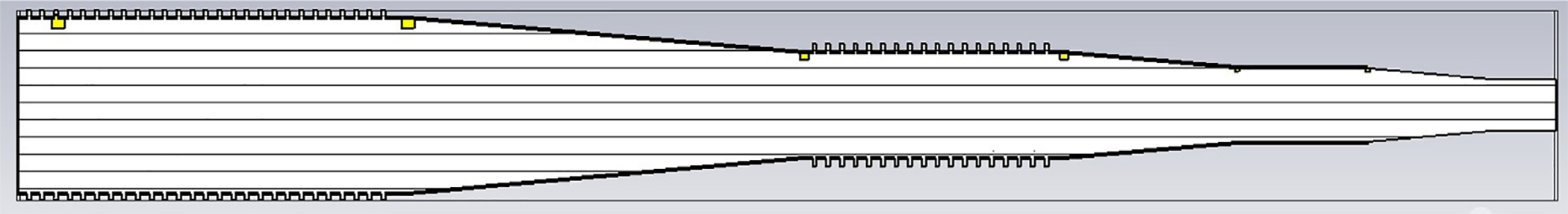
Example-2 geometries, as described in the text, included 6 metallic ring imperfections (shown in yellow and exaggerated in size for clarity), four OMWGs (always longer than depicted here), and three smooth downtapers. For reference, the ID of the first (left side) OMWG is 8 mm, the corrugation groove depths depicted here are *λ*/4 (at 200 GHz) in the first OMWG and 0.32*λ* in the second OMWG, and the corrugation periodicities were each 0.42*λ*.

**Fig. 10.**
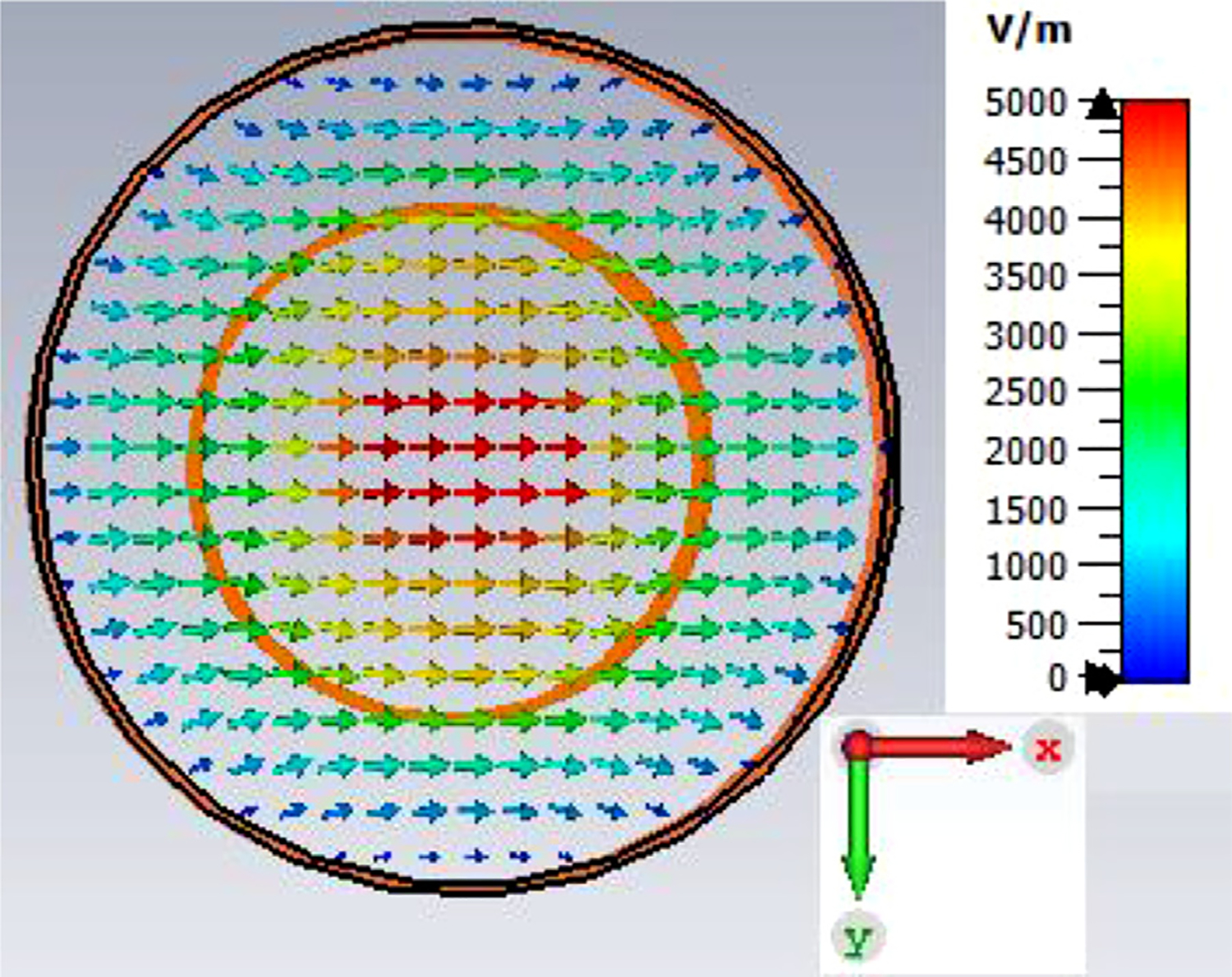
Field vectors of the excitation beam at the input port. The central maximum is 6700 V/m, the next to outermost light blue arrows are ~1000 V/m.

The general features of the waveguides for which data are presented in subsequent figures and discussions are illustrated in cross-section in the shortened version depicted in Fig. 9, here of total length only 48*λ* for better visualization of the small features. The models in this section all begin at 8-mm ID and have three smooth downtapers with 5° semiangles. They are of total length in the range of 130–380 *λ* and included small imperfections in the form of off-center metallic rings of square cross-section, with radial offset equal to their width and with inner diameter the same as that of the waveguide, protruding into the OMWGs near the ends of each OMWG. The waveguides end with a short section (2–3 mm long) having ID in the range 1.5–3 mm. The excitation at input port_1 approximates HE_11_, as confirmed by the E vector plot in Fig. 10, where the E field at 64% of the radius (indicated by the inner orange circle in Fig. 10) was seen to be ~40% of the central peak value, which is close to the 38% value reported to be optimum for excitation by a quasi-Gaussian beam.

In the subsequent mid-length models – unless noted otherwise – the lengths of the first, second, third, and fourth OMWGs are 78 mm, 75 mm, 22.5 mm, and 3 mm respectively, with respective IDs of 8 mm, 4.8 mm, 3.4 mm, and 2.4 mm. As in Fig. 9, the first two OMWGs are corrugated, the rest of the waveguide is smooth, and the output port diameter is 2.4 mm.

**Fig. 11.**
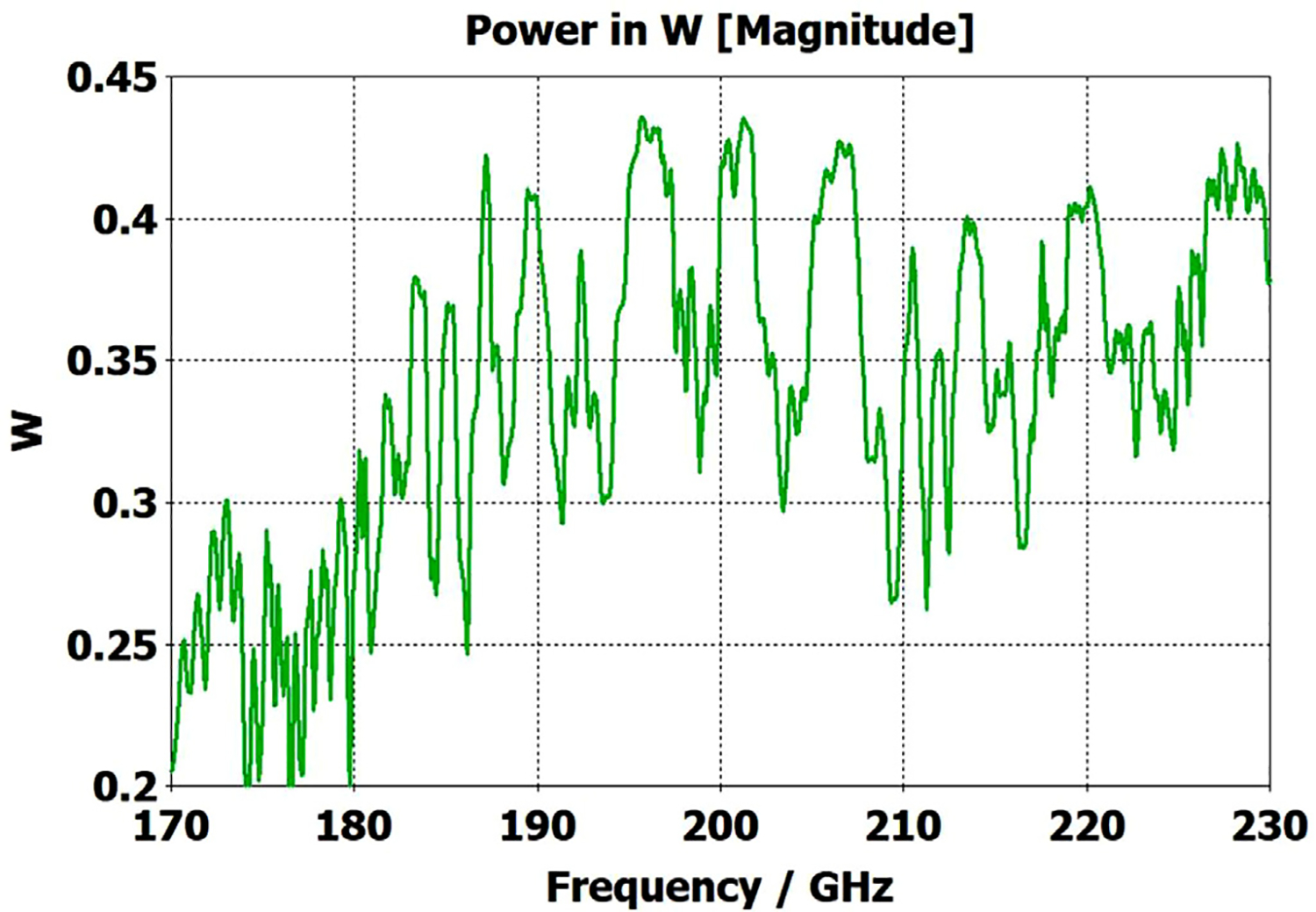
P_A_ for the output port for a mid-length version of a 7-section OMWG similar to Fig. 9 with corrugations according to previous recommendations (*d* = 0.25*λ*, *p* = 0.33*λ*, *w/p* = 0.6) and small manufacturing errors.

Fig. 11 shows the power transmission spectrum for the case with corrugations according to previous recommendations, where groove depths (in the first and second OMWG) were *λ*/4, with *λ*/3 periodicities and 0.6 relative groove widths. The widths of the three pairs of asymmetric rings were 0.05 mm, 0.04 mm, and 0.03 mm, as proxies for small manufacturing errors. Surface conductivities were all 1.5E7 S/m. Corrugating the third (3.4-mm) OMWG was not helpful. We note that the mesh for the case shown in Fig. 11 had ~32 M cells and 0.045-mm minimum cell size. The P_A_ curve was quite different (was more ragged and showed more loss, particularly away from the center frequency) for a mesh with ~20 M cells, though it was rather similar for a mesh with ~13 M cells and 0.05-mm minimum mesh cell size. Similar mesh studies were performed on all cases to insure the meshes were refined sufficiently for valid results. It was also important to utilize enough port modes, and we found using the first 30 modes to be sufficient for a 4-mm port radius.

**Fig. 12.**
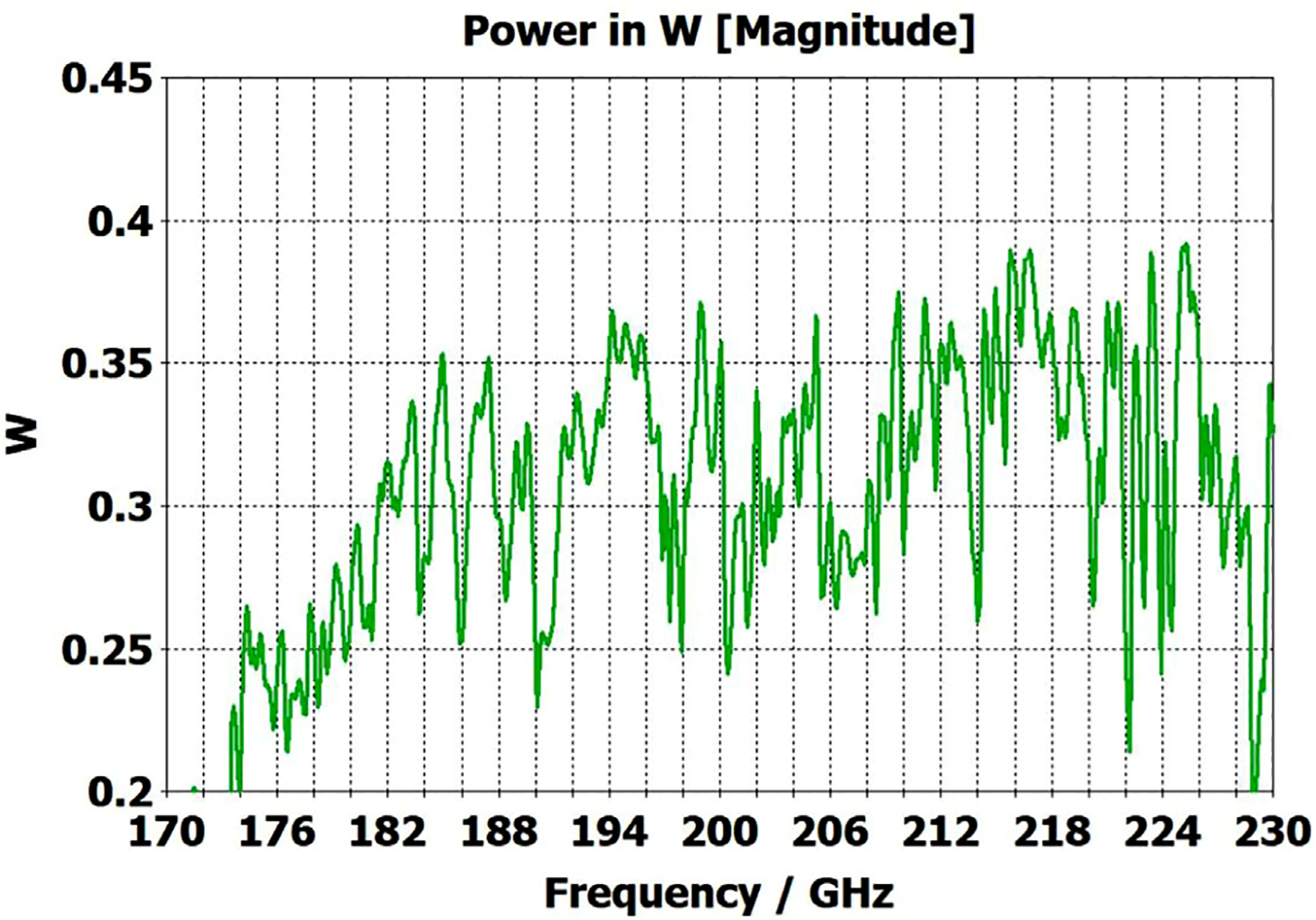
P_A_ for the output port for the same mid-length model as for Fig. 11 except that the sizes of the metallic ring defects were made 3–6 times larger, the relative groove widths were reduced to 0.3, and groove conductivity was reduced by a factor of three.

The widths of the three pairs of asymmetric rings were then increased to 0.3 mm, 0.2 mm, and 0.1 mm as a proxy respectively for the effects of the many small joint errors that are possible from available manufacturing methods. This made the transmission spectrum much worse. One parameter, relative groove width, was adjusted to improve transmission to the extent possible with just that variable adjustment; and the results with that limited optimization, which gave a preferred relative groove width of 0.3, are shown in Fig. 12. This optimization helps regardless of the size of the manufacturing defects and acts as a safeguard against this type of defect. In addition, the conductivity in the corrugation grooves was reduced by a factor of three to 5E6 S/m (as more appropriate for some commonly used nickel-bearing alloys). The primary effect of this conductivity change was to reduce peak-to-peak amplitude of the ripples, and this value was used in all subsequent simulations.

Parameter sweeps were then run to optimize transmission for cases with these *large* defects by changing groove depths, periodicity, relative groove widths, taper angles, and waveguide diameters – the latter with adjustments only over narrow ranges.

**Fig. 13.**
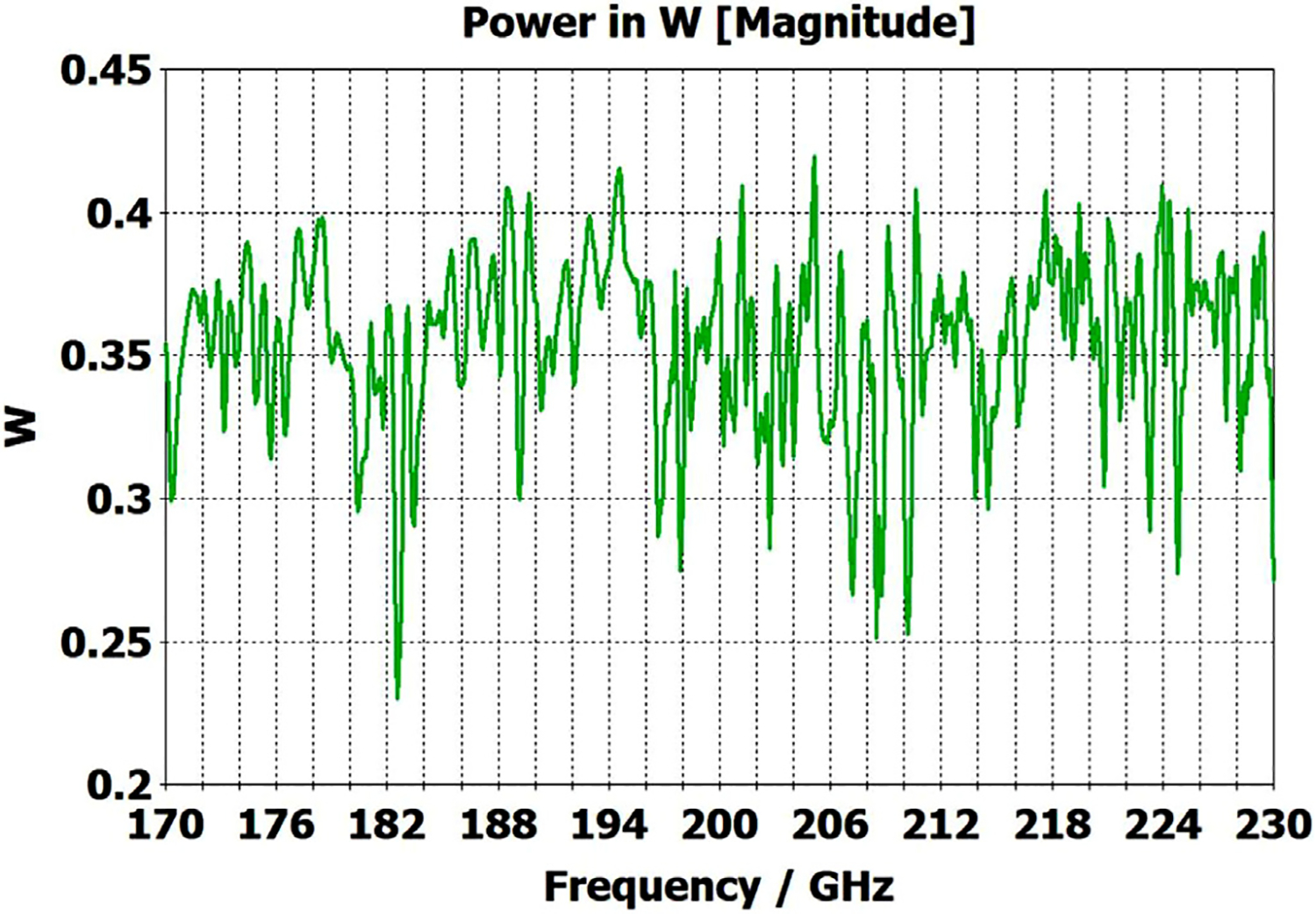
P_A_ for the output port for the same model as for Fig. 12 (large defects) except with numerically optimized corrugation parameters.

Fig. 13 shows the substantially improved results when periodicities are increased to 0.42*λ* in the first OMWG and 0.4*λ* in the second OMWG, groove depths are increased to 0.27 *λ* in the first OMWG and 0.32*λ* in the second OMWG, and relative groove widths are reduced to 0.28 in both OMWGs, with other parameters the same as for the case shown in Fig. 11. We will not attempt to present a theoretical basis for these parameters, and we emphasize that our focus has been on OMWGs smaller by factors of 2–6 relative to *λ* than those of most prior studies and usually in concert with large smooth downtapers, though in the next section we present some results on OMWGs with very small downtapers.

We should note that it was from the optimization of performance with the large defects (as in Figs. 12 and 13) that we settled on the diameters (8 mm, 4.8 mm, 3.4 mm, and 2.4 mm) that were then used for all of the cases shown in this set of simulation results. Different values for the diameters, but always beginning near 8 mm and ending in the 2.2–2.4 mm range, gave poorer results for most of these cases. Several runs without symmetry planes gave results nearly indistinguishable from those using two symmetry planes, confirming the theoretical validity of the solver using such to speed up the runs. One of these runs with no symmetry planes was with the larger asymmetric ring defects, and it did not show evidence of any rotation of the plane of polarization, which surprised us. Apparently, a more extended cylindrical asymmetry is needed to significantly rotate the plane of polarization.

We found that corrugation parameter optimization with OMWGs about half the lengths used in the mid-length cases reported here remained approximately optimum as the lengths were increased, so short versions were used to get close to the final optimum parameter values.

**Fig. 14.**
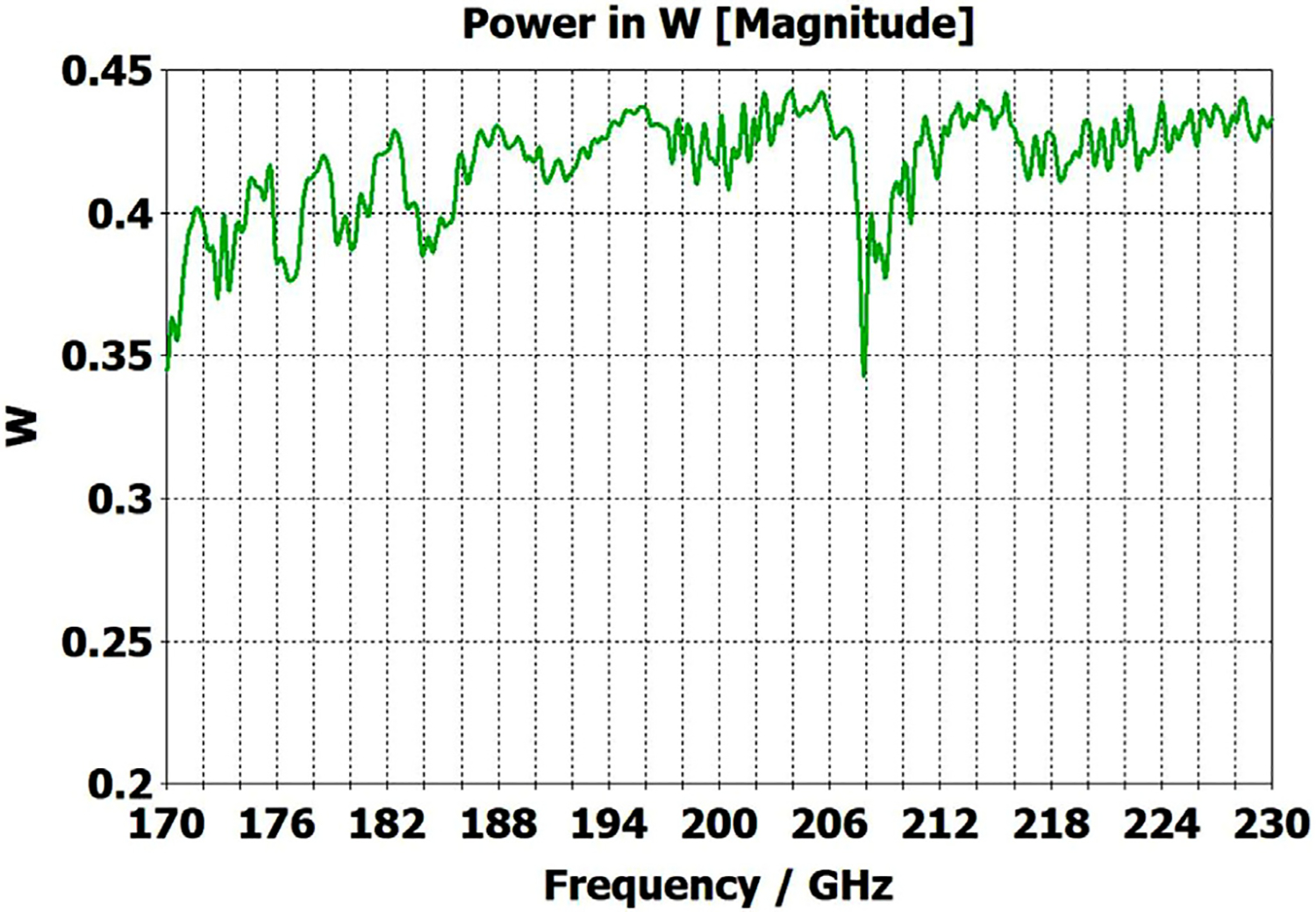
P_A_ for the output port with the same (numerically optimized) corrugation parameters as for Fig. 13 (mid-length 7-section model) but now with small manufacturing errors, as used in Fig. 11.

When the defect sizes are then made small (as were used in Fig. 11) without changing anything else, the transmission spectrum with the optimized corrugation parameters is rather amazing, as seen in Fig. 14. In this case, where the primary wave front reaches port_2 ~0.75 ns after excitation at port_1, the energy in this 28M-cell mesh decayed 30 dB in only ~5 ns, which still captures a sufficient number of secondary and tertiary reflections to obtain a well resolved power spectrum.

**Fig. 15.**
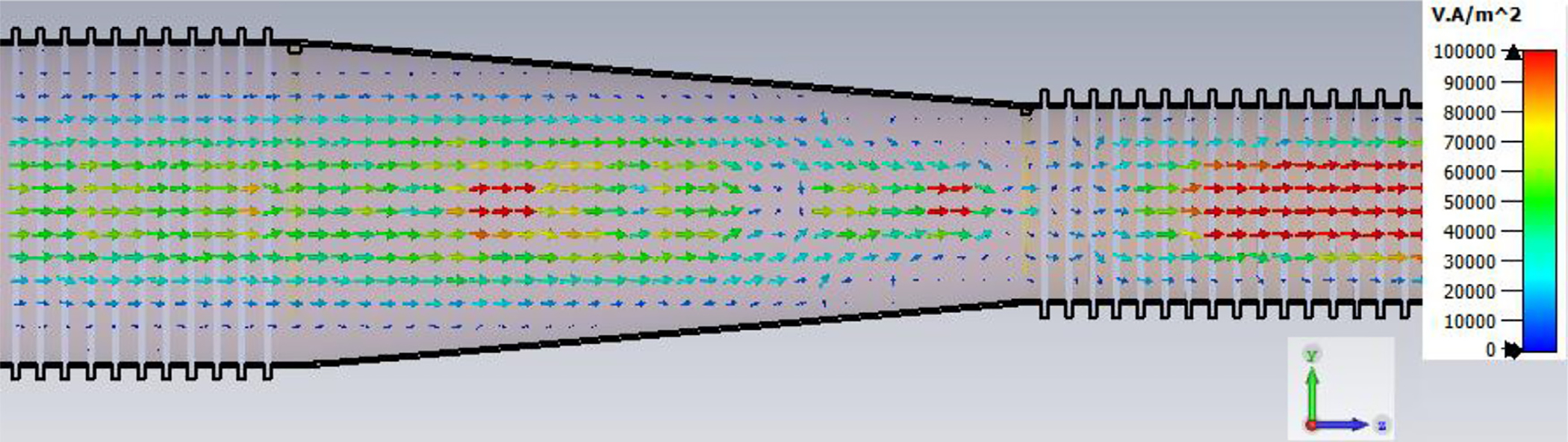
Power flow on the *x* = 0 plane at 200 GHz through the first downtaper for the model of Fig. 13 – large defects (visible near the ends of the downtaper) and numerically optimized corrugations. Red is > 1E5 W/m^2^.

**Fig. 16.**
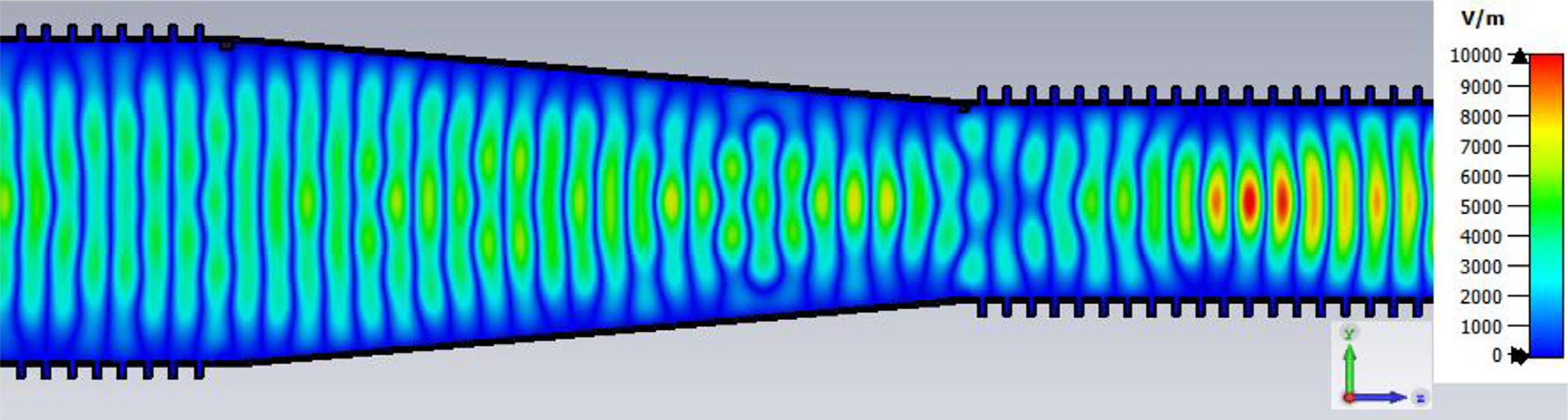
Eabs for the same region as in Fig. 15. Red is > 1E4 V/m.

The Poynting vector plot (power flow density, which is not dependent on the chosen phase) in Fig. 15 on the *x* = 0 plane at 200 GHz in the vicinity of the first downtaper from the model used for Fig. 13 (large defects) helps one understand why smooth straight downtapers can, within limits, work very well. Here, one sees very low power density near the walls all the way through the large downtaper and into the second OMWG. The situation here was similar at 195 GHz. *Apparently, the collective evanescent fields from the numerically optimized corru-gations keep the power flow low near the walls and thus reduce losses and the effects of defects there.* An E-magnitude snapshot for the same view, shown in Fig. 16, exhibits the quasi-Gaussian profile expected for HE_11_ over most of this region, though with some obvious mode conversion and beating in places.

**Fig. 17.**
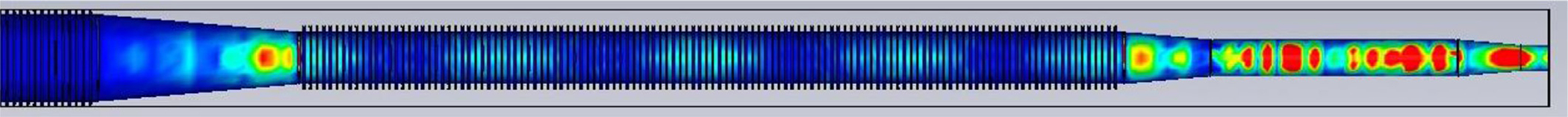
Surface power density at 200 GHz for the case in Fig. 13. Red is > 1E5 W/m^2^.

The surface power density at 200 GHz depicted in Fig. 17 shows highest loss density in the smooth regions even though those regions had conductivity 1.5E7 S/m while the corrugated regions had conductivity 5E6 S/m. Still, the dissipation from the corrugated sections (typically ~0.05 W) was about half of the total typical surface dissipation. It could have been reduced by gold plating, but the amplitude of the spikes in the transmission spectrum (Fig. 13) would have been greater.

**Fig. 18.**
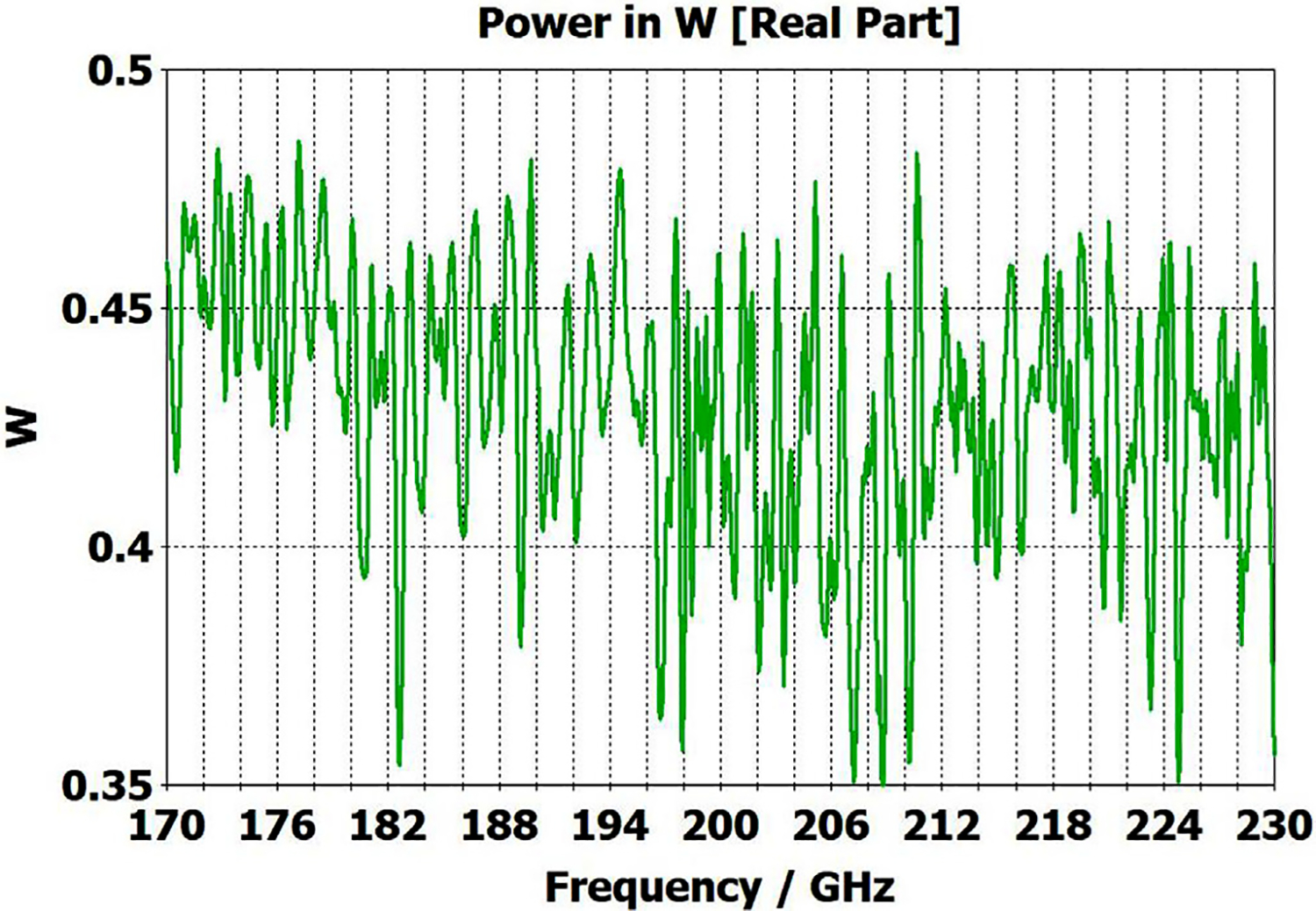
Power accepted at the in-port for the case of Fig. 13.

The port_1 power-accepted (P_A_) graph in Fig. 18 shows that over half of the difference between the 0.5 W excitation and that seen at the outport in Fig. 13 is explained by reflected power – the difference between 0.5 W and what is seen in this graph. Virtually all of this comes from the large asymmetric defects (visible, though exaggerated, in Fig. 9), not from the downtapers and other effects. This conclusion is supported by the observation that the mean S11 for the case shown in Fig. 14 (with small defects) was below −22 dB over the full bandwidth, and later we present results for smooth waveguides further supporting this determination. Several separate runs showed that all of the defects contributed to the degradation in performance of the model of Fig. 13, though defects in the second OMWG were a little more significant. Clearly, defects everywhere need to be kept as small as practical.

**Fig. 19.**
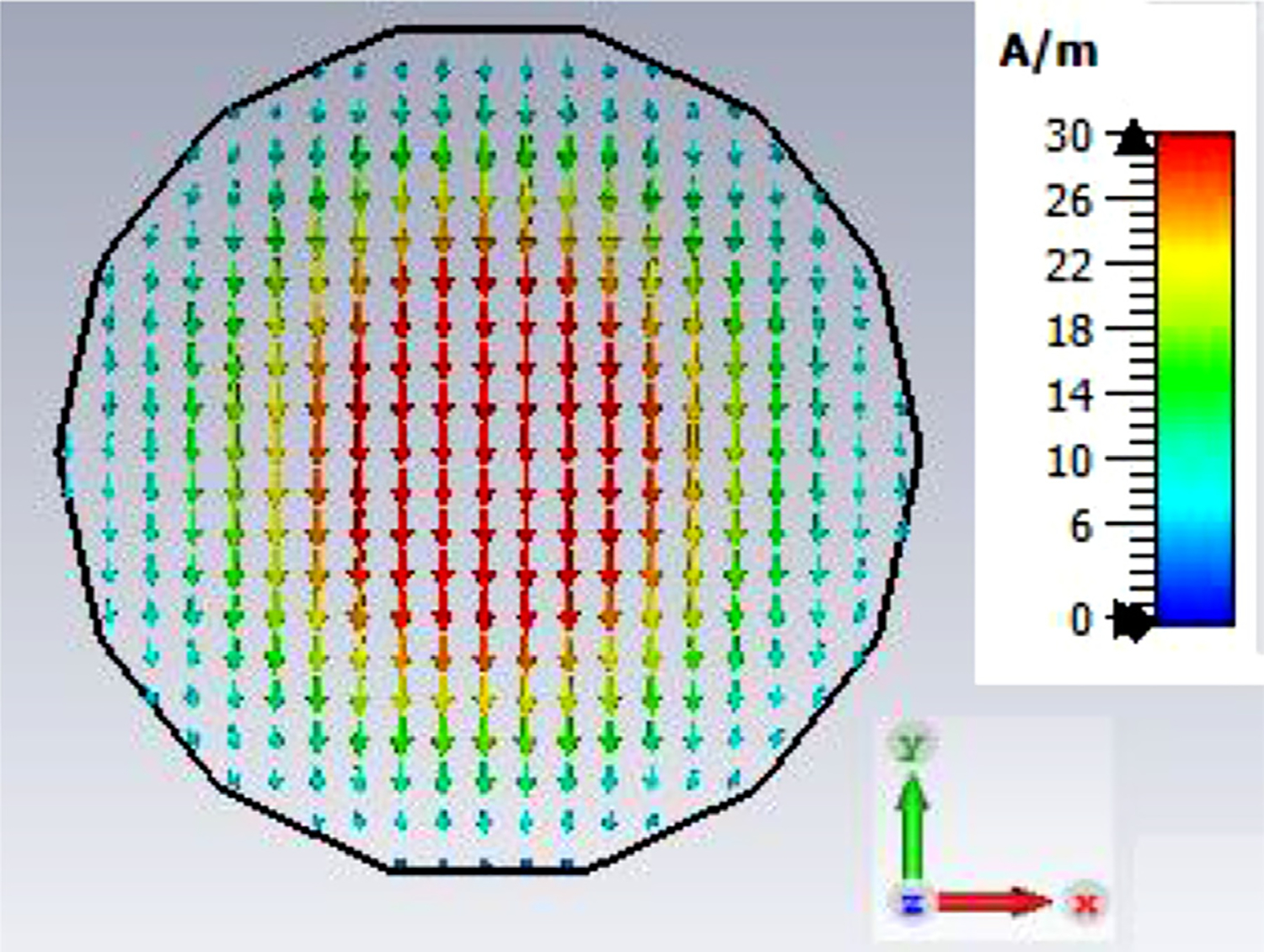
Magnetic H-field vectors at the output port. Red clamped at 30 A/m. Central maximum is 38 A/m.

Looking further at the case of Fig. 13, the H field near the out-port, depicted in Fig. 19 at 200 GHz, is very much as expected at this point for predominately TE_11_. (The out-port was round, not a polygon, as appears here after the mesh is built and run in CST.)

**Fig. 20.**
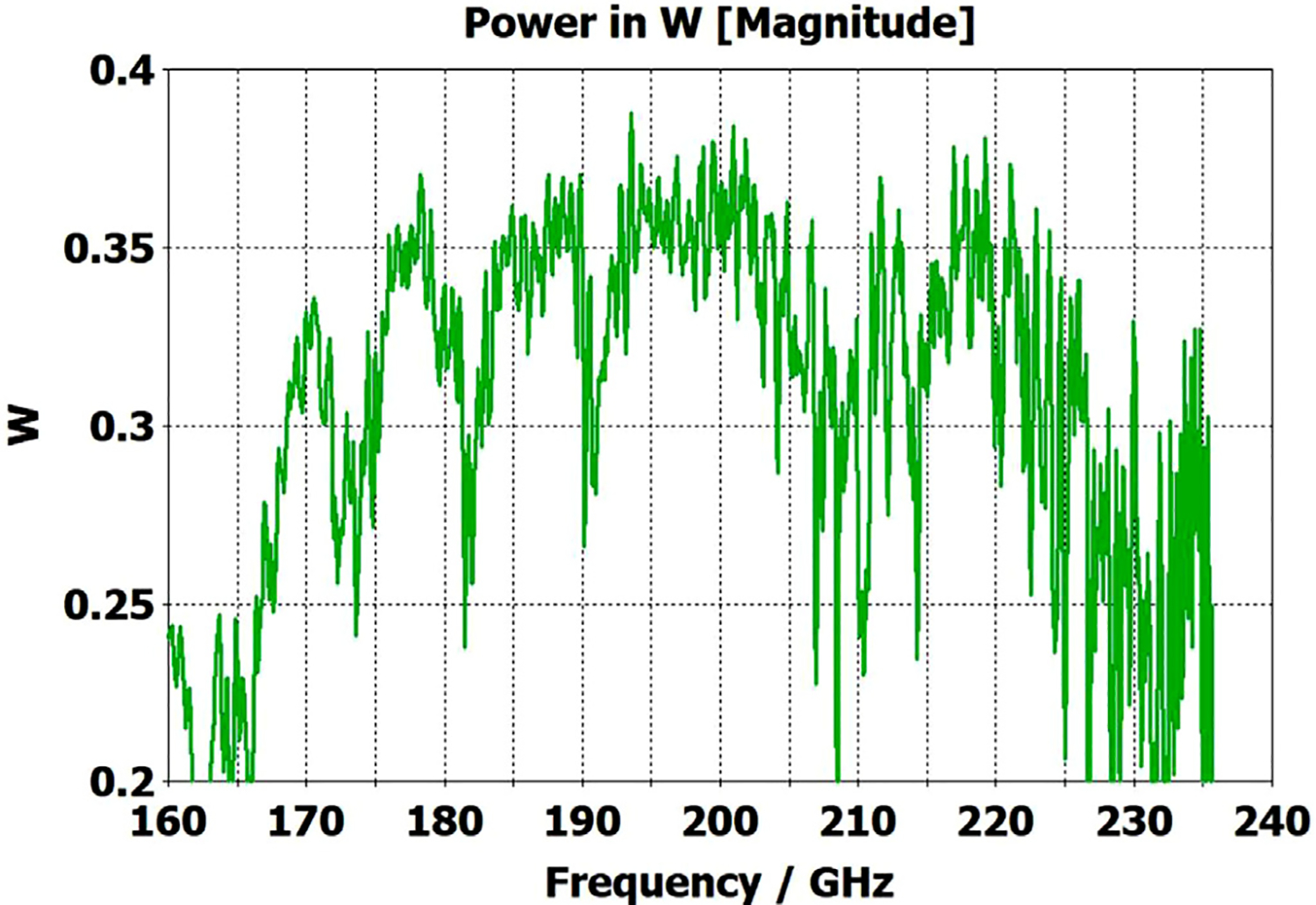
P_A_ at the output port for a long 7-section model, similar to that for Fig. 13 (with large defects) except with greatly increased lengths of the OMWGs and with out-port diameter reduced to 2.2 mm.

Fig. 20 shows that the same optimization continues to work “*well*” as the lengths of the two corrugated OMWGs are increased to 357 mm and 135 mm respectively, even with the out-port diameter reduced to 2.2 mm. This 72M-cell mesh had a 3.5-hour run time on a computer as described earlier. Readers may question the use of the word “*well*” here, but we note the waveguide includes six rather large manufacturing errors, three smooth downtapers, and a small (3.4-mm) smooth straight section. While it is theoretically possible to convert the source TE_11_ + TM_11_ efficiently to just TE_11_ at a final fundamental-mode waveguide, that requires the two modes to arrive at a suitable conversion structure with a specific phase relation, and in general that is not possible in the intended application, at least when manufacturing errors are present. Thus, good broadband transmission can only be achieved if the final waveguide to the out-port is large enough for both the TE_11_ and TM_11_ modes to propagate. Hence, the out-port waveguide diameter must be at least 2.2 mm for good broadband transmission in the 180–220 GHz range. The mean attenuation for this OMWG with the downtapers – not considering that reflected – at 200 GHz was a rather impressive 3.5 dB/m, which is three times better than for standard commercial fundamental-mode waveguide. Finally, we note that we briefly explored the possibility that some improvement might be possible from a smoothed profile at the ends of the tapers, as others have reported [30], but we did not see enough benefit in our regime of interest to justify the additional manufacturing complications.

## 5. Simple corrugated OMWGs with minor downtapers and defects

**Fig. 21.**
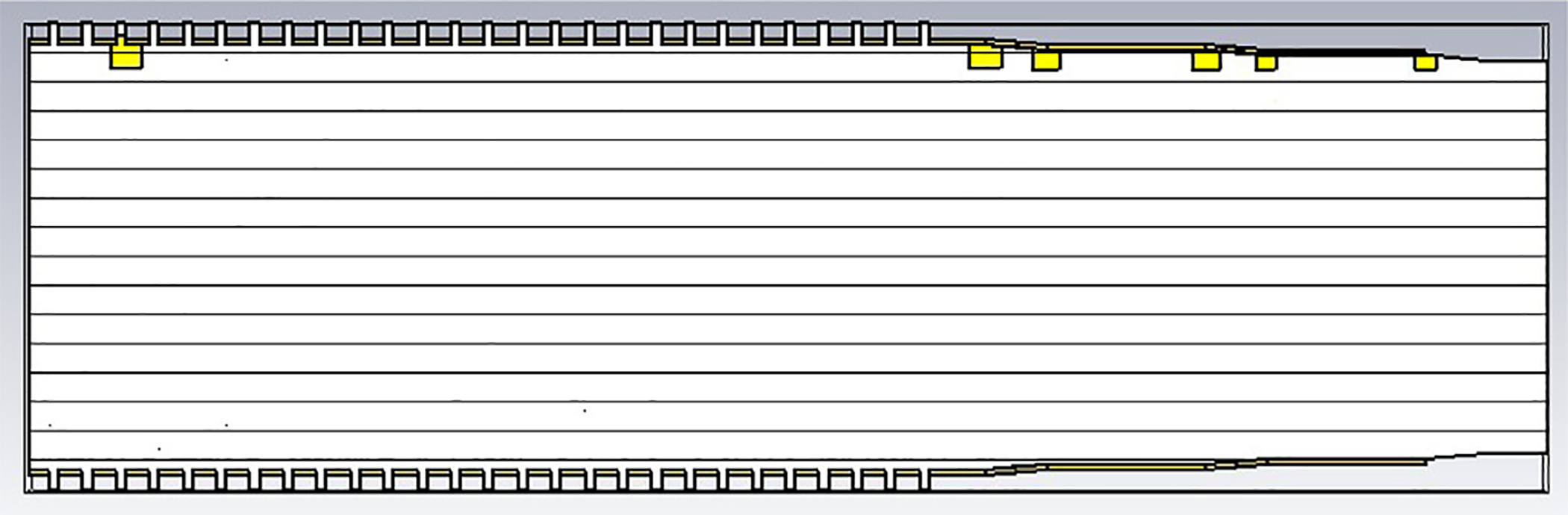
Example-3 geometries, beginning with a single corrugated OMWG (though shown here with the first OMWG severely shortened) followed by three minor downtapers to very short smooth OMWGs.

We were interested in seeing how the separate corrugated OMWGs in the model for Fig. 13 behaved when essentially alone, followed by minimal downtapers with small defects. This can be done with a model similar to that shown in Fig. 21 except with the first OMWG relatively longer than pictured and with smaller defects.

**Fig. 22.**
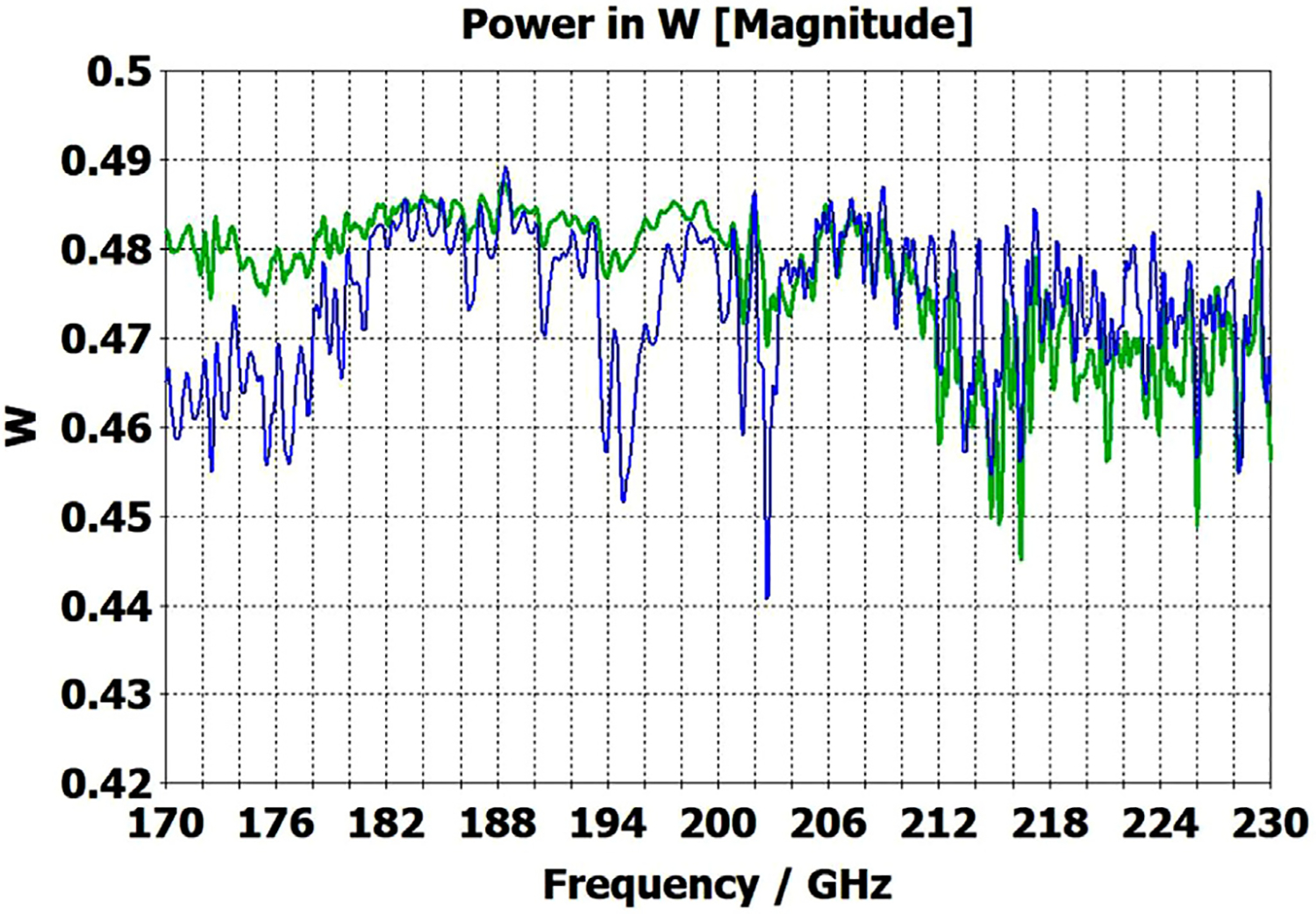
P_A_ for the output port for a model approximating just the first OMWG in the mid-length model for Fig. 13 (*a* = 2.67 *λ*, *d* = 0.27 *λ*, *p* = 0.42*λ*, *w/p* = 0.28, moderate defects), and translating to ~1 dB/m below 200 GHz. The green trace is for HE_11_ excitation, and blue trace is for TE_11_ excitation.

To look at just the first (8 mm) OMWG in the model for Fig. 13, we used a geometry similar to that shown in Fig. 21 where the lengths of the first, second, third, and fourth OMWGs were 78 mm, 3 mm, 3 mm, and 1.1 mm respectively, with respective IDs of 8 mm, 7.8 mm, 7.6 mm, and 7.4 mm (the out-port). The corrugation parameters for the first OMWG were the same as for the model of Fig. 13. The rest of the waveguide was smooth, and the defect sizes and offsets were 0.2 mm, 0.1 mm, and 0.1 mm. Fig. 22 shows power accepted at the output port for HE_11_ excitation compared to TE_11_ excitation for this model, in which conductivity in the corrugations was 5E6 S/m, but conductivity of the smooth sections was set at 5E8 S/m to make their losses negligible. For the HE_11_ case, the total power loss in the OMWG at 200 GHz was 0.01 W (mean reflected power was ~0.007 W), corresponding to ~1 dB/m. This is a little better than theoretical attenuation for pure TE_11_ in smooth waveguide of this size and conductivity (1.5 dB/m for *σ*=5E6 S/m) [4,6,7]. The previously derived expression [16] for HE_11_ attenuation in corrugated waveguide with rectangular grooves by standard guidelines (*d* = 0.25*λ*, *p* = 0.333*λ*) predicts 0.4 dB/m for the relevant conditions (200 GHz, *a* = 4 mm, *σ*=5E6 S/m). The traces in Fig. 22 clearly show why TE_11_ excitation is not preferred, though it is usable.

The HE_11_ trace in Fig. 22 suggests optimum transmission might have been better centered near the target frequency in this simple case (very little diameter reduction and much shorter smooth sections) for *d* = 0.25*λ*, but the spectrum of Fig. 20 for the complete four-OMWG waveguide with three substantial downtapers shows the parameters used seemed to be optimum for 194–206 GHz.

When the corrugation parameters in the above model were changed to standard guidelines and the size of the defects were reduced by an order of magnitude, the port_2 P_A_ response (not shown) from 160–240 GHz (with HE_11_ excitation) looked essentially as flat as that in the green trace (HE_11_ excitation) in Fig. 22 in the 170–200 GHz range, with about the same mean mid-band attenuation, ~1.1 dB/m.

The radii in the above model were then changed to 2.4 mm, 2.3 mm, 2.2 mm, and 2.1 mm for the four sections respectively, with corrugation parameters as used on the second OMWG in the model for Fig. 13 to see how this OMWG behaved essentially alone with large defects.

**Fig. 23.**
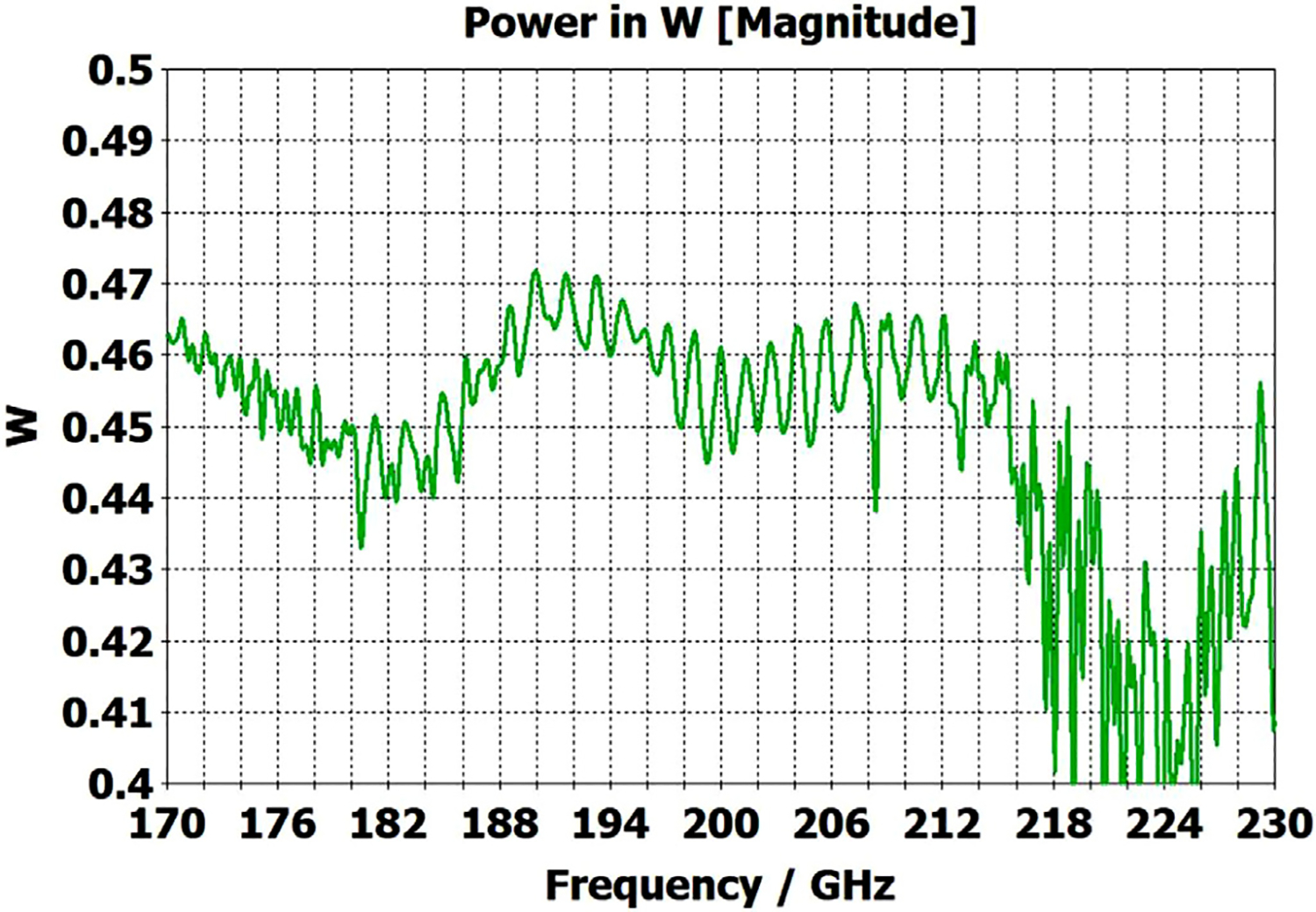
P_A_ for the output port of an approximation of just the second OMWG for Fig. 13, driven by HE_11_, with large defects.

Fig. 23 shows power accepted at port_2 for HE_11_ excitation at port_1 for this case. The typical mid-range power loss in the corrugated surfaces was ~0.9 dB/m, which is about a factor of two better than expected from the referenced derivation and more than a factor of two better than for pure TE_11_ in smooth waveguide of this size and conductivity. This suggests that even with large defects relatively small corrugated OMWGs may perform better than expected from theoretical derivations if numerically optimized. This contrasts with what is often seen in practice for larger corrugated OMWGs. The poorer performance seen above 216 GHz in Fig. 23 suggests there may be room for further improvement in these corrugation parameters, but keep in mind that this 4.8-mm OMWG had large defects.

## 6. Length effects in simple corrugated OMWGs

**Fig. 24.**
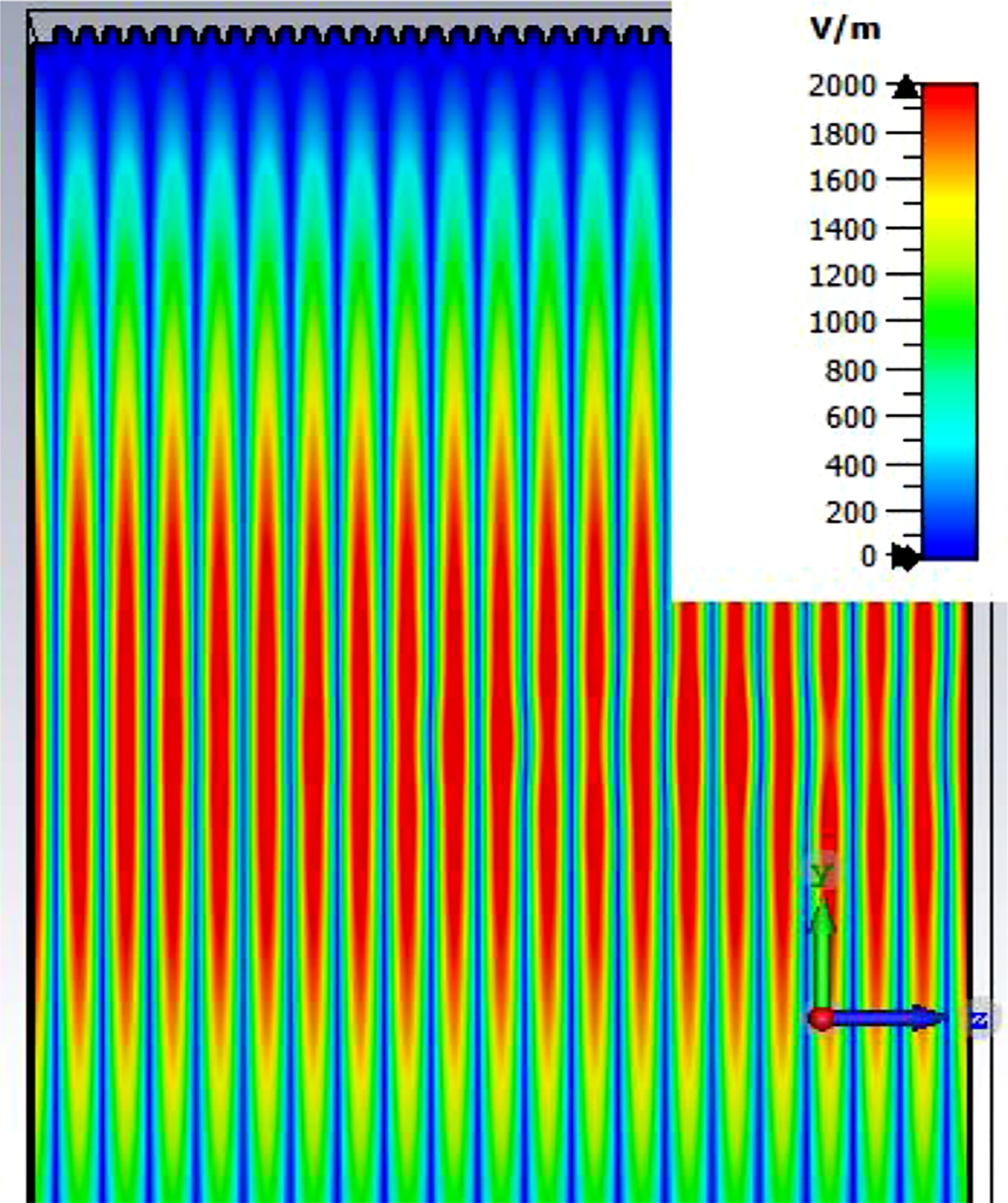
Eabs for the +*y* half of a very short version (12-mm long, 35 grooves) of the MIT 19-mm 330-GHz OMWG. Red is > 2000 V/m.

**Fig. 25.**
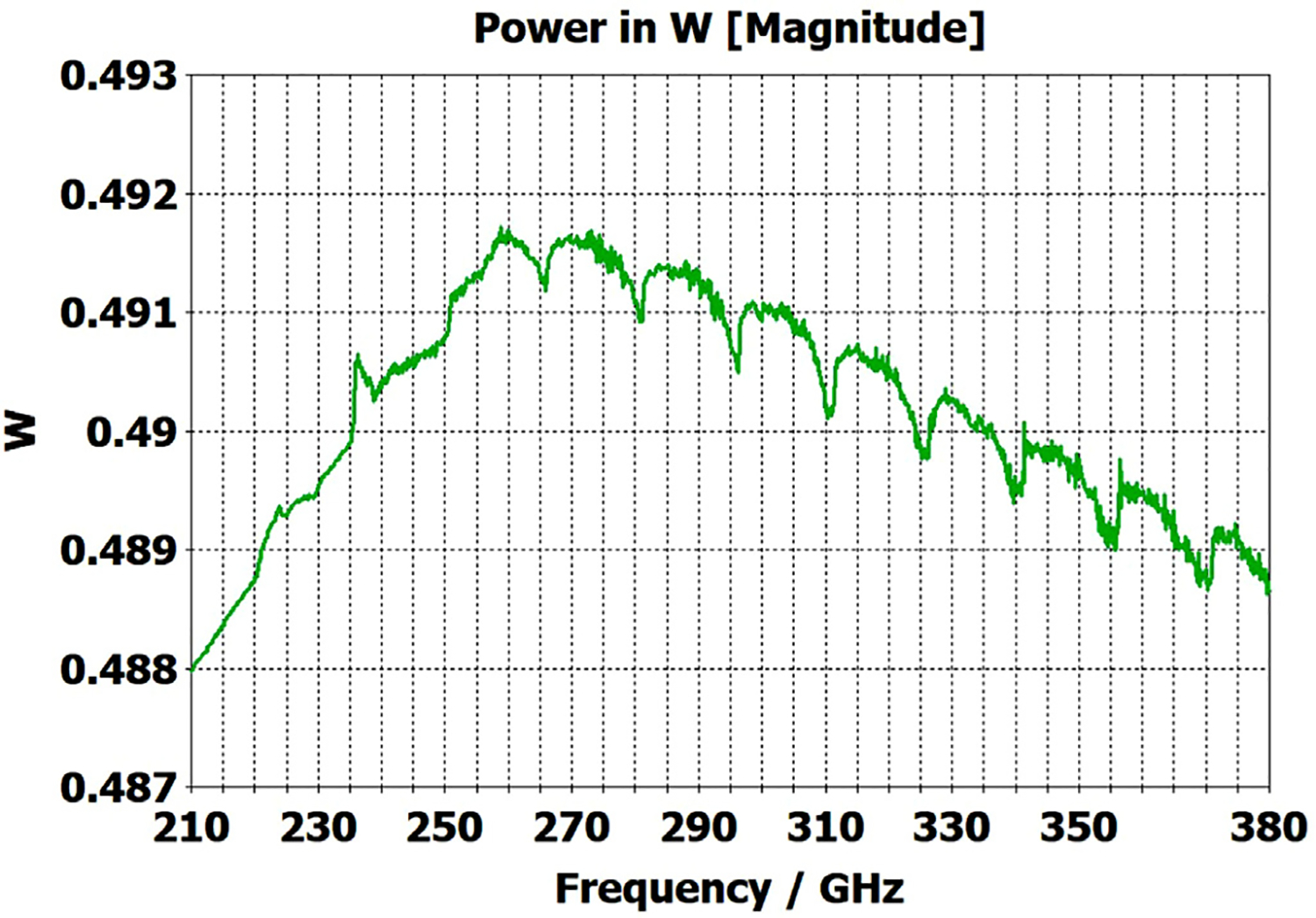
P_A_ for the output port of a 312-mm length of the 19-mm 330-GHz MIT OMWG as in [16], and translating to ~0.2 dB/m near 250 GHz. Note the remarkably wide bandwidth of large corrugated OMWGs.

The differences from what we expected from published expressions for attenuation in these simulations prompted us to simulate two large corrugated OMWGs described in previously reported experiments as a possible means of validating the CST TD software for large OMWGs. Nanni et al. reported experimental results at 250 GHz for a 19-mm brass OMWG (*a* = 9.5 mm) optimized for ~330 GHz with helically grooved trapezoidal corrugations (cut by a tap). The reported measured attenuation (by a radiometer) at 250 GHz (*λ* = 1.2 mm, *a*/*λ* = 7.9) for a 2.5-m length was 0.131 dB, or 0.052 dB/m [16]. While the reported HFSS simulation for a single rectangular groove was for *d* = 0.227 mm, *p* = 0.3175 mm, and *w/p* = 0.5, we instead used the reported mean fabricated depth of 0.22 mm and mean *w/p* of ~0.6 from the photo (Fig. 7 a in [16]). Our model included two small (0.02-mm) defects of the type described earlier. We assumed effective surface resistivity of 5E6 S/m, as the groove photo showed a rather rough surface. An Eabs field snapshot for a very short version of the model is shown in Fig. 24. Here, the total length was 12 mm; and the simulation showed a nicely flat transmission from 210–380 GHz with a total attenuation of 3.9 dB/m at 250 GHz (and similar at 300 GHz), or *~75 times* the experimentally measured value for a 2.5-m length. So, we increased the length to 32 mm (99 grooves) and saw a similarly nice transmission spectrum, with loss ~1.6 dB/m at these frequencies. Finally, we increased the length to 312 mm (979 grooves, 250 M mesh cells) and (22 h later) got the P_A_ spectrum shown in Fig. 25. Here, the total loss at 250 GHz was ~0.2 dB/m, or 4 times the reported value for a waveguide ~9 times longer and 8 times the value predicted by Nanni’s expression with *R*_S_ = 0.5 Ω. All of these were run to −35 dB energy decay. We did not go beyond 312 mm length in this 19-mm OMWG because the tool used in the CST geometry builder would not permit more than 1000 repetitions (grooves).

By making the small defects three times larger and seeing negligible difference, we concluded the high loss in the short cases was not from the defects. We also confirmed that the mesh was adequately refined and that the results were not significantly dependent on various solver parameters or in-port and out-port details. The corrugations for the above simulations (and in later simulations, unless noted otherwise) were simple trapezoids with sharp corners. We investigated the addition of small radii to the corners of the teeth, at both their inner and outer edges, and saw that had relatively minor effects (under 25%) in calculated losses for a given length but it added an enormous amount of time to the simulations.

Useful published data are available on the ITER LFSR OMWG, which is an aluminum OMWG optimized for 100 GHz with helically grooved trapezoidal corrugations (cut by a tap), with *d* = 0.75 mm, *p* = 0.73 mm, *w* = 0.5 mm, *w/p* = 0.685, and *a* = 31.75 mm. Measured loss at both 35 GHz and 50 GHz on a 62-m length that included 20 miter bends were reported [44]. At 50 GHz (where the entrance beam diameter was 2 cm, *λ*=6 mm, *a*/*λ*=5.29, *d*/*λ*=0.125, *p*/*λ*=0.122, and *w*/*λ* = 0.084) the total loss was 2.5 dB, or 0.04 dB/m. They calculated the loss per miter bend to be 0.08 dB, implying 0.014 dB/m in the waveguide itself. They indicated theoretical loss without the miter bends for aluminum (*σ* = 3.7E7 S/m, DC) was expected to be ~0.002 dB/m at 50 GHz and ~0.0003 dB/m at 100 GHz. One gets similar numbers from Nanni’s formula by assuming *R*_S_ is 2–3 times its classical value.

**Fig. 26.**
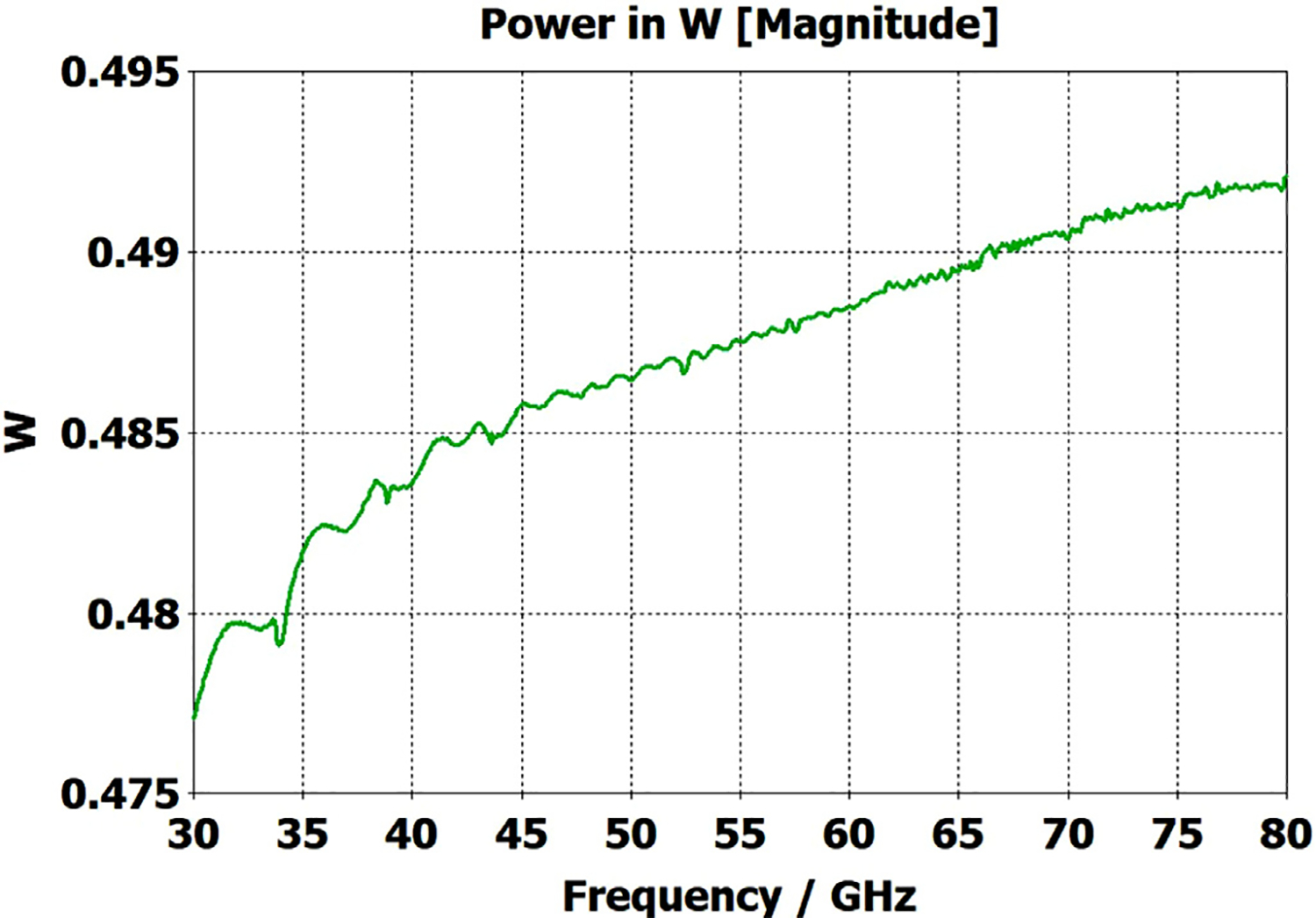
P_A_ for the output port of a 725-mm length of the 62-mm ITER OMWG.

We then simulated the above ITER OMWG, with *σ*=1E7 S/m, at several different lengths with grooves as reported except not helically cut. For a short case (66 mm long) attenuation at 50 GHz was ~0.02 dB, or 0.32 dB/m. For a mid-length case (270 mm long) loss at 50 GHz was ~0.06 dB, or 0.21 dB/m. Transmission for the longest case we could readily simulate (725 mm) is shown in Fig. 26, where loss at 50 GHz was found to be 0.12 dB, or 0.16 dB/m, which is ten times what they determined in a waveguide in which the individual sections were 5–10 times longer. Poynting vector plots showed the typical power density near the out-port (at 50 GHz) to be ~50% of the central peak value at a radius ~0.38*a,* and ~1% of the central value at a distance ~*λ*/4 from the wall. Our attenuation results were quite close to those recently reported (0.11 dB/m for rectangular grooves) by another group [45] for simulations of a waveguide of the same ID at 42 GHz (except 0.5 m long) in contrast to the results reported by the ITER LFSR group.

The above results suggest there may be a previously unreported length dependence in the attenuation – in dB/m – that dramatically increases attenuation when the length to diameter ratio is not very large. This is distinctly different from the standard diffraction effect at the in-port [15,17,43], as the attenuations we report are always based on the difference between power accepted at the inport and power accepted at the outport.

**Fig. 27.**
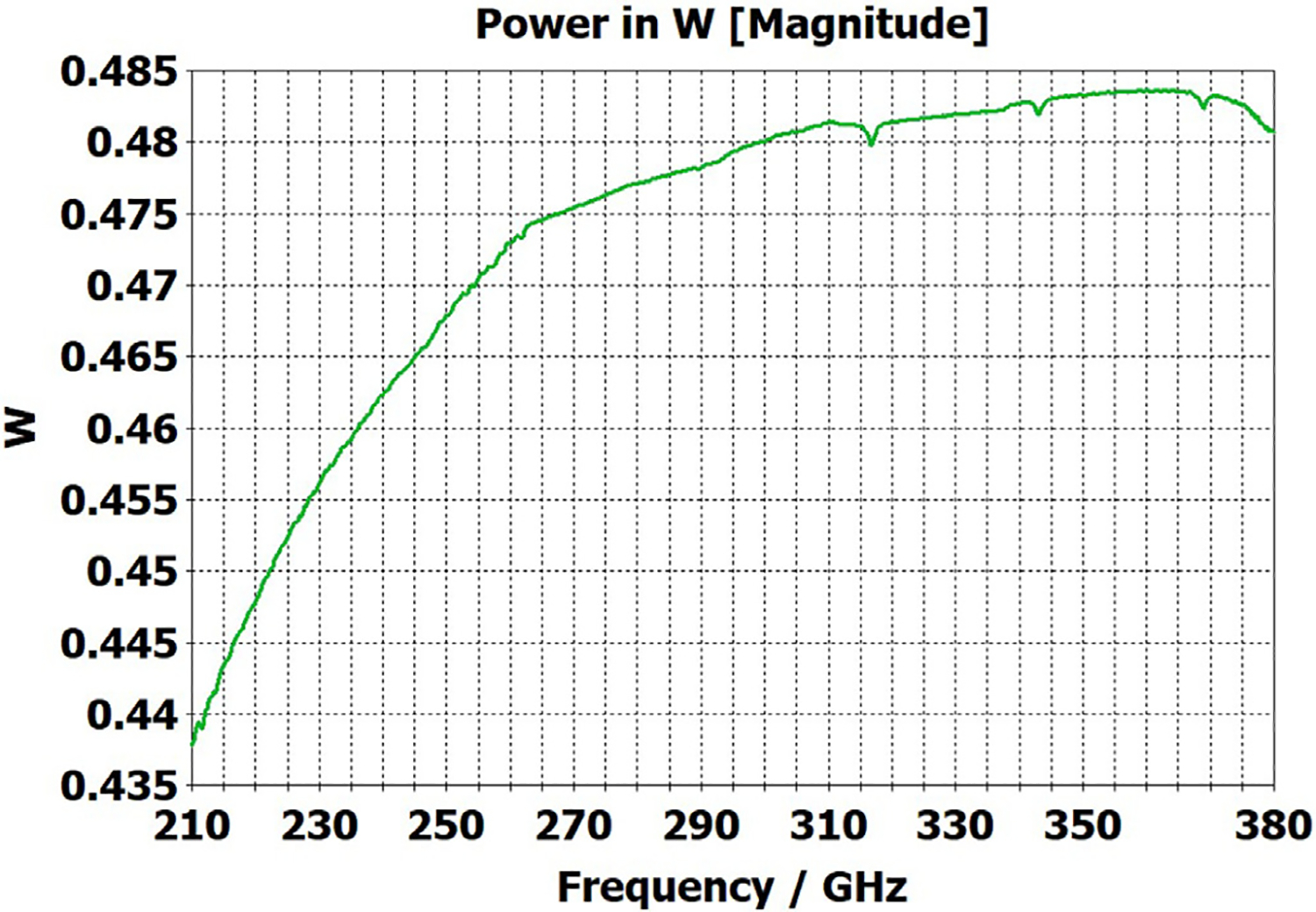
P_A_ at the output port of a 312-mm length of the 6-mm 330-GHz OMWG as described in the text.

To test our hypothesis that the loss in corrugated waveguides approaches the theoretical value only as the ratio of length to diameter becomes quite large, we reduced the diameter of the above “MIT” OMWG from 19 mm to 6 mm and set metal DC conductivity at 5E6 S/m while keeping all other parameters the same. This waveguide was simulated for lengths of 12 mm, 40 mm, 100 mm, and 312 mm. Attenuations for these four lengths were 4.4 dB/m, 1.54 dB/m, 0.71 dB/m, and 0.48 dB/m respectively at 300 GHz. Transmission results for the last case are shown in Fig. 27. The published expression [16] predicts attenuation of 0.48 dB/m at 300 GHz for effective surface resistance R_S_ of 0.5 Ω. The theoretical value of *R*_S_ for perfect metal surfaces of 5E6 S/m at 300 GHz is 0.49 Ω, and the effective value with typical surface quality is ~0.8 Ω. Clearly, the agreement of the published expression with simulation results for long waveguides (length/diameter > 40) can be quite good in some cases. However, with corrected values for *R*_S_ it apparently overestimates attenuation in small waveguides (by perhaps ~30% for *a*~3*λ*) and it underestimates attenuation in large waveguides – sometimes by an order of magnitude or more.

## 7. Some alternatives to corrugations in OMWGs

### 7.1. Smooth round waveguides with downtapers

**Fig. 28.**
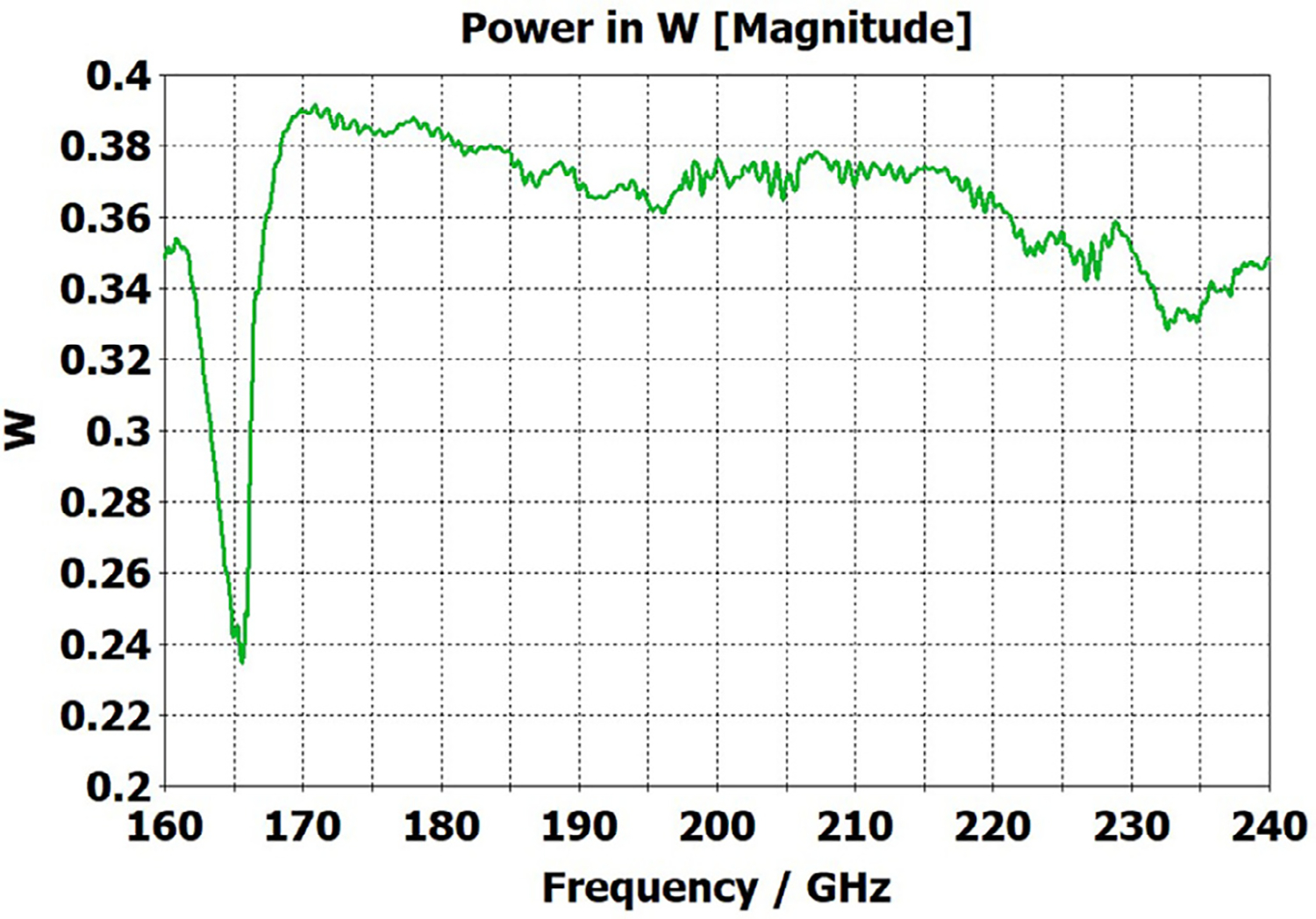
P_A_ at the output port for a long smooth 7-section waveguide with three downtapers but with no defects, with lengths and diameters the same as for Fig. 20.

**Fig. 29.**
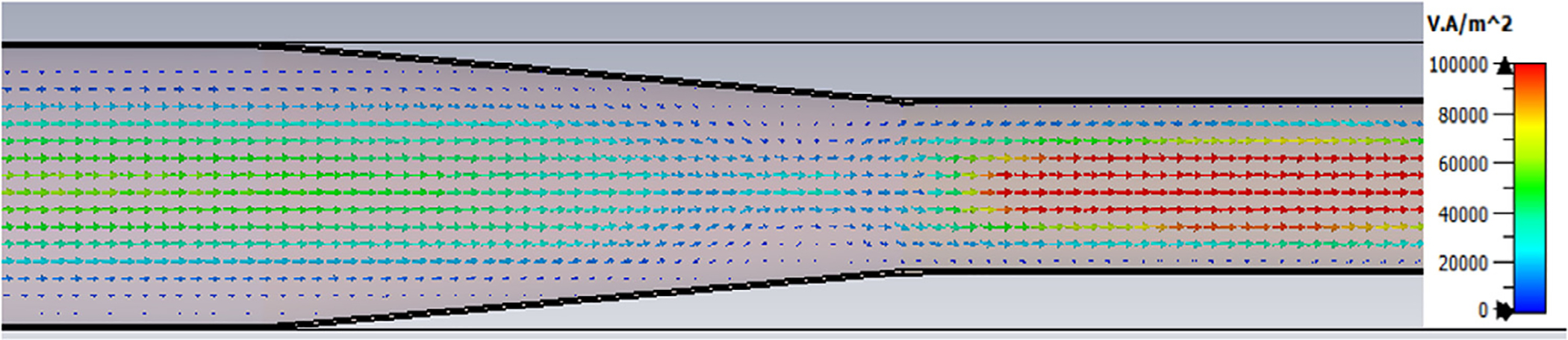
The Poynting vector in the first downtaper of the smooth waveguide for the case of Fig. 20 – except no defects – at 197 GHz.

The simplest alternative to corrugations is the classic smooth cylindrical OMWG, as discussed earlier in this paper (see Table 1). Surprisingly, CST simulations showed smooth OMWGs, excited by HE_11_, with downtapers and dimensions as in the earlier models for Figs. 11–14, *worked even better* – *as long as the defects were very small and the excitation was perfectly centered*. The beam intensity near the walls was much higher (as expected) than in the corrugated waveguides, *but response was flatter and mean loss was less!* Fig. 28 shows port_2 P_A_ for a long OMWG with the same dimensions as used for Fig. 20, but now perfectly smooth and with no defects. The Poynting vector for a portion of the model is depicted in Fig. 29.

So why do anything more complicated for small OMWGs than smooth round waveguides with smooth downtapers? The answer is seen when imperfections are added. Smooth waveguides in our size range of interest (0.7 *λ*<*a*<4*λ*) with very small imperfections (<0.01*λ*) and with multiple smooth downtapers to *a*>0.75*λ*, as employed in the model of Fig. 14, continued to perform better than seen with the optimized corrugations in Fig. 14. However, large imperfections, as employed in the models of Figs. 12 and 13, caused substantially poorer performance in smooth waveguides with downtapers than in waveguides of similar size with optimized corrugations, both with respect to flatness and with respect to mean mid-band loss. We recognize that our remarkable *in silico* results for HE_11_-beam-fed smooth waveguides (which was validated many times, with many different models, and many different meshes) are counter to conventional experience-based observations, which conclude that smooth OMWGs with downtapers are always problematic. This belief could seem to be supported by the results presented in Figs. 4 through 7, but we should point out that those cases included both uptapers from fundamental mode and downtapers back to fundamental mode. In the discussion here of smooth overmoded waveguides, we are limiting our observations to cases like those presented in the previous section – HE_11_-beam-fed OMWGs with multiple downtapers to smooth waveguide supporting TE_11_ and TM_11_.

We suspect the basis for some experience-based beliefs can be traced to unaccounted-for imperfections. We note that the proxies we used for small imperfections (a few asymmetric conductive-ring-type imperfections, of size ~0.03*λ*) may not be good proxies for many typical small imperfections in practice, including the cumulative effects of a much larger number of smaller manufacturing errors and irregular surface oil and oxide films. Some appreciation for the possible effects of the latter may be gained from our *in-silico* studies of dielectric linings, as seen in the following section.

### 7.2. Dielectric linings

Thin dielectric linings (more often called coatings in the literature) have been reported to be an alternative to corrugation for support of HE_11_ in small waveguides for reduction of losses, dispersion, and trapped modes [46–48]. While a few remarkable results have been reported [48], the reported experimentally measured benefit of dielectric lining has more often been well below theoretical expectations [46, 47]. Presumably this has been from a combination of uncertainties in the theory and in deposition non-uniformities. We simulated a number of cases in the 160–240-GHz range with thin dielectric linings in a smooth 8.5 mm OMWG connected to input and output port diameters in the 2–2.8 mm range via a corrugated uptaper and downtaper respectively, with thicknesses ranging from *λ*/10 to *λ*/3, (where *λ*=*λ*_0_/*ε*_r_^0.5^) for several readily available flexible microwave substrate materials. Teflon (*ε*_*r*_ = 2.1, tan *δ*=0.0003), Rogers RO3003 (*ε*_*r*_ = 3.0, tan *δ*=0.0015), and Rogers 3850HT (*ε*_*r*_ = 3.15, tan *δ*=0.003) were evaluated. (We assumed loss tangents in the RO materials at 200 GHz were 50% higher than the published values for 10 GHz.) While we were able to obtain substantial improvement in flatness of response in relatively short models (up to ~30*λ* OMWG length) lined with 5-mil Rogers R03003 over bandwidths up to ~10%, those results did not hold up in longer models. We replaced the corrugated downtaper with a series of two and then three smooth lined downtapers and got some further improvement, though again only with short models and generally only going down to 2.8 mm at the out-port.

### 7.3. Suppressing cross-polar modes

As noted earlier, suppression of the cross-polar modes has previously been shown to be beneficial in round waveguides [8]. Since we always excited at phase = 0 (+*E* along +*x*, as in Fig. 10) we added a thin slab (0.127 mm thick) of the rigid microwave substrate material Taconic TLX-9 (*ε*_*r*_ = 2.5, tan *δ*=0.0019) on the *x* = 0 plane of the OMWGs to help damp cross-polar and some other trapped modes with minimal loss of the desired HE_11_ mode.

**Fig. 30.**
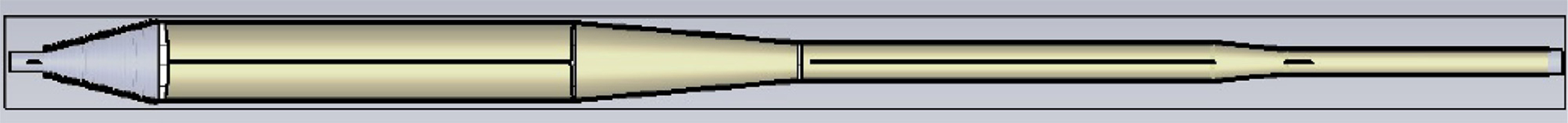
Cross-section view on the XZ plane of a simulated geometry beginning with a corrugated horn and including dielectric-lined smooth OMWGs, lined smooth downtapers, three lenses, and a thin dielectric slab along most of the *x* = 0 plane.

A cross-section view on the XZ plane of an example simulated geometry with two smooth downtapers is pictured to scale in Fig. 30. The full smooth length is lined with 5-mil Teflon. A thin Taconic TLX-9 slab is placed along most of the *x* = 0 plane, as seen. There are three lenses of Taconic TLX-9, focal lengths of 17 mm, 35 mm, and −35 mm respectively. Lenses at the beginning and end of tapered transitions were sometimes helpful. The lengths of the three OMWGs are 45 mm, 45 mm, and 30 mm respectively, with respective diameters of 8.3 mm, 4.1 mm, and 2.8 mm. Note that it includes substantial lengths of the smaller waveguides, getting it closer to one of our objectives of improved compatibility with the space constraints seen for MAS-DNP in high-field NB magnets; and its length is about mid-way between that used in most of our “short” and “mid-length” models. The corrugated horn was fed with TE_11_ from a 2-mm waveguide.

**Fig. 31.**
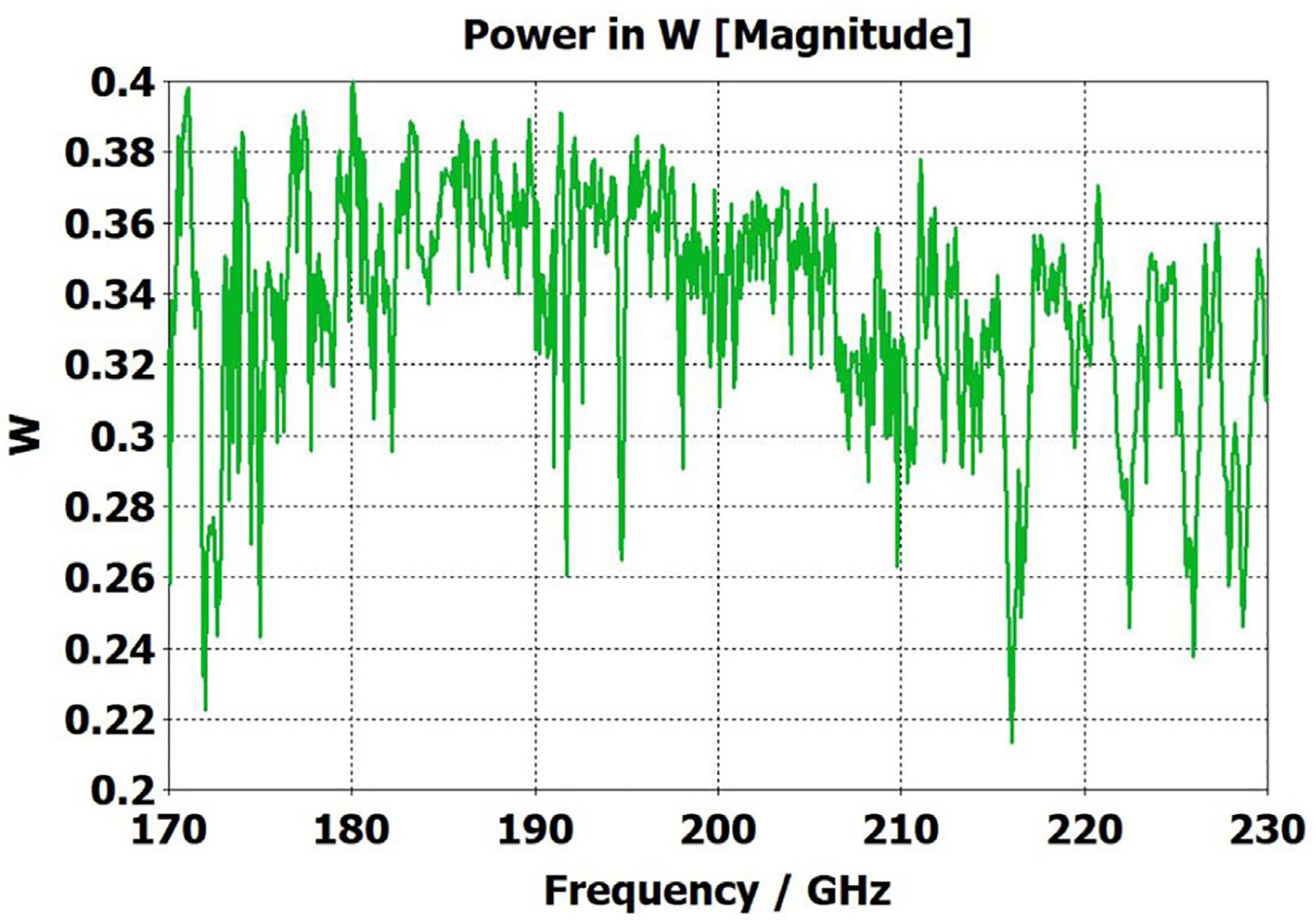
P_A_ for port_2 for the short 8-section model depicted in Fig. 30 (three lined smooth waveguides), tapering down to a 2.8-mm out-port.

The transmission performance of this model in Fig. 31 shows good overall performance though a few troublesome trapped modes remained near the region of interest. The graph shown here was from a simulation with two symmetry planes and 15 M mesh cells. The same model was run with a finer mesh and no symmetry planes (129 M mesh cells) and with a 31 M mesh with 2 symmetry planes. All gave essentially equivalent results – just minor differences in heights of the various spikes, with no changes in their locations. The strong mode at 194.8 GHz was found to be coming from the third lens. It could be moved out of the way by changing the lens thickness, but most of the trapped modes were related to various section lengths.

**Fig. 32.**
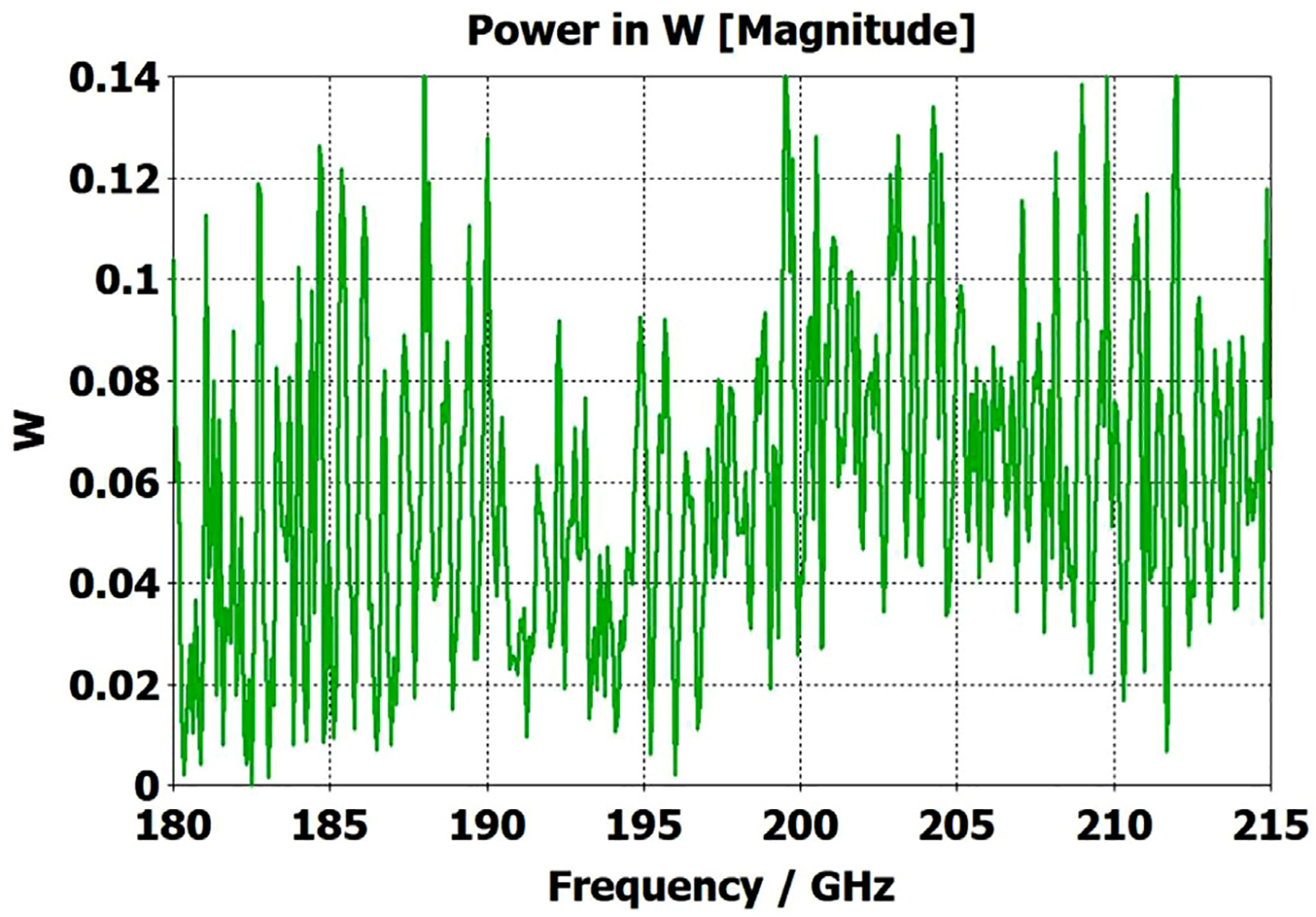
P_A_ for port_2 for a lined model similar to that in Fig. 30 but about 30% longer and with a third downtaper added, taking it to fundamental-mode output (1.5 mm diameter).

The length of the first OMWG in this model was still only ~10% of what would be needed in typical MAS-DNP probes; and when its length was increased, the trapped modes increased in number. It became clear that the measures employed here would not work with more realistic lengths or if the output needed to taper down to fundamental mode – and especially if both were needed. In fact, when a third downtaper was added to a model otherwise nearly the same as Fig. 30 that took its output down to 1.5-mm diameter in a final additional short section, the P_A_ at the output was only ~10% of that entering port_1, as seen in Fig. 32 with a very different vertical scale. Later we saw better results were possible by simply removing the linings. Changing from a corrugated horn to a spline-profile horn also helped a little.

We note many other alternatives have been reported for THz transmission with lower loss than fundamental-mode waveguides, most of which have been based on micro-structured “holey” *subwavelength* (meaning diameter <*λ*) dielectric fibers [48–55], as recently reviewed here [49]. However, these holey fibers can be leaky and require closely spaced mechanical supports, which increase leakage. With such, losses are typically in the 15–25 dB/m range at 400 GHz, and coupling losses into and out from these fibers is often ~6 dB at each end. Hence, none of these seemed well suited for use in MAS-DNP probes. Other reported THz waveguide alternatives [56–65] also had substantial deficiencies or drawbacks from our perspective. Our application requires: a waveguide with good confinement even when near external structures (whether dielectric or metal), compatibility with efficient excitation from a highly overmoded quasi-Gaussian TEM_00_ beam, ability to handle power up to a few watts, compatibility with tapered transitions, not too difficult to manufacture, and good broadband performance. So, we looked at some novel approaches.

### 7.4. Converting some trapped modes to TE_11_

Most of the power not getting to the fundamental-mode port_2 output in the better models similar to that of Fig. 30 (but longer, and without linings) was not appearing as reflected power at the in-port. Rather, it was being absorbed in trapped modes. There appeared to be a lot of conversion of TE_11_ to TM_11_ at the ends of the second and third downtapers (which then does not propagate in the output 1.5-mm waveguide below 250 GHz) and also some conversion to cross-polar modes. We hypothesized performance could be improved by preventing propagation of the primary cross-polar modes in the OMWGs, as is routinely done in some other applications of fundamental-mode circular waveguide [8], and that conversion of undesired modes to TE_11_ could be improved, with little attenuation of TE_11_ and HE_11_, by putting some copper foil patches along the *x* = 0 plane.

**Fig. 33.**
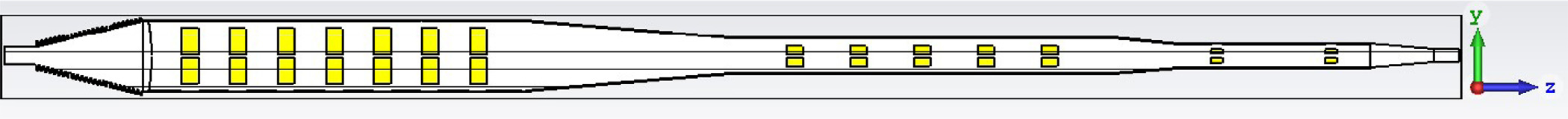
Cross-section view on the YZ plane showing how copper foil patches supported on thin dielectric slabs were positioned at various places in the smooth OMWGs (with no linings) on the *x* = 0 plane to suppress primary cross-polar modes with negligible effect on HE_11_ and TE_11_.

**Fig. 34.**
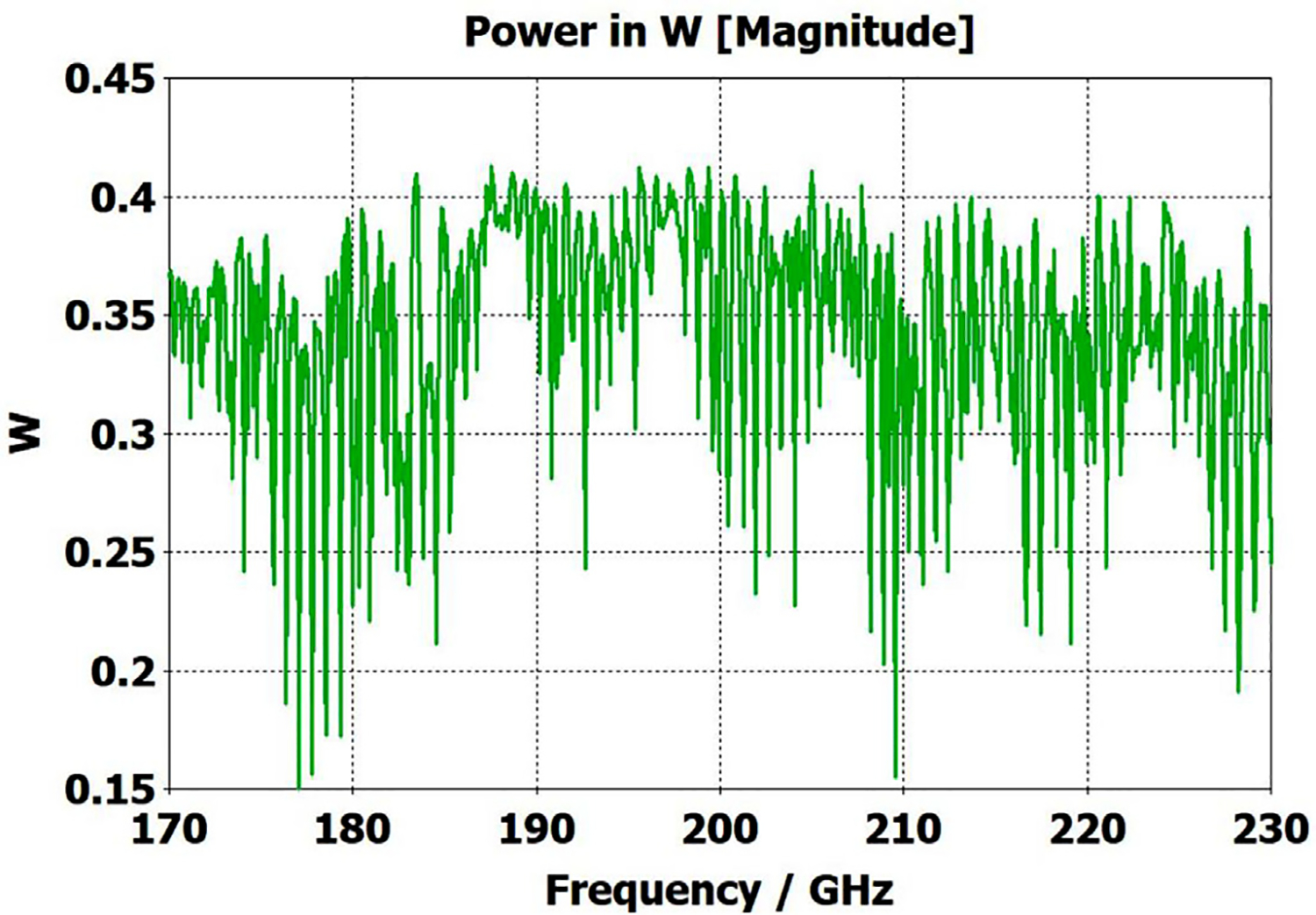
P_A_ at the output for a mid-length model, as pictured in Fig. 33 but longer, with fundamental-mode output (1.5 mm).

The long dielectric slabs on the *x* = 0 plane were replaced with yet more short TLX-9 slabs supporting pairs of copper foil patches, as seen in Fig. 33, and the Teflon linings and two lenses were eliminated. To center the copper foil patches precisely with respect to the *x* = 0 plane, the slabs were modeled as pairs of back-to-back copper-clad 5-mil TLX-9 slabs, with their copper-clad surfaces together on the *x* = 0 plane. The improvement compared to many other attempts to get down to fundamental-mode output after an OMWG, such as in Fig. 32, was dramatic, as seen in Fig. 34, with the improvement coming more from the copper patches than from the other changes.

**Fig. 35.**
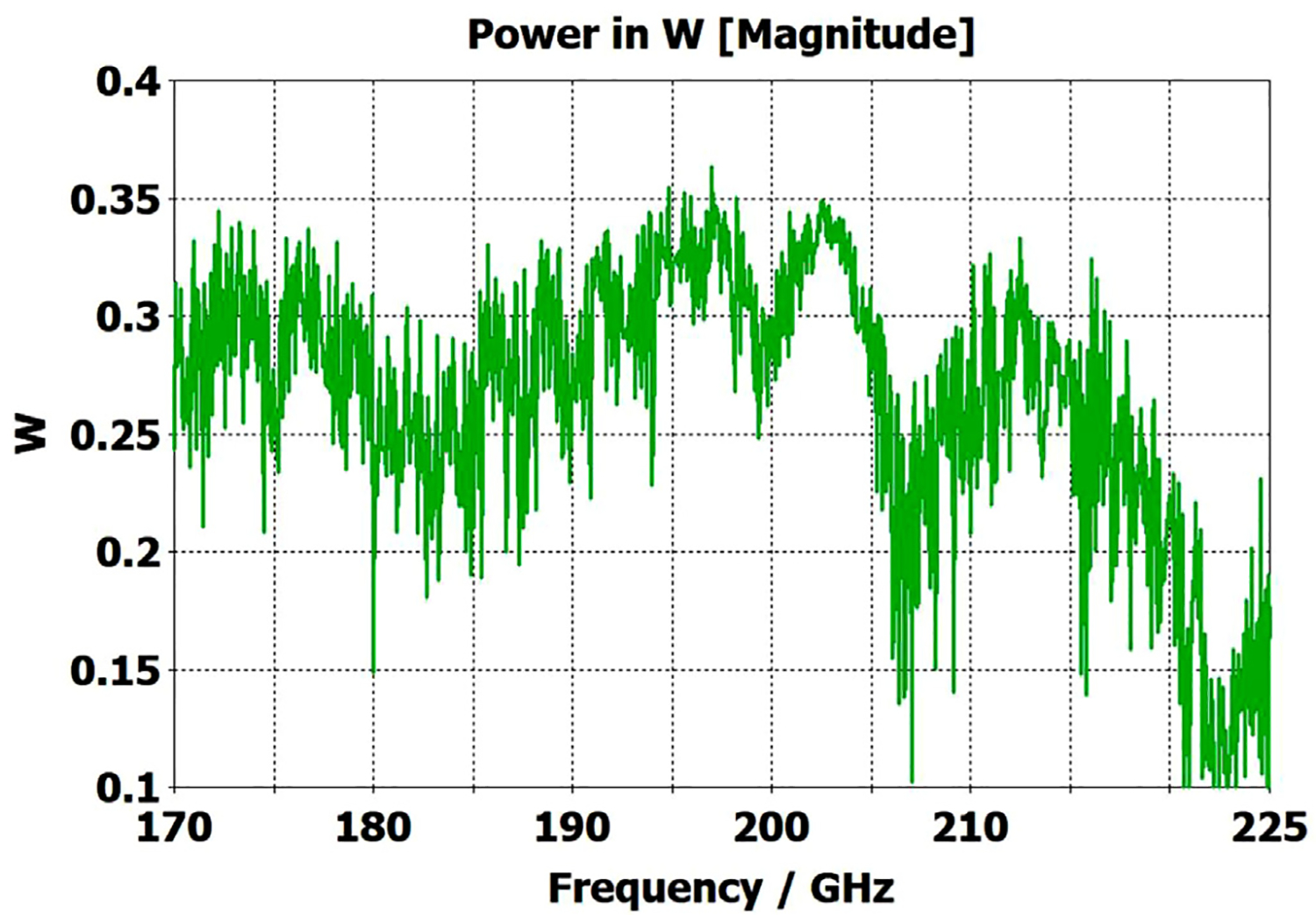
P_A_ at the output for a model similar to that in Fig. 33 but ~5 times longer, with 2.2-mm out-port.

The same method was applied to a much longer version as appropriate for use in a MAS-DNP probe, with the first OMWG six times longer and with the other OMWGs about 50% longer. Numerous parameter sweeps were run to optimize the positions, number, and locations of the copper foil patches, and a few corrugations were added at the beginning of the first OMWG. In the final design (and these models did not include manufacturing errors), there were 8 slabs in the first OMWG, each 3 mm wide, 7 in the second OMWG, each 6 mm wide, and two in the third OMWG, each 3 mm wide, as in Fig. 33. Ultimately, the results shown in Fig. 35 were obtained with 2.2-mm diameter output – similar to what was seen earlier for similar overall dimensions with two corrugated sections in Fig. 20. However, this design did not appear to offer manufacturing advantages over the corrugated alternative.

## 8. The corrugated vs spline-taper horns for going from fundamental-mode TE_11_ to HE_11_

The beam supplied to an NMR-DNP probe, whether from a corrugated waveguide or from an open beam, clearly needs to be HE_11_ or TEM_00_ respectively; but we wish to begin with TE_11_ in standard fundamental-mode waveguide to simplify some bench validation experiments with OMWGs. There are two standard methods for generating HE_11_ from fundamental-mode TE_11_: corrugated horns and spline-taper horns.

**Fig. 36.**
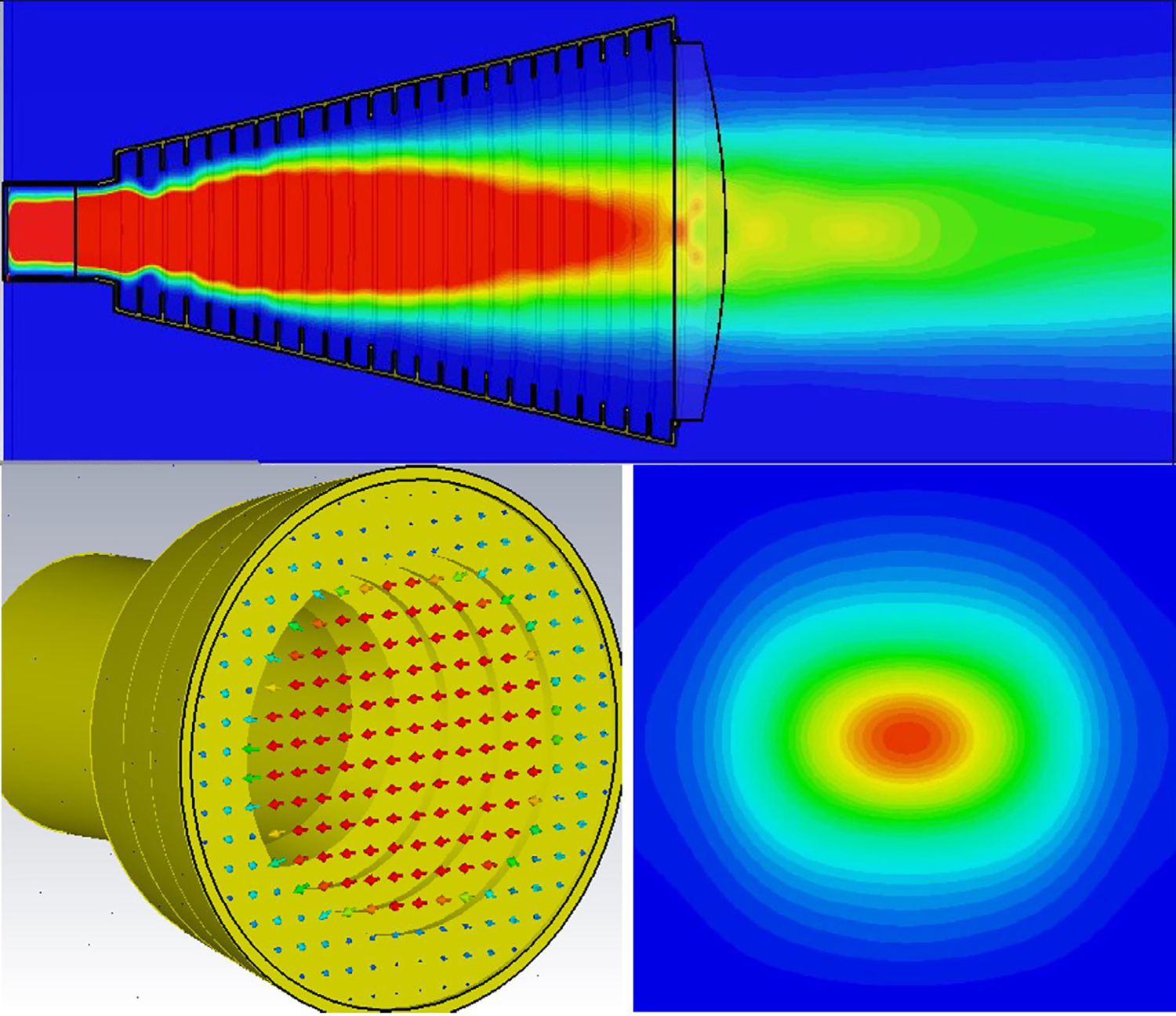
(A) Power flow in the YZ plane (color range clamped, red is 1E5 W/m^2^, blue is 0); (B) E vectors in XY cutting plane just after the fourth tooth showing the field is already HE_11_; and (C) power density in the XY plane at the maximum z boundary, which is 10 mm beyond the lens.

Corrugated horn antennas have been used in communication stations and radio astronomy for nearly half a century because the corrugated horn permits efficient generation of a wide-band highly symmetric radiation pattern with no cross-polarized component and the output field is a highly directional HE_11_ mode with virtually no side lobes (low-amplitude halo beyond the main on-axis beam) [43, 66]. Details of a design optimized for 120–270 GHz were recently published [66], and we used that initially to convert from fundamental mode TE_11_ to HE_11_. This horn is fed from waveguide of radius 1 mm, has a flare (semi-) angle of 14°, an aperture radius of 4.16 mm, a −3 dB far-field beam semi-width of ~7° (at mid-band), a 1/e^2^ full width of 25°, and side lobes below −40 dB. We tried adding a Teflon plano-convex lens at the aperture with 14.5-mm focal length (and weighted-average thickness close to *n λ*/2, where *λ*=*λ*_0_ / *ε*_r_^0.5^) to approximately cancel the mean half-power divergence so as to better approximate the divergence that could be expected from a good quasi-optical beam, but found it didn’t help broadband performance of the waveguides we excited with this HE_11_ source. Three field plots from a CST 0.5 W TE_11_ TD excitation, radiating into space (all open boundaries), are shown in Fig. 36 at 200 GHz.

**Fig. 37.**
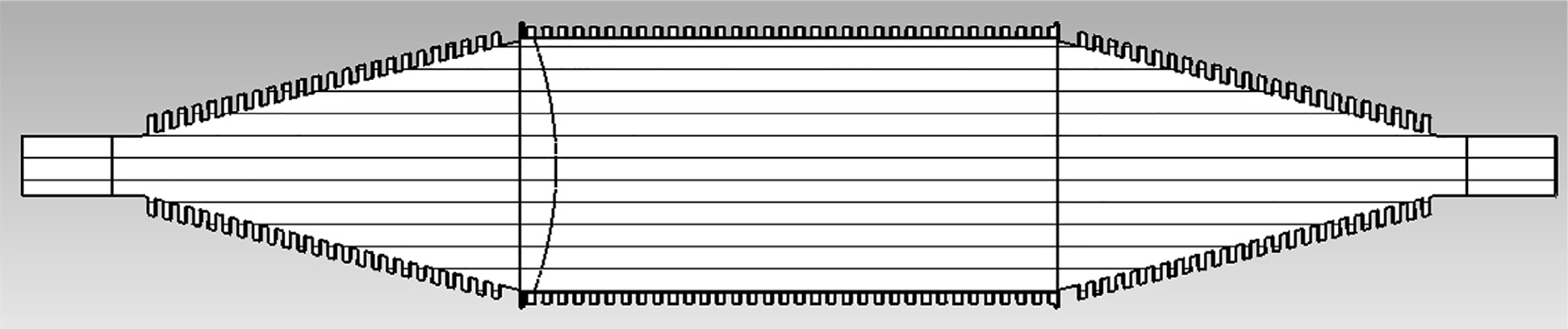
The corrugated lens-horn, excited with TE_11_ from fundamental-mode waveguide, fed the short corrugated 8.5-mm OMWG, followed by a mirror-image downtaper (but no lens) to a 1.5-mm waveguide to the out-port.

**Fig. 38.**
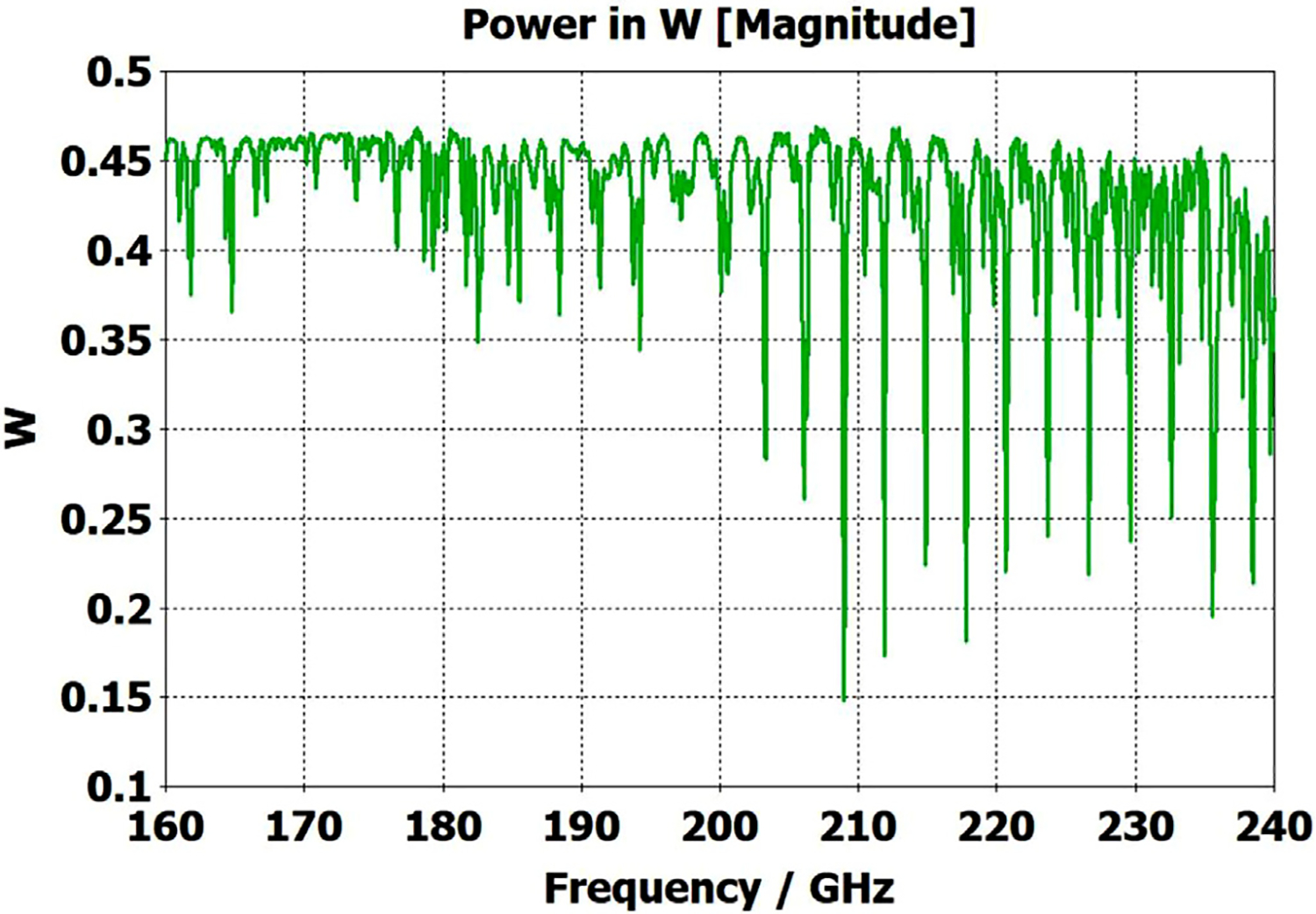
P_A_ at the output port for a short 5-section model essentially as depicted in Fig. 37, going down to fundamental-mode (1.5-mm) out-port.

The above lens-horn, fed with TE_11_ from 1.5-mm waveguide through a small uptaper to 2-mm diameter, was used to excite a short length of corrugated OMWG of 8.5-mm diameter, initially with corrugation groove depths of *λ*/4, periodicity of *λ*/3, relative groove width of 0.4, followed by a corrugated downtaper (mirror of the feed horn) to 1.5-mm waveguide as shown in Fig. 37. The resulting transmission spectrum, shown in Fig. 38 – not an improvement over results from the smooth waveguide of similar central dimensions seen in Fig. 8 – was surprising, as others had used corrugated downtapers and had not reported anything remotely similar.

**Fig. 39.**
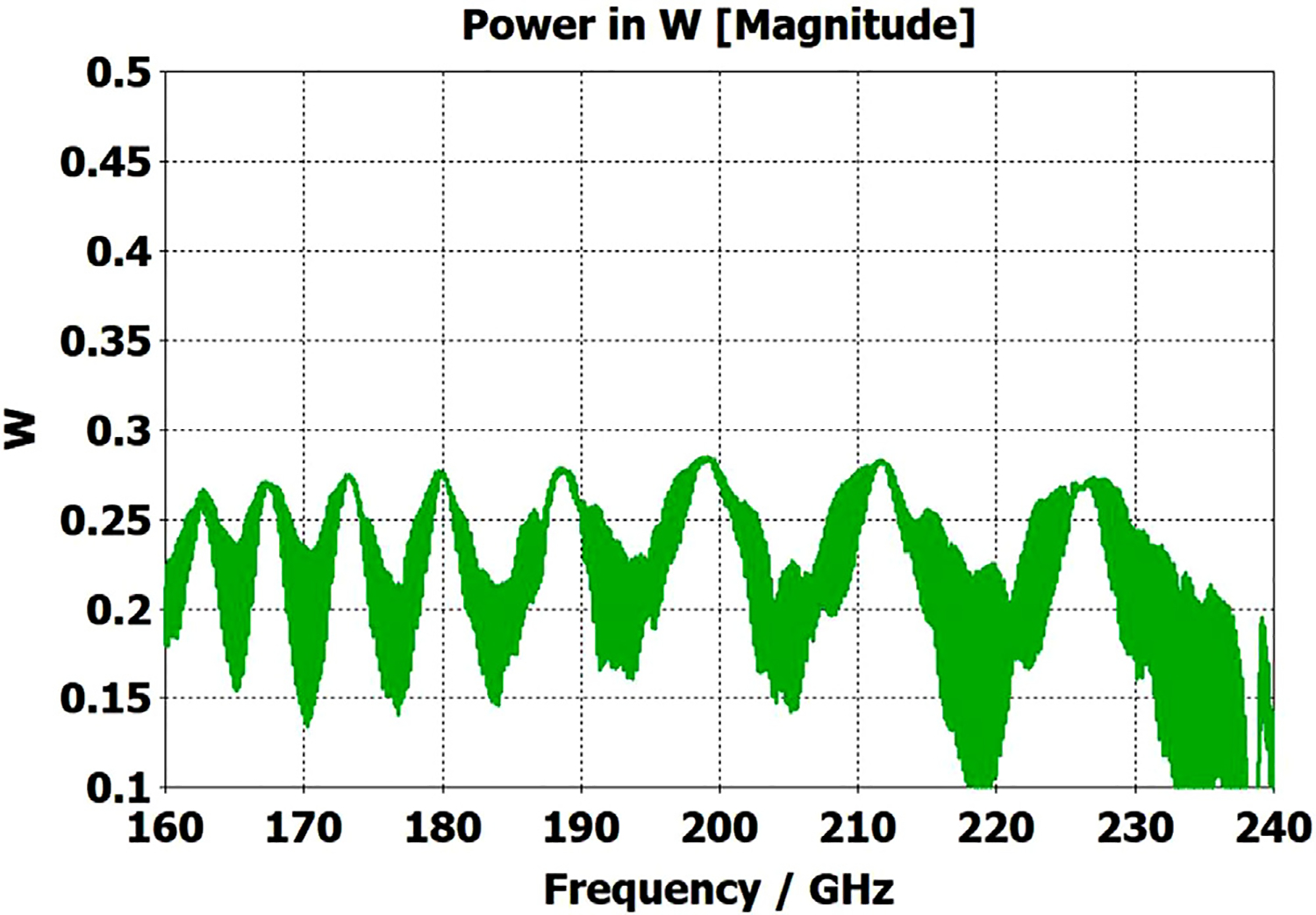
P_A_ for port_2 for a model similar to that in Fig. 38 but with 2-mm out-port diameter, improved corrugation parameters, no lens, and 600-mm length of the OMWG.

We soon saw that much better results were possible if the diameter of the out-port was increased to 2 mm so that TM_11_ – a significant component of the HE_11_ from the source horn – could propagate to the load. (The cut-off for TM_11_ in 2-mm waveguide is 183 GHz.) Some changes in the corrugation parameters also helped a little, and removing the lens helped a little in some cases. However, as the OMWG was made longer the spikes just got closer together, as seen in Fig. 39, for a case with a 600-mm-long OMWG, 33 times longer than pictured in Fig. 37.

### 8.1. Spline horns are preferred over corrugated horns

The corrugated horns pose significant manufacturing challenges by conventional metal machining methods, as noted in the introduction. Thus, over the past decade, the smooth-walled spline-profile horn has been increasingly seen as a preferred alternative in the THz regime to the corrugated horn for generating the HE_11_ beam needed by antennas for low cross-polarization, high gain, large bandwidth, low return loss, low side-lobe level, and small size [67–69]. Several profile optimizations have been published for different prioritizations of the various objectives. While corrugated horns have thus far achieved slightly better overall performance for a given size – at least below ~300 GHz – the spline horns are much easier to produce. Like the corrugated horn, the spline horn begins with a short mode-conversion region, which in the spline horn is achieved by rapid expansion while in the corrugated horn it is accomplished by deep corrugations. Then comes the phase-slip (or smooth intermediate) region designed to achieve the needed phase relationship between the TE_11_ and TM_11_ modes to form the HE_11_. That is followed by the final expansion section, which largely affects the radiation pattern. We chose to follow an optimization that prioritized large fractional bandwidth (~30%), low cross-polarization (<−30 dB), and small relative aperture [68], and we scaled its dimensions from 33 GHz to 200 GHz.

**Fig. 40.**
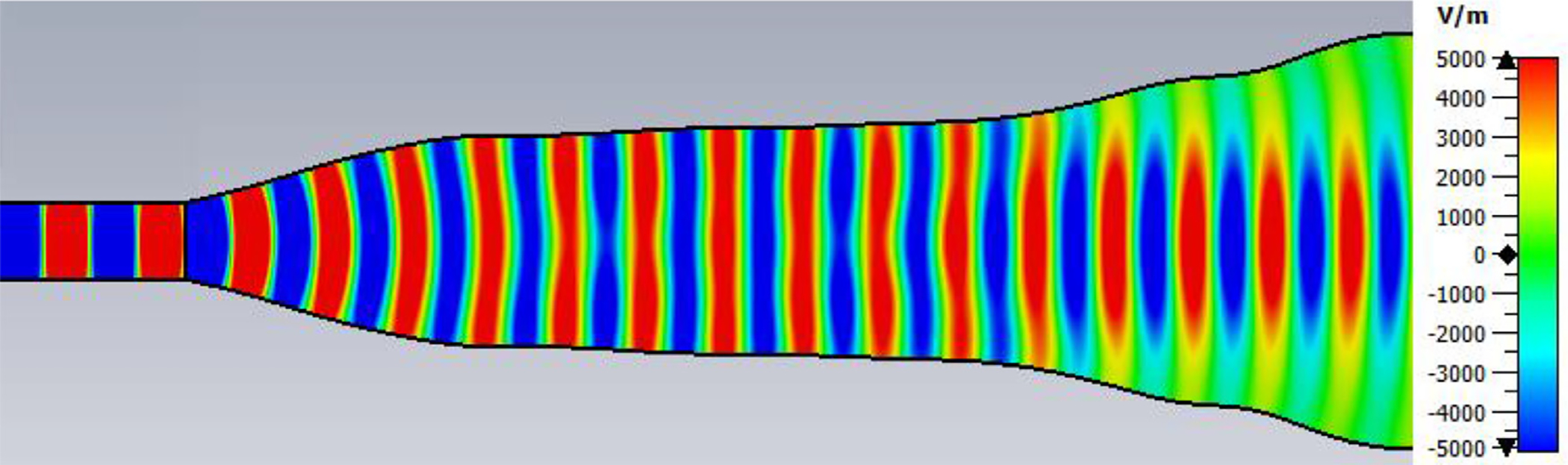
The spline horn, as used in subsequent models, on the XZ plane with Ex at 200 GHz, for going from TE_11_ to HE_11_.

**Fig. 41.**
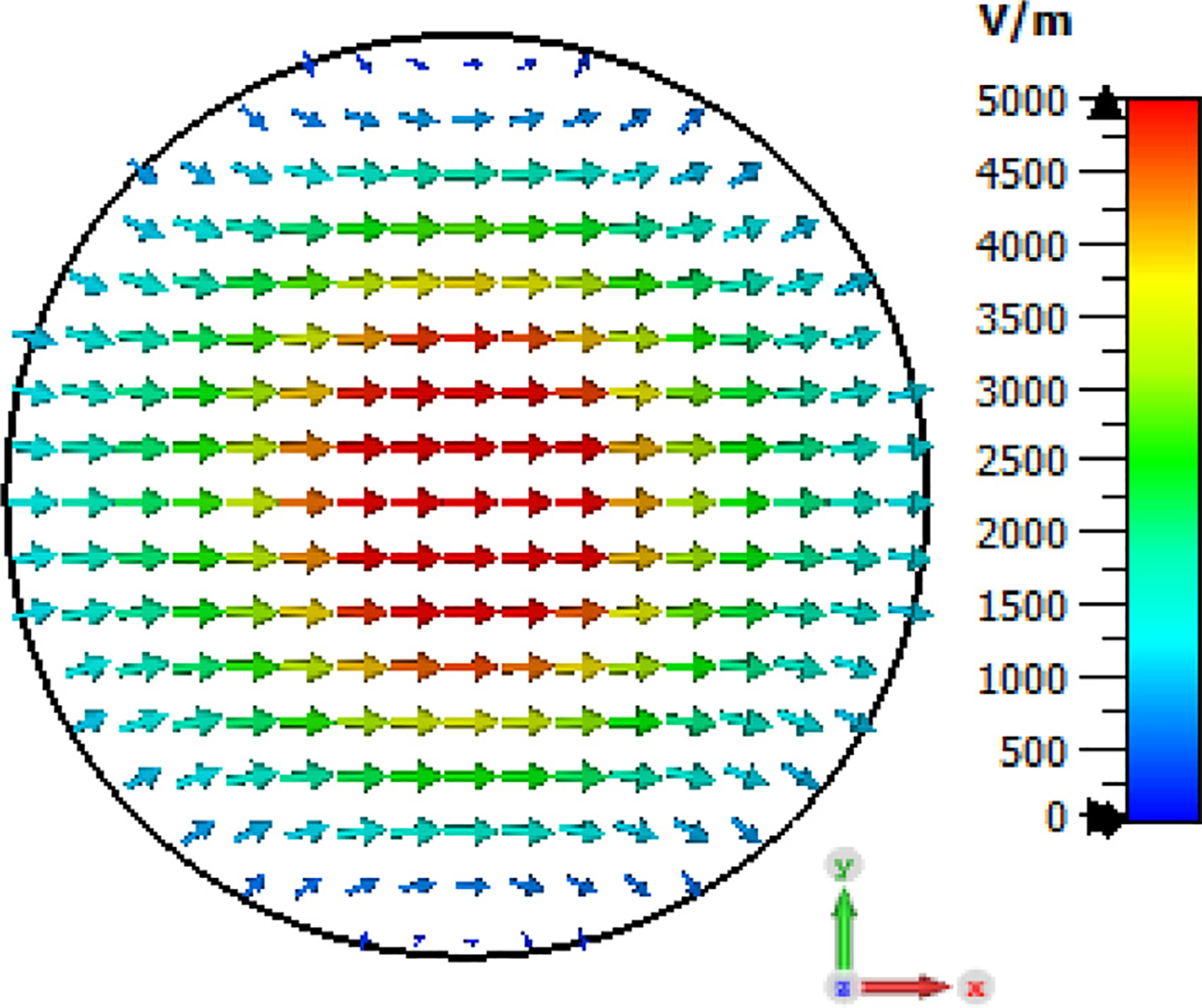
The E field vectors in the XY plane at the aperture for the spline horn and conditions of Fig. 40.

**Fig. 42.**
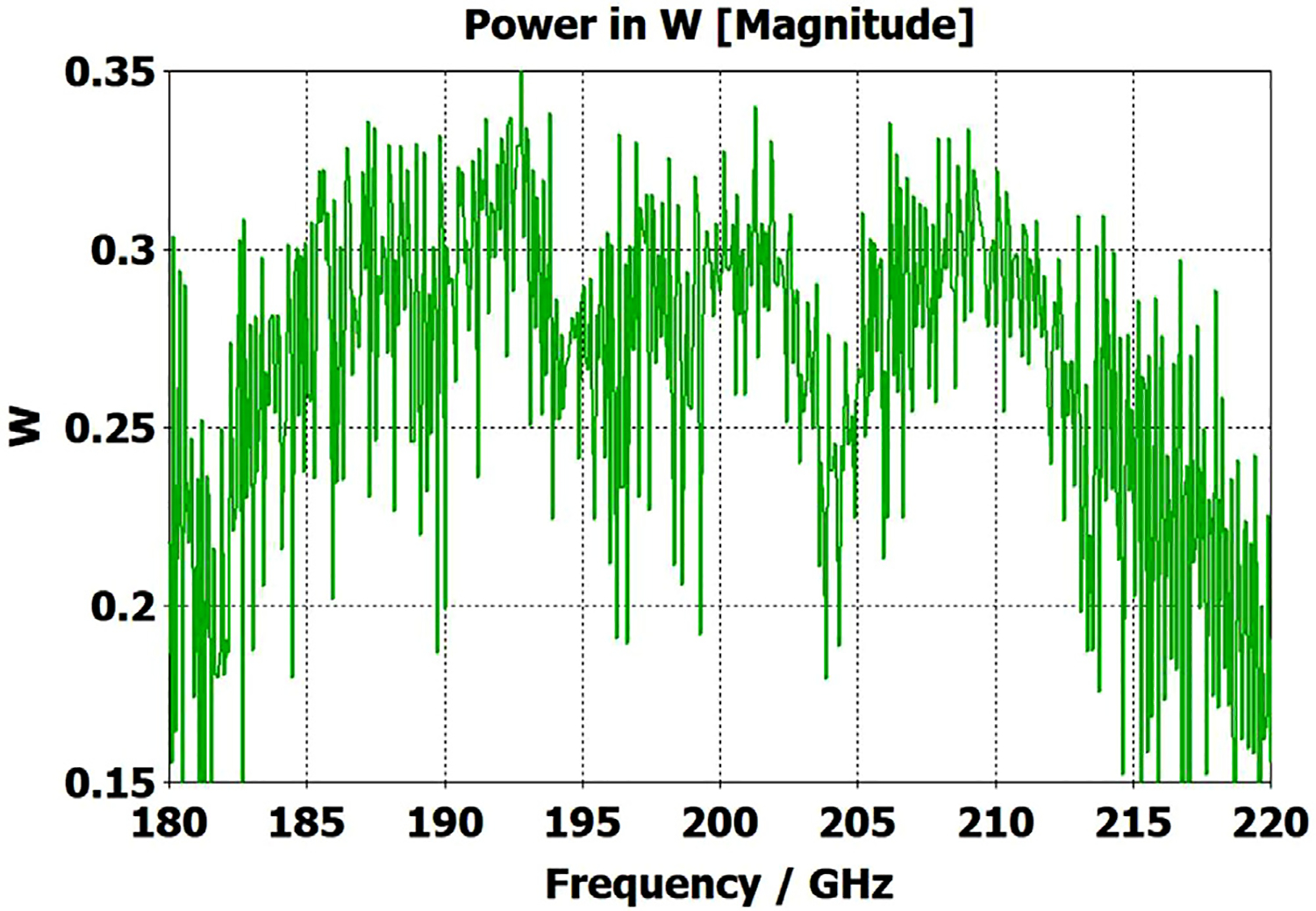
P_A_ for port_2 for a long model very similar to that for Fig. 35 except with the corrugated horn replaced with a spline horn.

The profile of the spline-horn used to excite our 8-mm OMWG with HE_11_ from fundamental-mode TE_11_ (coming from 1.5-mm waveguide) is seen in Fig. 40 with Ex at 200 GHz. The E field at the aperture on the XY plane is shown in Fig. 41. The fields are quite close to ideal HE_11_, and the 3dB bandwidth was >100 GHz. When the corrugated horn in models such as shown in Fig. 33, or its longer version, as used for the results shown in Fig. 35, was replaced with the spline horn of Fig. 40, the overall bandwidth that could be achieved with low loss was similar and the response was smoother, though some of the roughness in the simulations using corrugated horns appeared to be an artifact of the meshing. Fig. 42 shows the port_2 P_A_ results for a long model of overall dimensions the same as those of Fig. 35 with similar internal slabs and foils, no dielectric linings, and no lenses. Sweeps on parameters for the foil patches and their supporting dielectric slabs revealed room for further improvement by reducing their widths and increasing their number, at least in the first OMWG. However, this is even more impractical from a manufacturing perspective.

**Fig. 43.**
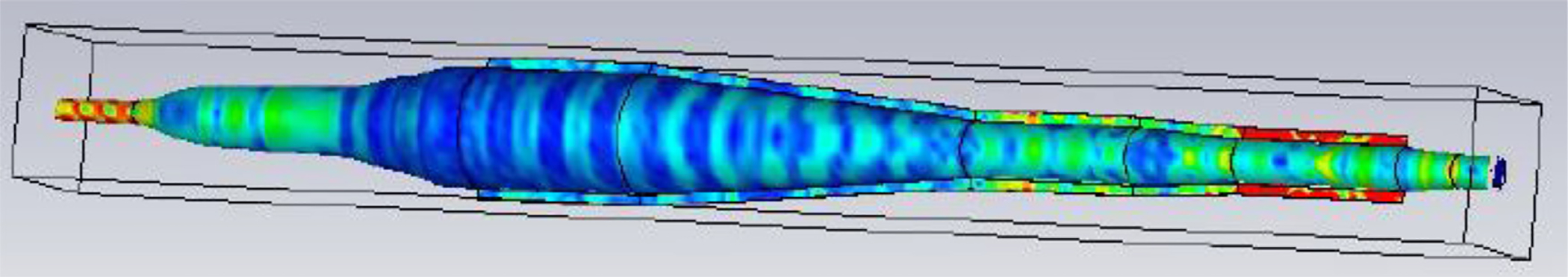
External perspective view showing absolute value of surface current density at 198 GHz on a short slab-bisected model: OMWG lengths of 12 mm, 15 mm, and 15 mm. Red is > 30 A/m.

A possibly better alternative to explore appeared to be bisecting nearly the full length of the waveguide, from the beginning of the first OMWG to the end of the third OMWG, with a long thin dielectric slab along the YZ plane to support small copper patches wherever desired on the *x* = 0 plane. As before, this was a pair of back-to-back copper-clad TLX-9 slabs so the copper patches could be precisely centered with respect to the *x* = 0 plane. This slab-pair was supported by grooves 3*λ*_d_/4 deep into the walls of the waveguide, where *λ*_d_ is the wavelength in the substrate at 200 GHz. An external perspective view showing absolute value of surface currents at 198 GHz for a short slab-bisected model, again with three downtapers, is portrayed in Fig. 43.

**Fig. 44.**
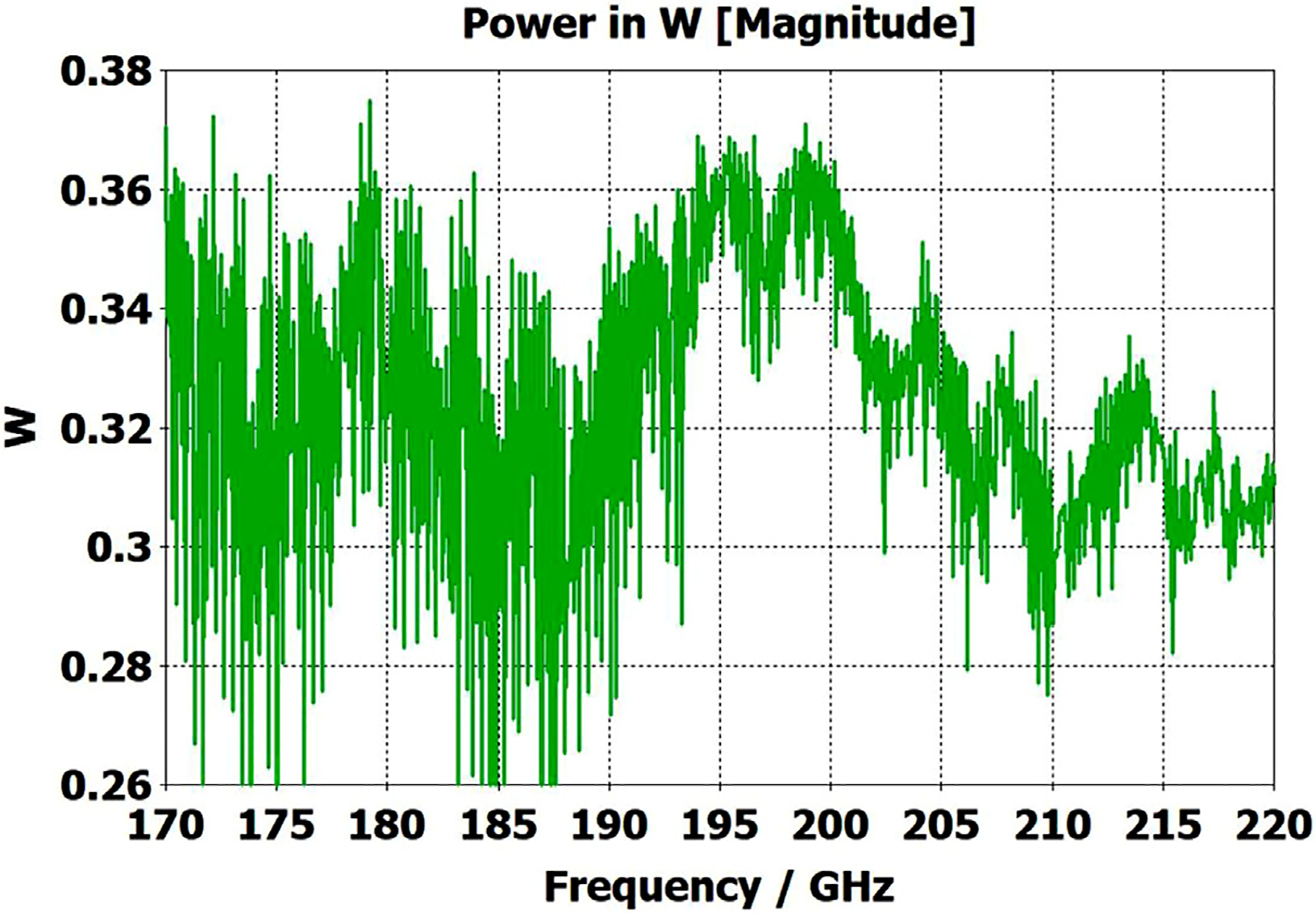
P_A_ for port_2 for a long model of the slab-bisected smooth waveguide similar to that seen in Fig. 43, but with OMWG internal dimensions the same as for the models for Figs. 20, 35, and 42: OMWG lengths of 357 mm, 135 mm, and 22 mm, diameters of 8.1 mm, 3.85 mm, and 3 mm, and outport diameter of 2.2 mm.

Fig. 44 shows the generally improved broadband port_2 P_A_ for a slab-bisected model with the same overall dimensions as those for Fig. 42 and noted in the caption, with no dielectric linings or lenses and optimized copper patches on the *x* = 0 plane, that were now much smaller, but still killing primary cross-polar modes. Comparing the results here to the corrugated case in Fig. 20 (which had the same internal dimensions in the OMWGs, and similar downtapers), one sees rather similar overall mean results. The graph here is much more ragged than Fig. 20, but that is because the model for Fig. 44 begins with a horn that reflects most of what comes back to it (thus giving rise to many more trapped modes) rather than a full-diameter ideal HE_11_ port that absorbs everything that comes back to it. However, there is another important difference: the model for Fig. 20 included rather large asymmetric defects, while the model for Fig. 44 does not.

A closer look at several details reveals there are issues with the slab-bisected design that will probably keep it from being competitive with numerically optimized corrugated designs. Foremost may be the sensitivity that was seen of performance to small errors in the fit of the slab to the grooves in the wall that hold the slab in position – because of the intense fields there in places, as is obvious from the plot of surface currents in Fig. 43. That also means even thin contaminating films there could greatly increase losses.

**Fig. 45.**
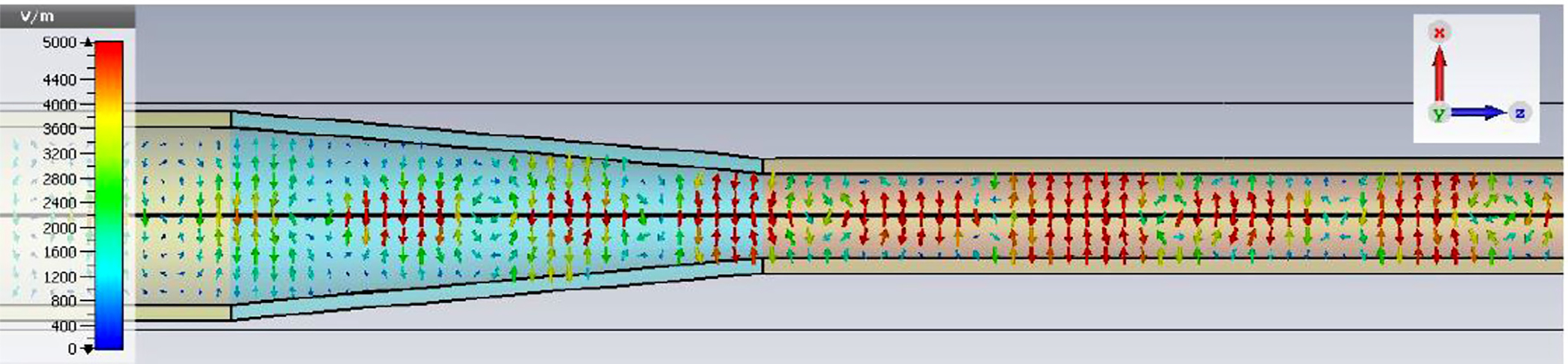
E field in the model for Fig. 44 at 198 GHz in the first downtaper and into the second OMWG on the XZ plane. Red is >5000 V/m.

The E-field plot for the above model at 198 GHz, as depicted in Fig. 45 for the first downtaper and into the second OMWG on the XZ plane, shows fields near the surface are very high in many places, even at a frequency where loss is minimal, which makes this design quite sensitive to manufacturing defects. We also worried about the mechanical stability of the TLX-9 slab on the *x* = 0 plane that was made unrealistically thin (0.06 mm) to minimize losses in it. It was at this point that we began looking at improving performance of small corrugated OMWGs, as discussed in an earlier section, and yet other creative alternatives, a subject we expect to return to at some point in the future; but we turn now to bench experiments with OMWGs using fundamental-mode instruments, as discussed next.

## 9. Experimental validation of CST TD simulations of multiple overmoded transitions

**Fig. 46.**
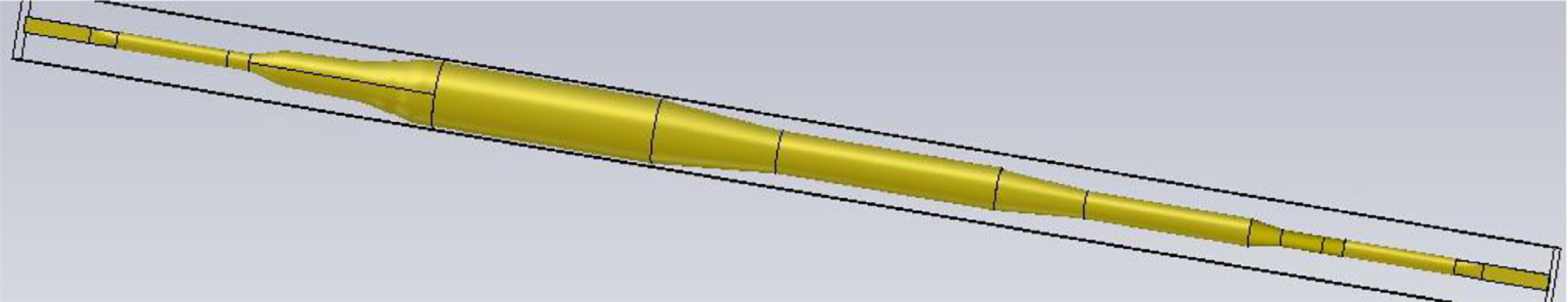
The experimental model, beginning and ending with TE_11_ in rectangular waveguide.

For simplified quantitative bench validations of simulations of OMWGs and transitions, it may be best to start with TE_11_ in standard fundamental-mode waveguide, end with TE_11_ in standard fundamental-mode waveguide, and put everything in between into the simulation – in a model that is not too difficult to manufacture and that doesn’t generate the kind of fine structure that is not likely to be reproduced in the experiment. A perspective view of the outside of the air space in the experimental model is shown in Fig. 46. It begins with a standard rectangular to circular converter (Cernex CRC050058, 140–220 GHz).

**Fig. 47.**
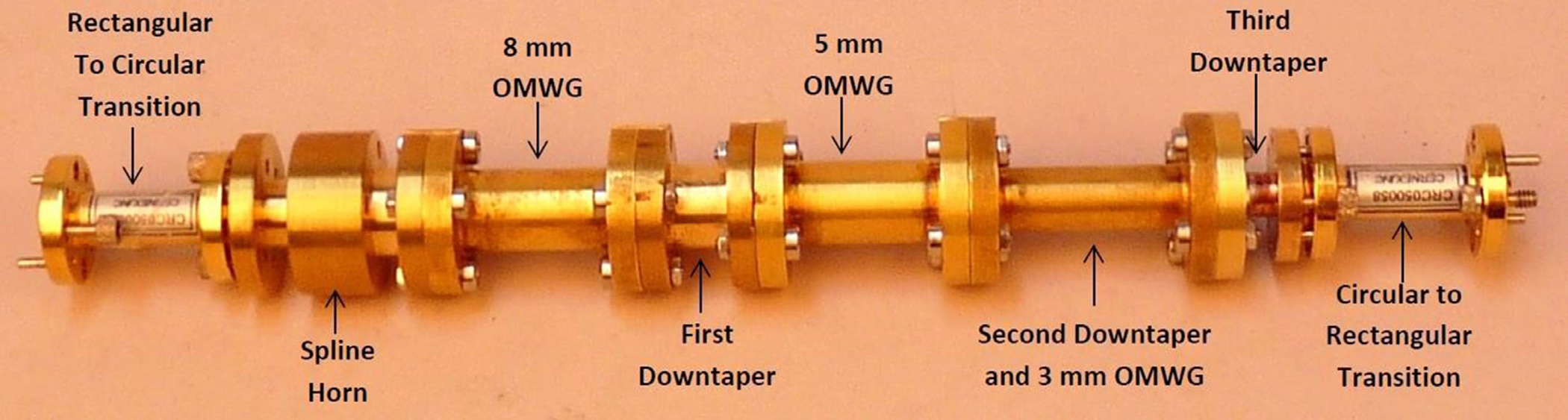
Photo of the experimental hardware setup. Clearly, the external features of the hardware bear little resemblance to the internal features, other than section lengths.

It then proceeds as shown with fundamental mode (1.5 mm) circular waveguide, followed by a spline-profile horn into a short OMWG, a smooth downtaper, a second OMWG, a second down taper, a third OMWG, a final downtaper to fundamental mode circular waveguide, and ends with the same (reversed) Cernex converter into rectangular waveguide. The Cernex converters have a small transverse foil in the circular section a short distance from the loft for suppression of cross-polar modes, and that was included in the model. The rest of the model is simply smooth hollow metal, with no internal slabs, foils, linings, or lenses. A photo of the experimental bench setup in seen in Fig. 47. The source was a Millitech PLS-05-A-002 187–211 GHz 2 dBm Phase Locked Synthesizer, followed by a Millitech FBI-05-RSES0 isolator and then an Elva-1 DC-05/20 directional coupler. An Elva-1 DC-05/6 directional coupler was also used on the receiving end. The detectors were Millitech DET-05-RPFW0, 140–220 GHz WR-05.

The spline horn was the same as used in earlier simulations, scaled from the Wollack design [68]. The OMWG lengths were 30 mm, 30 mm, and 22.5 mm, with diameters of 8.1 mm, 5.1 mm, and 3.0 mm respectively. The downtaper semi-angles were 5°, 5°, and 10° respectively. (Smaller angles on the final downtaper made no significant difference in the overall performance quality.) There were unavoidable small alignment errors (typically ~0.04 mm) at the flanges between the various components, and small fillets (typically ~0.05 mm) at those junctions. Those features were measured to within ~0.02 mm and an attempt was made to include them in the model.

The mean measured loss through just the Cernex convertors with the rest of the model removed was ~3.2 dB over the 190–210 GHz range and fairly flat. The surface conductivity in the model of the convertors was adjusted to the surprisingly small value of 9E5 S/m so that CST simulation results were similar to experimental results. The conductivity in the rest of the model was kept at 1.5E7, as believed to be appropriate for typical gold platings more than 3*δ* thick at 200 GHz.

**Fig. 48.**
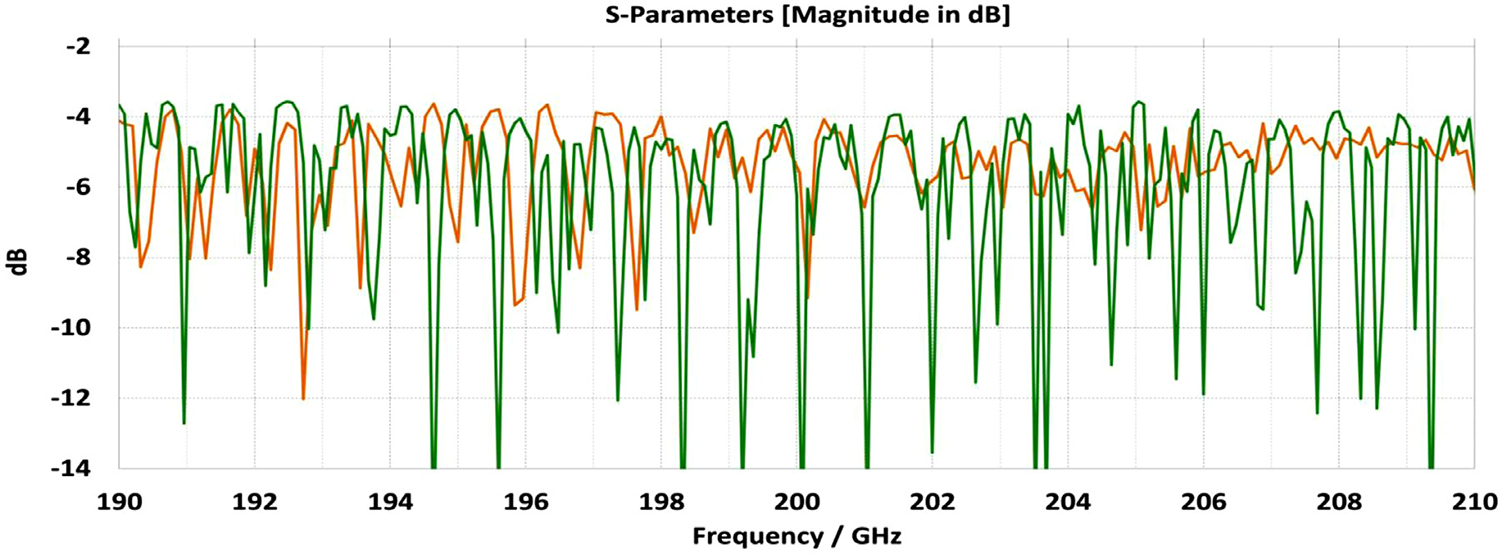
S21 for the experimental model as measured (in red) and as simulated by CST (green).

**Fig. 49.**
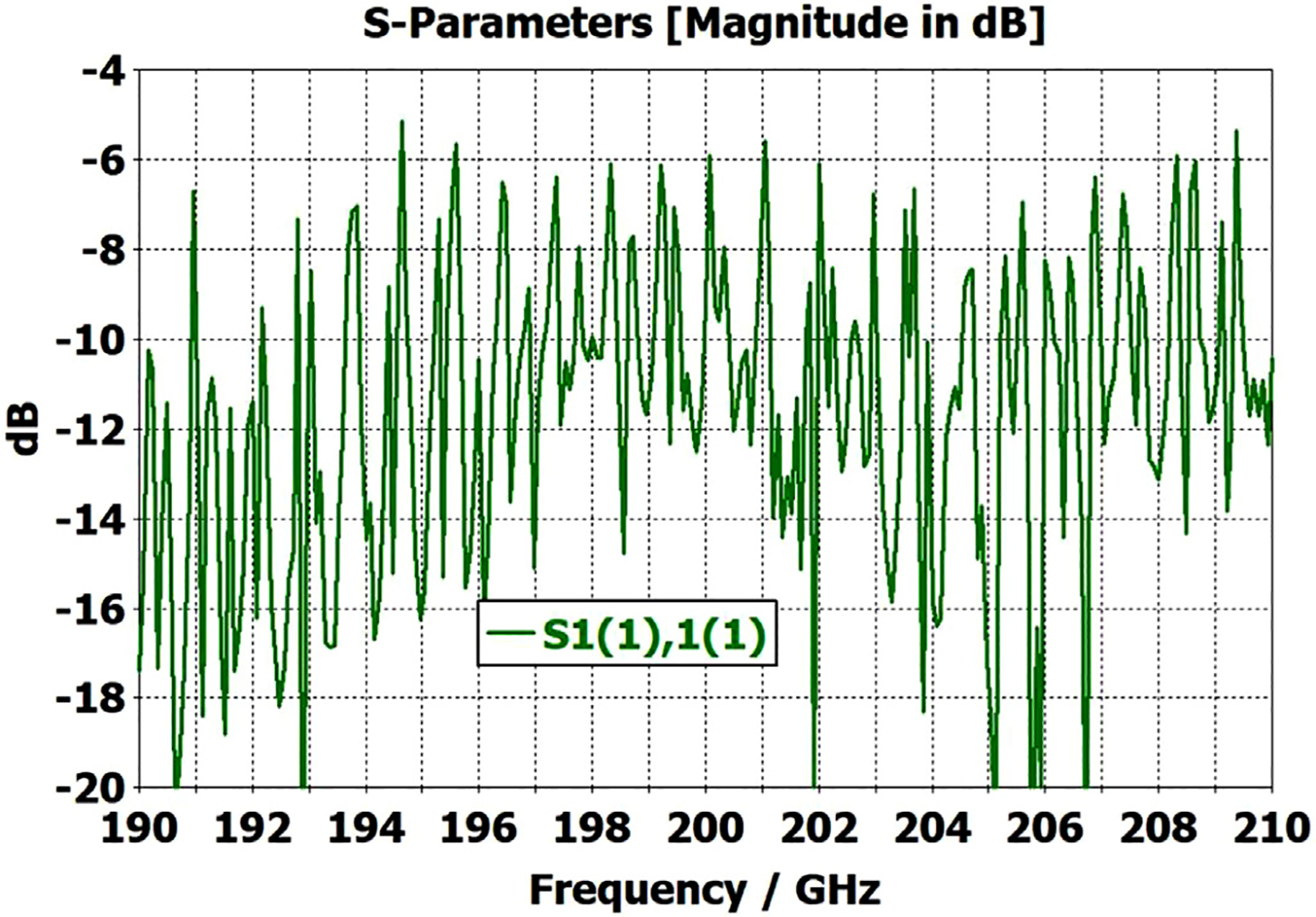
S11 for mode 1 from the CST simulation of the experimental model.

The simulation S21 results are shown in Fig. 48 (from a run with no symmetry planes, 39 M mesh cells) along with the measured results. (The CST results with two symmetry planes were similar to those with no symmetry planes.) The S11 plot from CST in Fig. 49 shows significant reflected power even at the best transmission frequencies, and the experimental results (not shown) while differing in details, were roughly similar.

**Fig. 50.**
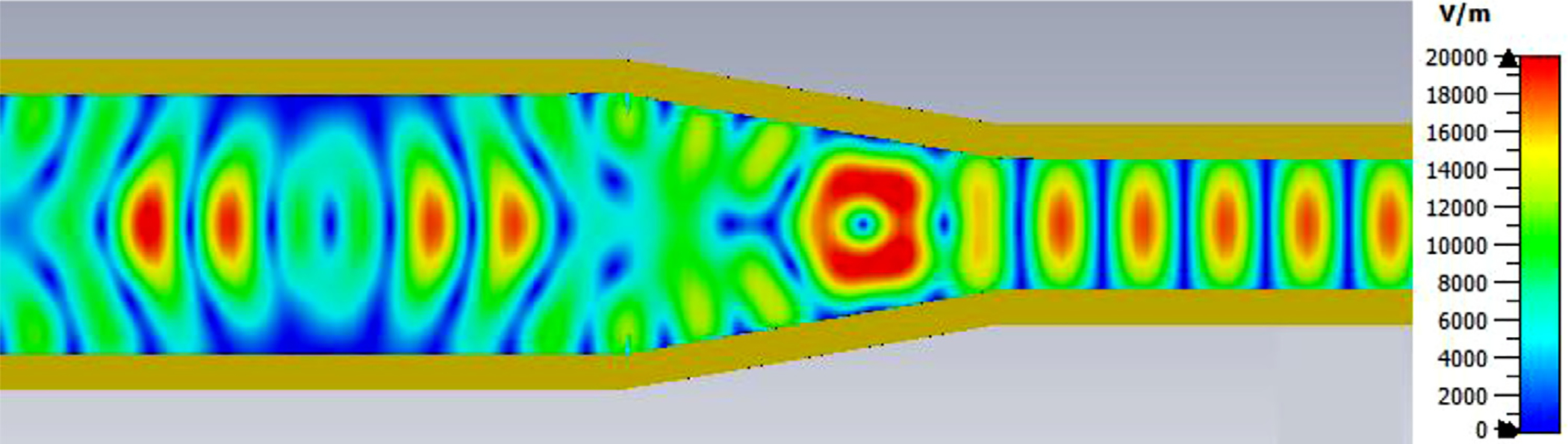
Eabs in the final downtaper of the experimental model at a frequency of very good transmission.

**Fig. 51.**
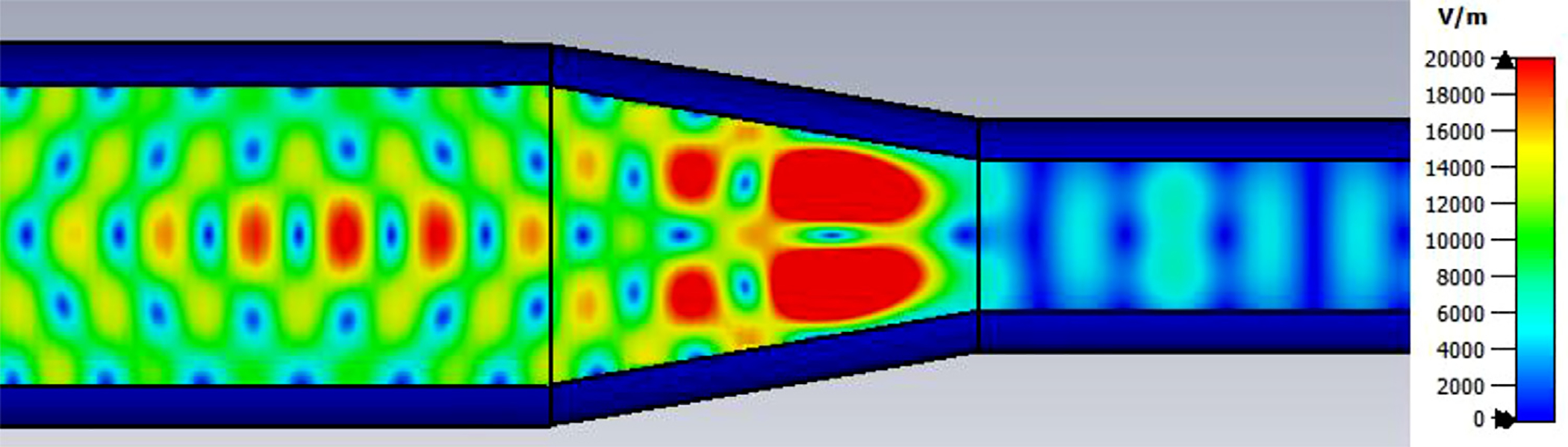
Eabs in the final downtaper of the experimental model at the frequency of a trapped mode.

Fig. 50 shows a snapshot of Eabs in a cross-section near the final downtaper at a frequency of good transmission between trapped modes, and Fig. 51 shows the same at the frequency of a trapped mode nearby. Most of the losses in the trapped mode were seen to be on the surface of the final small (3-mm diameter) OMWG.

The experimental mean separation between absorptive spikes in the central region (195–202 GHz) was ~0.87 GHz, quite close to the ~0.9 GHz seen in the CST simulations, though they showed splitting of many of the modes that was not seen experimentally. The differences in detail features of the two transmission spectra are probably mostly due to alignment errors and effects of the imperfect junctions between the various components.

## 10. Concluding remarks

Here we have considered a set of common methods of improving the performance of OMWG for transmission of Terahertz radiation for NMR-DNP. Our purpose was to provide a practical guide of dos and don’ts of designing OMWG rather than give a solid explanation of why the rules we outlined work. We hope future theoretical studies focusing on individual techniques will achieve the latter. In the mean time a brief summary of the former is given below.

The strong resonances seen in the various cases that began and ended with fundamental-mode waveguide and had an overmoded section in between were not surprising. The TE_11_ wave entering the horn or uptaper is partly converted to TM_11_ near the initial flare, and those modes then proceed at different velocities. At some frequencies, they arrive at the end of the final downtaper with a field structure that doesn’t closely resemble a mode that can propagate in the final waveguide (TE_11_, TM_01_, or TE_21_ below 220 GHz in 1.5-mm waveguide for example) so the wave gets reflected. When it gets back to the throat of the horn much of it again will likely be of a form than doesn’t propagate in the source waveguide so again it gets reflected, and absorptive modes build at those frequencies if there is no internal structure to suppress them or help convert them to propagating modes at the final downtaper.

The results from many of the cases simulated did surprise us. HE_11_ - beam-driven defect-free corrugated waveguides with corrugations according to prior recommendations performed well, but when defects and downtapers were added, performance could be significantly improved with numerically optimized corrugation parameters. In these cases, we saw better results from multiple short downtapers than from a single long downtaper to the same final size, and corrugated downtapers did not generally perform better than smooth downtapers in the cases we simulated. We were quite surprised to see that a HE_11_-beam-driven defect-free smooth OMWG with multiple downtapers performed remarkably well – better than a similar corrugated case with defects.

Our simulations of straight long sections of corrugated waveguides with *a*~4*λ* gave attenuation close to that calculated from published expressions, but we saw significantly greater attenuation than expected (both from reported experimental results and from analytical expressions) for larger diameters and somewhat less than expected for smaller diameters. However, the bigger surprise came for short sections, where attenuation in a case where length was just 5 *d* was seen to be more than an order of magnitude greater than expected.

We did not find various previously reported non-corrugated methods (including dielectric linings and lenses) for improving transmission performance of overmoded waveguides to be beneficial, except perhaps with very short waveguides. Several novel planar internal structures were found to be beneficial in improving flatness of response and mean transmission performance, particularly when the final waveguide was large enough for TM_11_ to propagate. In these cases, we generally saw better (smoother) performance when the initial expansion was a spline horn rather than a corrugated horn. However, the novel structures reported here all appeared to present serious manufacturing and stability challenges.

We have not given up in our search for a better option than corrugated waveguides for cases where size or cost constraints weigh strongly against them and other known options also appear to be unsatisfactory for one reason or another. We expect our progress on a novel promising alternative to be the subject of a future report.

## Figures and Tables

**Table 1:** Modes in circular WGs of 6-mm ID at 200 GHz.

Mode	*χ*	*f*_c_, GHz	v_g_/*c*	*λ*_g_, mm	Z, Ω	*α*, dB/m

TE11	1.841	29.3	0.989	1.515	381	1.122
TM01	2.405	38.3	0.982	1.527	384	2.164
HE11	2.405	38.3	0.982	1.527	384	1.042
TE21	3.054	48.6	0.970	1.545	388	2.106
TE01	3.832	60.9	0.952	1.574	396	0.256
TM11	3.832	60.9	0.952	1.574	396	2.230
HE21	3.832	60.9	0.952	1.574	396	
TE31	4.201	66.8	0.943	1.590	400	3.083
TM21	5.136	81.7	0.913	1.642	413	2.327
HE31	5.136	81.7	0.913	1.642	413	
TE41	5.317	84.6	0.906	1.654	416	4.126
TE12	5.331	84.8	0.906	1.655	416	0.602
TM02	5.52	87.8	0.898	1.668	419	2.464
HE12	5.52	87.8	0.898	1.668	419	
TM31	6.38	101.5	0.862	1.740	437	2.464
